# Investigating the rationale for COPD maintenance therapy prescription across Europe, findings from a multi-country study

**DOI:** 10.1038/s41533-023-00334-x

**Published:** 2023-05-03

**Authors:** Janwillem Kocks, António Jorge Ferreira, Per Bakke, Onno C. P. van Schayck, Heikki Ekroos, Nikolaos Tzanakis, Stéphane Soulard, Monika Haaksma-Herczegh, Montserrat Mestres-Simon, Malena Águila-Fuentes, Didier Cataldo

**Affiliations:** 1 General Practitioners Research Institute, Groningen, The Netherlands; 2 Observational and Pragmatic Research Institute, Singapore, Singapore; 3 Groningen Research Institute for Asthma and COPD (GRIAC), University of Groningen, University Medical Center Groningen, Groningen, The Netherlands; 4 Department of Pulmonology, University of Groningen, University Medical Center Groningen, Groningen, The Netherlands; 5 Faculty of Medicine, University of Coimbra, Coimbra, Portugal; 6 Pulmonology Department, Coimbra Hospital and University Centre, Coimbra, Portugal; 7 Department of Clinical Science, University of Bergen, Bergen, Norway; 8 Care and Public Health Research Institute, Maastricht University, Maastricht, The Netherlands; 9 Department of Pulmonary Medicine, Porvoo Hospital, Porvoo, Finland; 10 Department of Thoracic Medicine, University Hospital of Heraklion, Medical School, University of Crete, Crete, Greece; 11 Boehringer Ingelheim B.V., Amsterdam, The Netherlands; 12 Adelphi Targis S.L, Barcelona, Spain; 13 Department of Respiratory Medicine, Centre Hospitalier Universitaire de Liège (CHU) and University of Liège, Liège, Belgium

**Keywords:** Chronic obstructive pulmonary disease, Drug regulation

## Abstract

This study aims to understand healthcare professionals’ thoughts and motivations about optimal management and treatment of patients with chronic obstructive pulmonary disease (COPD). We conducted a DELPHI survey through an online questionnaire distributed to 220 panellists from six European countries and a discrete choice experiment to describe the relationship between selected clinical criteria and the initial COPD treatment of choice. One hundred twenty-seven panellists (general practitioners [GPs] and pulmonologists) completed the survey. Despite the familiarity and use (89.8%) of the GOLD classification for initial treatment selection, a frequent use of LAMA/LABA/ICS was noted. In fact, panellists agreed that inhaled corticosteroids (ICS) are over-prescribed in the primary care setting. Our study showed that GPs felt less confident than pulmonologists with ICS withdrawal. This mismatch observed between best practice and behaviour indicates the need to increase awareness and efforts to improve the adherence to guidelines in clinical practice.

## Introduction

Chronic obstructive pulmonary disease (COPD) is the third leading cause of death worldwide. Its prevalence is estimated to be ~10.1%^1^. The global COPD burden both economic and social^2,3^ is substantial and is expected to increase further due to the ageing population and continued exposure to COPD risk factors^4^.

The international COPD treatment strategy document—the Global Initiative for Chronic Obstructive Lung Disease (GOLD)—proposes solutions for initial and follow-up therapy. However, GOLD influence on specific treatment guidelines and on clinical practice varies between countries^5,6^. The GOLD document recommends bronchodilators as baseline therapy and initiation with dual bronchodilators for highly symptomatic patients. It proposes a model for maintenance treatment initiation based on the ABCD assessment, which reflects symptom burden and risk of exacerbation. Treatment recommendations vary between short and long-acting bronchodilators, long-acting muscarinic antagonist (LAMA) or long-acting ß2-agonist (LABA), or various combinations including LAMA/LABA or LABA/ICS depending on the GOLD patient category (A, B, C or D). ICS use for initial maintenance therapy is restricted to patients with previous exacerbations and high blood eosinophil counts in combination with LABA. Although ICS use is also restricted in the follow up therapy, a significant proportion of COPD patients in Europe are still prescribed ICS based on habits and past practice^7^. As patients with COPD often have concomitant chronic illnesses at the time of diagnosis^8,9^, these deserve consideration in the management and treatment choices of COPD^10^. While blood eosinophil counts have been introduced in the recent GOLD document as a way to guide treatment decisions, the effect on prescribing behaviour is still unknown.

While current COPD management and prescription, as well as the overuse of ICS are well described in Europe^11–17^, there is limited knowledge on the healthcare professionals’ motivations for the treatment decision. This study aims to understand general practitioners’ (GP) and pulmonologists’ rationale about COPD care from a holistic perspective.

## Methods

This study surveyed general practitioners (GPs) and pulmonologists across six European countries (primary and secondary care) including descriptive questions on current clinical practice, a discrete choice experiment to identify patient profiles associated with specific initial treatment choices and an eDELPHI component to elicit consensus on treatment decisions.

A total of 220 panellists accepted the invitation to participate in the study. Panellist recruitment followed different methodology in different countries: In Finland and Greece invitations were sent out randomly from the Adelphy Targis panellists database, in The Netherlands by the steering committee members (the authors of this paper), who invited GPs without a special interest in respiratory diseases and non-academic pulmonologists, in Portugal and Belgium by representatives of the company Boehringer Ingelheim B.V. and in Norway by the steering committee members, IPCRG members and Boehringer Ingelheim B.V. representatives. Panellists current work was mainly in public centres (58%), specifically in a non-university hospital (39%) and in private centres (19%), other 23% worked in both public and private centres.

Panellists were GPs and pulmonologists who were required to have over 3 years of experience in their specialty and to have seen a minimum of ten COPD patients in the last month. The questionnaire was distributed online in two rounds between July and December 2020.

Results were analysed in the entire group, by specialty and by country for all questions. An additional sub-analysis by age of the contributing physicians was performed in some questions. Results (total sample and sub-specialty results) in which significant differences were found are reported in this publication, while results by country or age are reported in Supplementary Information 1. Sub-analysis by specialty where no statistically significant differences were found is also detailed in Supplementary Information 1.

**Fig. 1:**
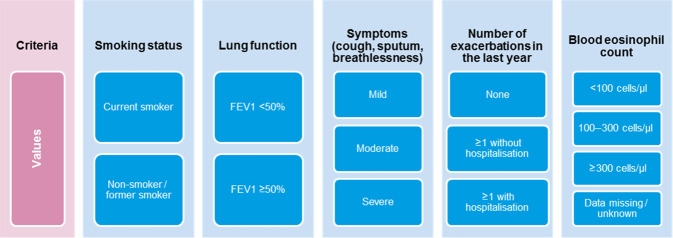
Criteria and values used for the discrete choice experiment. FEV forced expiratory volume.

The discrete choice experiment was performed to assess factors influencing initial COPD maintenance treatment. For this, a set of criteria and values were defined (Fig. 1), resulting in 144 different patient profiles. Each panellist was asked to assign the initial maintenance treatment they would prescribe to 12 randomly selected patient profiles, choosing among LAMA, LAMA/LABA, LABA/ICS, LAMA/LABA/ICS (Triple Therapy), or another alternative.

The analysis of the discrete choice experiment of patients’ profiles included: (1) a univariate analysis to describe the relationship between each one of the defined criteria and the initial COPD treatment of choice; (2) a multinomial logistic regression model to analyse the relative weight of each criterium and their corresponding values of the patient profile on the decision of the initial COPD maintenance treatment that should be prescribed.

A Boolean literature search in PubMed using the search terms “COPD” AND “management”, OR “maintenance treatment” OR “inhaled corticosteroids” was first performed to design a first version of the eDELPHI questionnaire. Then, based on their knowledge and experience in COPD, all authors iteratively reviewed the questionnaire, agreed on amendments and validated the final version of the questionnaire (Appendix 1). The questionnaire contained descriptive questions and consensus questions (9-level Likert-type scale). Consensus on agreement or disagreement was pre-defined before data collection and set as 70% or more experts coinciding on the assessment in a 9-levels Likert scale recorded in three categories (1–3 disagreement, 4–6 neutral, 7–9 agreement). All results of the first round were analysed. Those questions where no consensus was reached, were asked again in the second round of the study, showing the results obtained in the first round. Only the answers of the panellists who fully completed the eDELPHI study were analysed and considered valid. Nominal variables were described by frequency counts and percentages. For continuous variables, central tendency and dispersion statistics were calculated. Chi-square or *t*-test was used for bivariate analysis, without correction for multiple testing.

### Ethics

Written approval for inclusion in this study was obtained in the first questionnaire. No ethics approval was sought, as this was a survey with healthcare professionals and not patients.

### Reporting summary

Further information on research design is available in the Nature Research Reporting Summary linked to this article.

## Results

The questionnaire was long (found in the Supplementary Materials). Hence, a selection of the results and questions are described in the publication and the rest are included in the Supplementary Material. Results showed in this publication are based on the response of all panellists, panellists’ results analysed by specialty and country are shown in Supplementary Information 1.

### Descriptive results

A total of 144 panellists completed the first round of the questionnaire and 127 panellists completed both rounds of the questionnaire (45 GPs and 82 pulmonologists and 0 internal medicine specialists). The average age of the panellists was 46.3 years and 48.0% stated they had more than 15 years of experience treating patients with COPD. We obtained a sample of panellists representing university hospitals, non-university hospitals, primary care centres and individual practices or offices with few health professionals.

On average, each panellist reported having seen 150 COPD patients (pulmonologists saw 3 times more patients than GPs, 205 versus 49) in the last 6 months: 21.5% patients were first consultations (78.5% follow up patients) and 54.0% were current smokers.

All panellists were familiar with the GOLD (A, B, C, D) classification for treatment initiation, and 89.8% of them applied it. Both pulmonologists and GPs considered the GOLD report the most important source to guide treatment decisions.

**Fig. 2:**
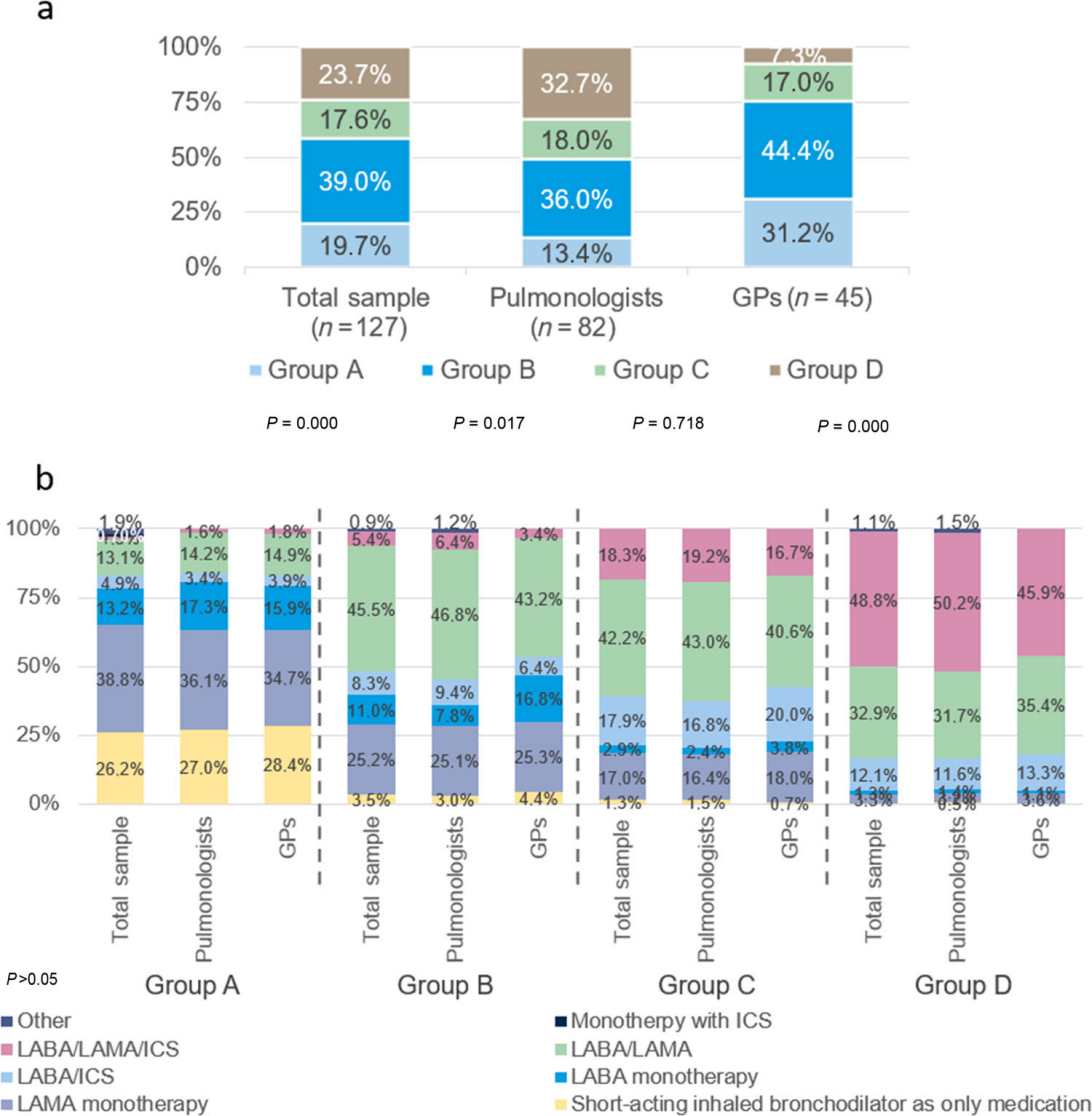
Distribution of COPD patients. Distribution of COPD patients according to the GOLD (A, B, C, D) classification (**a**) and the initial treatment prescribed (**b**). GP general practitioner, LABA long-acting beta-agonists, LAMA long-acting muscarinic antagonist, ICS inhaled corticosteroids. *p* values refer to the differences between specialties for each statement. Statistically significant differences were considered when *p* value < 0.05.

Figure 2 shows the distribution of patients seen by the panellists in the last 6 months according to GOLD (A, B, C, D) classification and the initial maintenance treatment per category for first visit patients only (23.3%).

Specialty did not influence the choice of initial treatment according to GOLD group. Overall, in group A (19.7%) patients, LAMA monotherapy was the main treatment initially prescribed (38.8% of the patients). While in group B (39.0%) and C (17.6%) patients, LABA/LAMA therapy (45.5% and 42.2% of the patients, respectively) was primarily chosen. In group D (23.7%) patients, the main initial treatment was triple therapy (48.8%). Statistically significant differences were observed when analysing prescriptions per country.

**Fig. 3:**
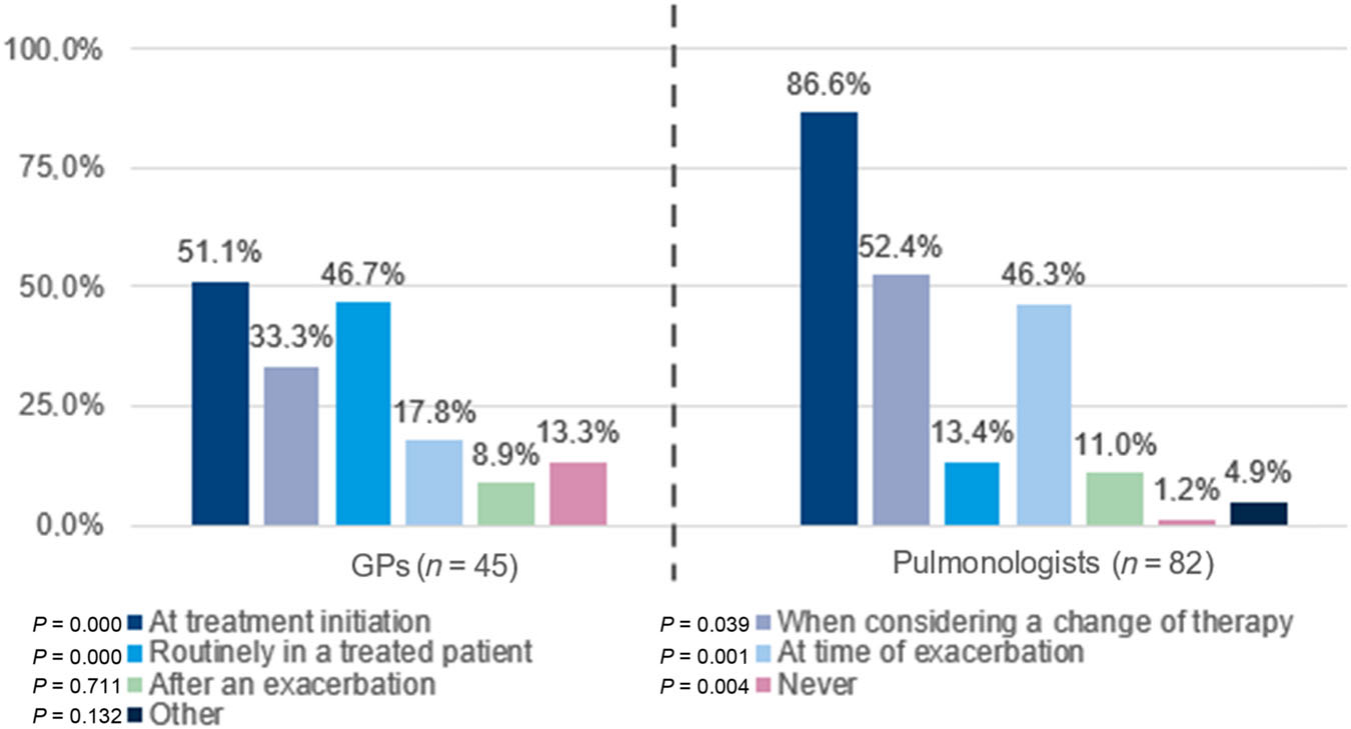
Use of blood eosinophil counts at different stages in COPD management in primary and secondary care. GPs general practitioners. *p* values refer to the differences between specialties for each statement. Statistically significant differences were considered when *p* value <0.05.

The most frequently used tests for stable COPD patients are (1) physical examination (every 3–6 months); (2) Clinical COPD Questionnaire (CCQ)/COPD Assessment Test (CAT) or Modified Medical Research Council (mMRC) dyspnoea Scale (every 6–12 months) and (3) blood eosinophil counts (once a year or less). GPs evaluate blood eosinophil counts for treatment initiation (51.1%) and routinely in treated patients in their clinical practice (46.7%). To a lesser extent, they use blood eosinophil counts when considering a change of therapy (33.3%) and as a biomarker for treatment decisions during exacerbations (17.8%). More pulmonologists used them for initial treatment decision (86.6%), when considering a change of therapy (52.4%), and as a biomarker for treatment decisions during exacerbations (46.3%). In the specialist care it was not commonly measured as a routine in treated patients (13.4%) (Fig. 3).

Both GPs (88.9%) and pulmonologists (96.3%) reported having ever withdrawn ICS treatment from a patient with COPD. Of their total ICS-treated COPD patients seen in the last year, over 22.0% were eligible for ICS withdrawal, and 17.2% were withdrawn from treatment. The main reason for withdrawal was ICS treatment no longer indicated (49.5%). Panellists reported a high percentage of successful withdrawals, without the need for ICS re-introduction and no exacerbations within 6 months of withdrawal in 75.0% of the patients. In total, 16.0% of the COPD patients needed re-introduction of ICS within 6 months of withdrawal (Table 1).

**Table 1:** Panellists experience on ICS withdrawal in COPD patients.

			Results by specialty		
		Total sample	General practitioners	Pulmonologists	*p* value
Patients’ distribution per panellist, in the last year	*N* respondents	127	45	82	
Total COPD patients seen in the last year, mean		300	97	410	<0.0001
ICS-treated patients, *N* (%)		117 (37.1%)	29 (28.2%)	165 (42.0%)	<0.0001
ICS-treated patients eligible for ICS withdrawal, %		22.0%	24.9%	20.4%	<0.0001
Have you ever withdrawn ICS treatment from any COPD patient? Yes, *n* (%)		119 (93.7)	40 (88.9)	79 (96.3)	0.098
Patients withdrawn from ICS treatment, %		17.2%	14.5%	18.5%	0.207
Patients that refused ICS withdrawal, %		8.6%	11.1%	7.4%	0.251
Patients distribution per panellist (only those who have withdrawn an ICS treatment in the last year)	*N* respondents	111	34	76	
Reason for ICS withdrawal, %					
No longer indication for ICS		49.5%	49.2%	49.6%	0.956
Lack of response to ICS		27.1%	27.4%	26.9%	0.935
Pneumonia		11.8%	12.2%	11.6%	0.880
Fear of adverse events to ICS		11.2%	11.2%	11.3%	0.979
Other		0.5%	0.0%	0.7%	0.256
Outcome of ICS withdrawal, %					
Remained with no exacerbations within 6 months after ICS withdrawal		75.0%	77.0%	74.0%	0.500
Needed ICS treatment reintroduction within 6 months after ICS withdrawal		16.0%	12.0%	18.0%	0.069
Reasons for ICS re-introduction (only among those who needed a re-introduction), %	*N* respondents	81	19	62	
Patient preference		16.0%	28.0%	12.0%	0.010
Increased symptoms		37.0%	35.0%	37.0%	0.762
Exacerbation		42.0%	37.0%	44.0%	0.387
Other		5.0%	0.0%	6.0%	0.166

*p* values refer to the differences between specialties for each statement. Statistically significant differences were considered when *p* value <0.05.

*ICS* inhaled corticosteroids, *COPD* chronic obstructive pulmonary disease.

Nearly half of panellists (48.8%) relied on exacerbation frequency and blood eosinophil counts, while 35.4% relied on the frequency of exacerbations only for decision making around ICS withdrawal. Most pulmonologists (70.7%) reported that ICS withdrawal should be abrupt in patients on LABA/ICS therapy followed by LAMA/LABA introduction, while most of the GPs (68.4%) preferred gradual dose reduction and introduction of LAMA/LABA treatment at the same time. In case of patients on triple therapy, 74.0% of panellists (89.0% pulmonologist versus 46.7% GPs) agreed that ICS should be withdrawn by an abrupt dose reduction while maintaining LAMA/LABA combination (Table 2).

**Table 2:** Panellist’s opinion on how ICS therapy should be withdrawn from their COPD patients.

		Results by specialty	
	Total sample (*N* = 127)	General practitioners (*N* = 45)	Pulmonologists (*N* = 82)
In your decision to withdraw ICS therapy, which parameter do you rely on most? (Multiple choice option)			
Frequency of exacerbations	35.4%	42.2%	31.7%
Level of blood eosinophil counts	1.6%	2.2%	1.2%
Always both in combination	48.8%	44.4%	51.2%
None of these two	3.9%	0.0%	6.1%
Other factors	10.2%	11.1%	9.8%
For what reasons do you think ICS-treated patients can be eligible for ICS withdrawal? (Multiple choice option)			
Patients with pneumonia	68.5%	73.3%	65.9%
No longer indication for ICS	87.4%	93.3%	84.1%
Lack of response to ICS	88.2%	86.7%	89.0%
Patients without asthma	39.4%	46.7%	35.4%
Patients with diabetes	10.2%	11.1%	9.8%
Patients with osteoporosis/osteopenia	17.3%	17.8%	17.1%
Patients with cardiovascular disease	1.6%	4.4%	0.0%
Patients with ICS-related side effects	81.1%	86.7%	78.0%
Patients should never be withdrawn from ICS treatment	0.0%	0.0%	0.0%
How confident you are in the following situations:			
Withdrawal in case of no longer indication for ICS. Confident, %	87.4%	91.1%	85.4%
ICS withdrawal in case of lack of response to ICS. Confident, %	77.2%	82.2%	74.4%
ICS withdrawal in case of pneumonia. Confident^a^	63.0%	77.8%	54.9%
In your personal opinion, how should ICS be withdrawn in patients on LABA/ICS therapy? (Multiple choice option)			
Gradual ICS dose reduction without adding any other treatment in all patients	0.8%	0.0%	1.2%
Gradual ICS dose reduction without adding any other treatment in patients on high dose ICS	3.9%	0.0%	6.1%
Abrupt ICS withdrawal without adding any other treatment	7.9%	6.7%	8.5%
Gradual dose reduction and LAMA/LABA treatment introduction at the same time^a^	55.1%	68.9%	47.6%
Gradual dose reduction and LAMA/LABA treatment once ICS is completely withdrawn	0.8%	2.2%	0.0%
Abrupt ICS withdrawal and LAMA/LABA treatment once ICS is completely withdrawn	59.1%	37.8%	70.7%
ICS should not be withdrawn from patients on LABA/ICS	0.0%	2.2%	1.2%
Other	1.6%	0.0%	3.2%
In your personal opinion, how should ICS be withdrawn in patients on triple therapy (LAMA/LABA/ICS)? (Multiple choice option)			
Gradual ICS dose reduction maintaining LAMA/LABA combination^a^	28.3%	55.6%	13.4%
Abrupt ICS withdrawal maintaining LAMA/LABA combination^a^	74.0%	46.7%	89.0%
ICS should not be withdrawn from patients on LABA/ICS	0.0%	0.0%	0.0%
Other	0.8%	2.2%	0.0%
In your personal opinion, what drives the choice of LAMA/LABA combination when withdrawing ICS in patients who were on triple therapy? (Multiple choice option)			
Same device^a^	89.8%	97.8%	85.4%
Same LAMA	6.3%	8.9%	4.9%
Same LABA	6.3%	8.9%	4.9%
Switch to a potentially more effective LAMA	9.4%	6.7%	11.0%
Switch to a potentially more effective LABA	5.5%	2.2%	7.3%
Other	2.4%	0.0%	3.7%
In your personal opinion, after ICS withdrawal, how should patients with COPD be monitored? (Multiple choice option)			
No specific follow-up	0.0%	0.0%	0.0%
Planned follow-up visit/call 1 month after ICS withdrawal^a^	73.2%	86.7%	65.9%
Planned visit for spirometry	33.9%	24.4%	39.0%
Patients should have the possibility to communicate with me or another healthcare professional in case of questions	56.7%	73.3%	47.6%
Patients should have the possibility to call me or another healthcare professional in case of complaints	32.3%	42.2%	26.8%
Other	0.8%	0.0%	1.2%
Please indicate the reasons that could indicate the need for reintroducing ICS (Multiple choice option)			
Worsening of COPD symptoms (such as breathlessness)	68.5%	75.6%	64.6%
Exacerbations after ICS withdrawal^a^	95.3%	88.9%	98.8%
Persistent adverse events after ICS withdrawal^a^	5.5%	11.1%	2.4%
Significant worsening of spirometry	66.1%	62.2%	68.3%
Patient preference	7.9%	6.7%	8.5%
Others	0.0%	0.0%	0.0%
What should be done in such cases? (Multiple choice option)			
Back to the ICS treatment at the same doses than before	35.4%	22.2%	42.7%
Back to the ICS treatment at lower doses than before	63.8%	75.6%	57.3%
Keep the ICS withdrawal	0.8%	2.2%	0.0%
Other	0.0%	0.0%	0.0%

*LABA* long-acting beta-agonists, *LAMA* long-acting muscarinic antagonist, *ICS* inhaled corticosteroids, *COPD* chronic obstructive pulmonary disease.

^a^Statistical difference between GP and pulmonologist.

The results showing statistically significant difference between specialties are described as follows: (1) How confident you are with ICS withdrawal in case of pneumonia (*p* value 0.023); (2) ICS in patients on LABA/ICS therapy should be withdrawn by gradual dose reduction and LAMA/LABA treatment introduction at the same time (*p* value 0.021); (3) ICS in patients on triple therapy should be withdrawn by gradual ICS dose reduction maintaining LAMA/LABA combination (*p* value < 0.0001) and (4) with abrupt ICS withdrawal maintaining LAMA/LABA combination (*p* value < 0.0001); (5) The choice of LAMA/LABA combination when withdrawing ICS in patients who were on triple therapy is driven by the same device (*p* value 0.027); (6) After ICS withdrawal, patients with COPD should be monitored with planned follow-up visit and a call 1 month after ICS withdrawal (*p* value 0.011); (7) The reasons that could indicate the need for reintroducing ICS is exacerbations after ICS withdrawal (*p* value 0.012) and (8) persistent adverse events after ICS withdrawal (*p* value 0.041).

### Discrete choice experiment results

The relationship between different criteria and values combined creating patient profiles and the initial COPD treatment choice was assessed using a discrete choice experiment (see details in Fig. 1). Smoking status was not statistically significant in the model, and therefore not included. Details on the results found are presented in Table 3.

**Table 3:** Multinomial logistic regression model showing the variables associated with the initial treatment decision.

Criteria	Variables	LAMA monotherapy	LABA/LAMA	LABA/ICS	TT (LAMA/LABA/ICS)
		OR (CI 95%, *p* value)	OR (CI 95%, *p* value)	OR (CI 95%, *p* value)	OR (CI 95%, *p* value)
Lung function	FEV < 50%	0.634 (0.32–1.25, *p* = 0.19)	1.001 (0.52–1.92, *p* = 0.999)	0.75 (0.38–1.49, *p* = 0.409)	1.574 (0.8–3.09, *p* = 0.188)
	FEV ≥ 50%^a^				
Symptoms	Severe	0.307 (0.12–0.79, *p* = **0.014**)	2.632 (1.09–6.34, *p* = **0.031**)	1.221 (0.49–3.05, *p* = 0.669)	9.55 (3.98–24.88, *p* < **0.0001**)
	Moderate	0.489 (0.23–1.05, *p* = 0.067)	1.56 (0.75–3.26, *p* = 0.237)	0.686 (0.32–1.49, *p* = 0.34)	2.623 (1.19–5.78, *p* = **0.017**)
	Mild^a^				
Number of exacerbations in the last year	≥1 with hospitalisation	0.201 (0.08–048, *p* < **0.0001**)	0.857 (0.38–1.94, *p* = 0.711)	2.564 (1.09–6.04, *p* = **0.031**)	7.733 (3.26–18.34, *p* < **0.0001**)
	≥1 without hospitalisation	0.445 (0.19–1.02, *p* = 0.056)	0.941 (0.42–2.1, *p* = 0.881)	1.637 (0.7–3.82, *p* = 0.254)	4.126 (1.75–9.7, *p* = **0.001**)
	None^a^				
Blood eosinophil count	<100 eos/μl	0.816 (0.31–2.14, *p* = 0.679)	0.873 (0.34–2.21, *p* = 0.774)	0.516 (0.18–1.5, *p* = 0.224)	0.499 (0.19–1.34, *p* = 0.168)
	100–300 eos/μl	1.344 (0.38–4.69, *p* = 0.643)	1.6 (0.47–5.43, *p* = 0.451)	3.017 (0.83–10.98, *p* = 0.094)	3.085 (0.88–10.8, *p* = 0.078)
	≥300 eos/μl	0.185 (0.07–0.48, *p* = **0.001**)	0.18 (0.07–0.45, *p* < **0.0001**)	2.999 (1.15–7.8, *p* = **0.024**)	1.468 (0.58–3.72, *p* = 0.418)
	Unknown blood eosinophil count^a^				

Only significant results (*p* < 0.05) are in bold.

*CI* confidence interval, *FEV* forced expiratory volume, *LABA* long-acting beta-agonists, *LAMA* long-acting muscarinic antagonist, *ICS* inhaled corticosteroids, *TT* triple therapy, *eos* eosinophiles.

^a^Reference category.

Results of the multivariate analysis indicate that severe symptoms, 1 or more exacerbations leading to hospitalisation and blood eosinophil counts ≥300 cells/µl were associated with not initiating treatment with LAMA monotherapy. A patient profile with severe symptoms leads to the initiation with LAMA/LABA therapy. In contrast, blood eosinophil counts ≥300 cells/µl results in the decision to not initiating treatment with LAMA/LABA. The presence of 1 or more exacerbations leading to hospitalisation and blood eosinophil counts ≥300 cells/µl are drivers for panellists to prescribe LABA/ICS therapy. Meanwhile, the presence of moderate to severe symptoms and 1 or more exacerbations leading to hospitalisation influence panellists to prescribe triple therapy. The relative weight of lung function values did not reach significance in the multinomial model, although it was significant in the univariate analysis (results shown in Supplementary Table 7).

### eDELPHI results

**Fig. 4:**
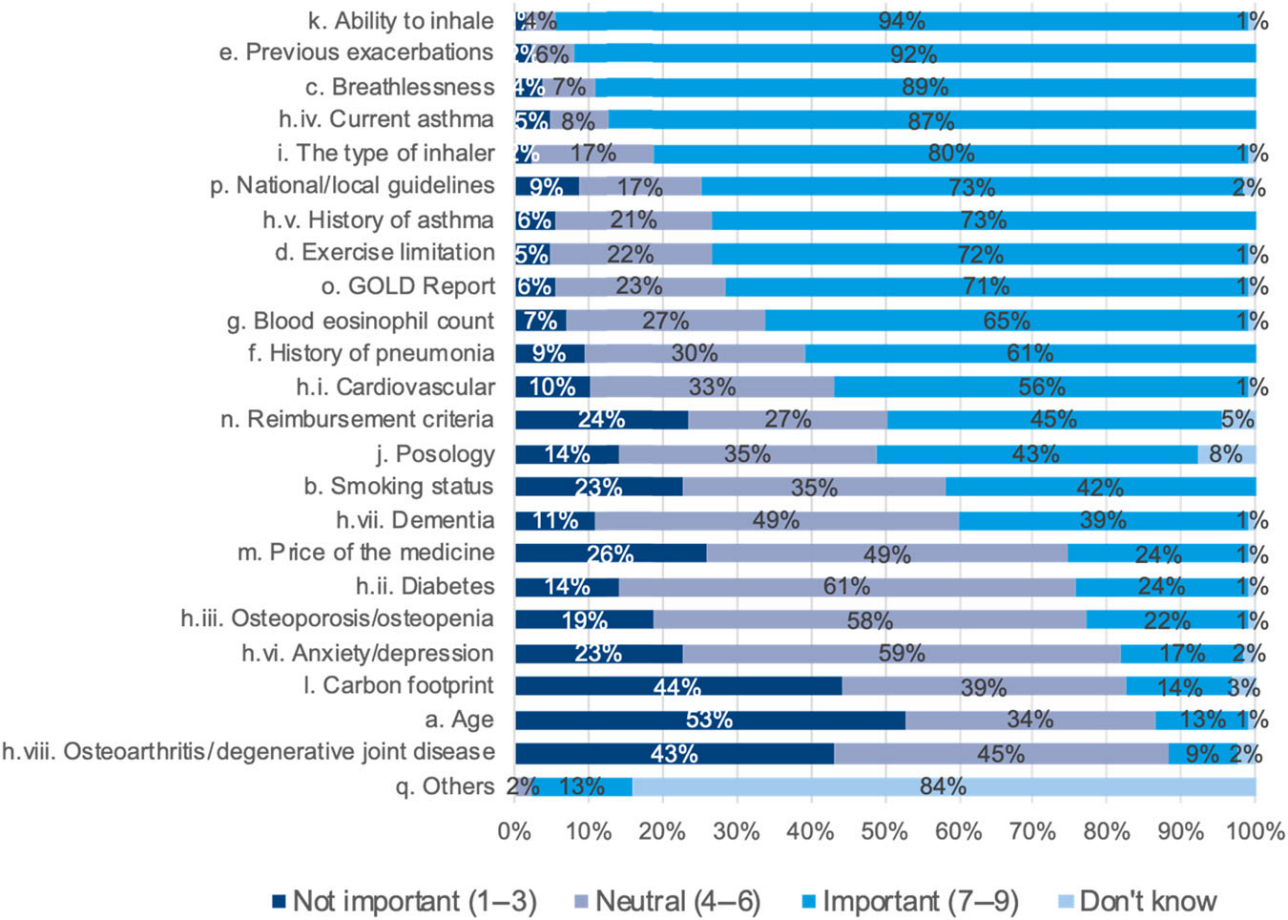
Importance of each criterion for the selection of initial COPD treatment.

Panellists were asked about the importance they attributed to each criterion for the selection of the initial COPD treatment (Fig. 4). The patient’s ability to inhale (93.7% of agreement), previous exacerbations (92.1%), breathlessness (89.0%), and the type of inhaler (80.3%) were found the most important aspects considered by panellists for the selection of an initial COPD treatment. Comorbidities, smoking status, price or level of reimbursement was considered important by much fewer panellists for COPD treatment decision. Nearly two-third (65.4%) of panellists (57.8% of GPs and 69.5% of pulmonologists) considered blood eosinophil counts as important, although this did not reach the pre-defined consensus threshold of 70% (Fig. 4).

Most panellists (84.3%) agreed that LAMA/LABA improve breathlessness compared to LAMA and LABA/ICS. LAMA/LABA was also considered to improve quality of life (79.5%) and physical activity (73.3%) compared to LAMA, but no consensus (pre-defined as 70% agreement) was reached on this when compared to LABA/ICS. Similarly, most panellists (85.0%) agreed that LAMA/LABA can be the initial treatment for some patients, and there was no consensus that LAMA should be the initial COPD treatment before prescribing LAMA/LABA. There was a clear disagreement (78.0%) with the statement in the questionnaire if LABA/ICS should be prescribed as initial treatment before LAMA/LABA. No significant differences were found between specialities regarding the above.

Panellists believed that LABA/ICS treatment may decrease exacerbations to a greater extent than LAMA/LABA in patients with a high exacerbation risk and high eosinophil blood counts, but not in case of low exacerbation risk and low blood eosinophil counts. When considering prescribing ICS, panellists generally considered blood eosinophil counts, exacerbation risk, and comorbidities (70.1% agreement), and generally took into account any uncertainty of concomitant asthma diagnosis (77.1%). There was consensus regarding the importance of considering comorbidities when selecting COPD treatment (78.7%) and that long-term ICS prescriptions should be re-assessed to verify their efficacy (73.2%). Panellists did not agree on the potential risks of ICS to increase bruising or individual comorbid diseases such as tuberculosis, diabetes and osteoporosis. Statements such as ICS being less beneficial in smokers or that treatment with ICS increases the risk of pneumonia (69.3% agreed) did not reach consensus among panellists. No significant differences were found between specialities regarding the risks and benefits of ICS treatment in COPD patients.

The majority of panellists (79.5%) agreed that ICS is over-prescribed in the primary care setting and felt confident to withdraw ICS when no longer indicated (87.4%) or due to a lack of response (77.2%). Both specialties agreed that GPs generally felt insecure about ICS withdrawal (70.9%).

Panellists believed that increasing the use of LAMA/LABA in patients for whom LABA/ICS is not indicated could increase clinical response to treatment (82.7% of agreement), better COPD control (82.7%), and a reduction in exacerbations (70.1%). In patients with further exacerbations with high eosinophil count, stepping up the inhaled treatment from LAMA/LABA to triple therapy may result in better COPD control (84.3%), prevent exacerbations (83.5%), and increase clinical response to treatment (81.9%). Panellists also believed that reducing the use of non-indicated ICS in COPD patients could result in a reduction of bacterial exacerbations (78.0%), a better outcome of co-existing diseases (76.4%), and a reduction of adverse events such as infections (76.4%). No significant differences were found between specialities.

Consensus was reached regarding the need for resources to optimise management and treatment of COPD patients, such as: tobacco cessation programmes (85.0% of agreement), recommendations for treatment management of multi-morbid patients (81.9%), access to a validated tool that helps to identify patients that would benefit from ICS (80.3%), among others. In terms of the economic impact of COPD treatment, the panellists agreed that exacerbations (92.9%) and pneumonia (85.0%) represent a major financial burden for healthcare expenditures. In general, no significant differences were found between specialities regarding the clinical impact of treatment optimisation.

## Discussion

This study aimed to understand current practice, attitudes and rationales in COPD management in both primary and secondary care in six European countries. The study results were in line with other real-world cohorts^18–22^.

The eDELPHI study demonstrated that the most important criteria to select COPD initial treatment were the ability to inhale, breathlessness, as well as the history of exacerbation and concomitant asthma. These are deemed more relevant than other criteria such as blood eosinophils, comorbidities and the GOLD classification.

All panellists were familiar with the GOLD classification and the vast majority reported to use it for treatment initiation. A significant proportion of the panellists of our study declared to start maintenance therapy with a LAMA/LABA. This is in accordance with another DELPHI study that showed the central place of LAMA/LABA in the treatment of COPD patients^23^, and in line with the Cochrane review showing the benefits of dual bronchodilation over mono bronchodilation^24^. The COPD guidelines of the American Thoracic Society recommend LAMA/LABA as initial treatment for symptomatic patients and patients with exercise limitation^25^. However, the high percentage of patients being prescribed an ICS-based regimen as initial maintenance treatment, even by pulmonologists, shows a mismatch between intention to adhere to GOLD and GOLD adherence in clinical practice. Overuse of ICS is already well documented in real-world studies^26,27^. The newly commercialised triple therapies are being frequently used as initial maintenance therapy^28^, even in less severe COPD patients, such as in GOLD A and B group patients^28,29^.

The use of eosinophil counts as biomarker has been proposed by GOLD to maximise benefit and minimise harm of COPD treatment choices^30,31^. According to the GOLD guidance, the threshold for ICS initiation used in patients with exacerbations is 300 cell/µl, and 100 cell/µl for patients on LAMA/LABA who still experience exacerbations. Our study showed no consensus on the importance of use of blood eosinophil counts for stable COPD patients. A difference of its use between GPs and pulmonologists was reported. While general practitioners use blood eosinophil counts routinely in a treated patient, pulmonologists commonly use it as biomarkers for individualised treatment decisions, for initiation and during exacerbations. In our discrete choice experiment, high eosinophil counts were associated with treatment initiation with LABA/ICS and with triple therapy in the univariate analysis, but interestingly not with triple therapy in the multivariate analysis. Overall, the results of the study suggest the need to increase awareness of the option to use blood eosinophils as a biomarker for treatment choice, especially in primary care.

Our study shows that panellist agree to consider comorbidities when selecting COPD treatment. However, the lack of consensus on the potential risks of ICS containing treatment and the need for re-assessment regarding each comorbidity suggests overconfidence among panellists regarding LABA/ICS safety in COPD patients. Clinical trials demonstrate that high doses of ICS increase the number of pneumonias in COPD patients^32^, while other observational studies describe long-term consequences of ICS in patients with diabetes or osteoporosis, especially when used with high dose^33^. Hence, personalised questions can aid physicians during consultation time to find the best treatment option for the individual patient with the use of tools such as International Primary Care Respiratory Group (IPCRG) desktop helpers^10,34^.

Panellists were well aware of the factors that may indicate if a patient should be withdrawn from ICS, and they relied mostly on exacerbation frequency and the level of blood eosinophils^35^. However, in our study, GPs felt less confident than pulmonologists when it comes to ICS withdrawal. When a patient had no exacerbation history and low eosinophil counts, panellists were confident on their decision. However, in case of high exacerbation frequency with low eosinophil counts or low exacerbation frequency with high eosinophil counts, they felt less confident. In case of frequent exacerbations but low blood eosinophil counts, ICS may not be effective as the type of inflammation is not eosinophilic. Higher blood eosinophil counts are associated with increased eosinophilic lung inflammation, where ICS treatment shows an adequate response. However, emerging data indicates that lower blood eosinophil counts are associated with an increased risk of bacterial infection, suggesting complex relationships between eosinophils, ICS response, and the airway microbiome^36^. In fact, the effects of triple therapy on exacerbation are dependent on blood eosinophil counts as well as smoking status^37^, while infrequent exacerbators do not experience increased risk of exacerbation upon discontinuation of ICS from triple therapy^38^. Different approaches have been proposed for ICS withdrawal either abruptly or gradually. The European Respiratory Society has recently published a guideline on withdrawal of inhaled corticosteroids in COPD^39^. This defines the eligible patients for withdrawal and lists further documents proposing guidance on how to do it, such as IPCRG desktop helper^40^. There are also national withdrawal guidance documents^41–43^. In our study, a significant proportion of panellists showed experience in ICS withdrawal with great success and no need for ICS re-introduction. Abrupt ICS withdrawal and introduction of LAMA/LABA treatment was the preferred choice for pulmonologists, whereas GPs prefer to reduce the dose of ICS before switching to a LAMA/LABA.

Regarding the economic impact of COPD treatment, the panellists agreed that COPD exacerbation and pneumonia represent a financial burden for healthcare providers that can be reduced by proper use of COPD treatments^44^. Several other studies have modelled the economic impact of treatment optimisation. In short, withdrawing ICS in patients when indicated and increasing LAMA/LABA would lead to fewer exacerbations, fewer pneumonia, and fewer diabetes-related events, and, therefore, a significant cost reduction for the healthcare systems^17,44,45^. A more recent systematic study described the trend of healthcare cost for European countries studies (higher costs for severe COPD and frequent history of exacerbations). The main costs exposed were hospitalisation, pharmacological treatment, loss of productivity ad premature retirement, all of which can be reduced with strong healthcare systems that prioritise monitoring, evaluation and health education^46^.

Our survey showed a gap between best practice and behaviour, which indicates further efforts are needed to improve the adherence to guidelines in clinical practice. This could be carried out by increasing the physicians’ time and tools to assess factors indicating treatment initiation or optimisation. It also highlights the need to amend behaviour and provide better education on how exacerbations in COPD can be avoided. This in turn leads to the importance of individualised management through discussions and personalised questions to address the real need and offer the best treatment option for the individual patient^37,38^.

In conclusion, the study identified a mismatch between intention-to and actual GOLD guideline adherence, reflected in ICS over-prescription. However, in our study, panellists agreed with the importance of continuous re-evaluation of treatment decisions. Initial maintenance treatment choice is highly influenced by the ability to inhale. The drivers for selecting LAMA/LABA as initial treatment were severe symptoms without high blood eosinophil counts. High blood eosinophil counts were found as drivers for LABA/ICS initiation, as recommended for GOLD D patients. While not recommended as initial therapy, drivers for LAMA/LABA/ICS prescriptions were found to be severe symptoms and exacerbation history with or without hospitalisation. With regards to ICS de-escalation in the eligible patients, pulmonologists were more confident to de-escalate ICS treatment in eligible patients than GPs.

## Strengths and limitations

The comprehensive nature, Delphi methodology and multi-country coverage result in a robust conclusion around COPD treating physicians’ current perceptions and opinions.

A key limitation of the study is the sampling method, differing per country (e.g., database list, peer-to-peer invitation). In order to avoid selection bias, a random selection of panellists from databases was operated, except for panellists invited by a peer. Furthermore, the length of the eDELPHI questionnaire could have led to panellist fatigue and selection bias. This was minimised by performing a pilot study among 4 GPs and 4 pulmonologists. Sample size limitations may have led to insufficient evidence for detecting differences between countries. In the case of specialties, the larger numbers of pulmonologist may have led to overrepresentation of this specialty. In addition, GPs and pulmonologists were asked to fill out the survey during the COVID-19 pandemic, which may have led to different perceptions, fewer patients or less observed exacerbations compared to a pre-pandemic situation. Finally, social desirability bias may have impacted the results. Nevertheless, the mismatch between described behaviours and the knowledge of the guidelines does suggest that not only socially desired answers were given.

## Supplementary information

Supplementary information 1

Reporting Summary

Appendix 1

## Data Availability

The authors declare that the data supporting the findings of this study are available within the paper and its Supplementary Information files. On request, further information can be obtained via the corresponding author.
