# Supplementary material for: Investigating the rationale for COPD maintenance therapy prescription across Europe, findings from a multi-country study
Source: NPJ Prim Care Respir Med. 2023 May 3;33:18. doi: 10.1038/s41533-023-00334-x (PMC10154184; doi:10.1038/s41533-023-00334-x)
Supplement: Supplementary file 1 — Supplementary information 1 [file 41533_2023_334_MOESM1_ESM.pdf]

Supplementary information

Supplementary Table 1. Study participants and patient's profiles 2
Supplementary Table 2. Importance of the sources for treatment decisions. Global results and sub-analysis by medical speciality and by country 4
Supplementary Table 3. Use of GOLD for treatment initiation and follow-up of COPD treatments. Global results and sub-analysis by medical speciality, by country and by age of the participants. 5
Supplementary Table 4. Distribution of COPD patients according to the GOLD (A, B, C, D) classification and the initial treatment prescribed. Global results and sub-analysis by medical speciality, by country and by age of the participants. 6
Supplementary Table 5. Clinicians' opinion on the use of fixed dose LAMA/LABA in COPD treatment. Global results and sub-analysis by medical speciality. 9
Supplementary Table 6. Clinicians' opinion on the use of fixed dose LAMA/LABA in COPD treatment. Global results and sub-analysis by country. 11
Supplementary Table 7. Clinicians' opinion on the risks and benefits of ICS treatment in COPD patients. Global results and sub-analysis by medical speciality. 14
Supplementary Table 8. Clinicians' opinion on the risks and benefits of ICS treatment in COPD patients. Global results and sub-analysis by country. 18
Supplementary Table 9. Importance of the criteria for the selection of an initial COPD treatment. Global results and sub-analysis by medical speciality. 23
Supplementary Table 10. Importance of the criteria for the selection of an initial COPD treatment. Global results and sub-analysis by country. 26
Supplementary Table 11. Multinomial logistic regression model showing the variables associated with the initial treatment decision. 31
Supplementary Table 12. Relevant criteria for the initial treatment decision - Univariate analysis. 31
Supplementary Table 13. Opinion on the current use of inhaled corticosteroids. Global results and sub-analysis by medical specialty. 33
Supplementary Table 14. Opinion on the current use of inhaled corticosteroids. Global results and sub-analysis by country. 35
Supplementary Table 15. Participants experience on ICS withdrawal in COPD patients. Global results and sub-analysis by country. 39
Supplementary Table 16. Participant's opinion on how ICS therapy should be withdrawn from their COPD patients. Global results and sub-analysis by specialty. 40
Supplementary Table 17. Participant's opinion on how ICS therapy should be withdrawn from their COPD patients. Global results and sub-analysis by country. 42
Supplementary Table 18. Potential impact of COPD treatment optimization. Global results and sub-analysis by medical speciality. 46
Supplementary Table 19. Potential impact of COPD treatment optimization. Global results and sub-analysis by country. 49
Supplementary Table 20. Resources to optimize the COPD patient management and treatment. Global results and sub-analysis by medical speciality. 52
Supplementary Table 21. Resources to optimize the COPD patient management and treatment. Global results and sub-analysis by country. 55
Supplementary Table 22. Economic impact of COPD treatment. Global results and sub-analysis by medical speciality. 59
Supplementary Table 23. Economic impact of COPD treatment. Global results and sub-analysis by country. 60
Supplementary Table 24. Inhaler selection: importance of patient and device characteristics. Global results and sub-analysis by medical speciality. 62
Supplementary Table 25. Inhaler selection: importance of patient and device characteristics. Global results and sub-analysis by country. 65

Supplementary Table 26. Participant's knowledge and opinions on COPD devices characteristics. Global results and sub-analysis by medical speciality and by country. 69
Supplementary Table 27. Clinicians' opinion on the potential impact of COPD treatment optimization. Global results and sub-analysis by medical speciality. 72
Supplementary Table 28. Clinicians' opinion on the potential impact of COPD treatment optimization. Global results and sub-analysis by country. 75
Supplementary Table 29. Important aspects for treatment decisions. Global results and sub-analysis by medical speciality and by country. 79

Supplementary Table 1. Study participants and patient's profiles

		**Results by speciality**		
	**Total sample**	**Pulmonologists**	**GPs**	**p value**
Medical specialty, n (%)	127 (100%)	82 (64.6%)	45 (35.4%)	
Country, n (%)				0.006
Belgium	26 (20.5%)	18 (22.0%)	8 (17.8%)	
Finland	7 (5.5%)	6 (7.3%)	1 (2.2%)	
Greece	17 (13.4%)	17 (20.7%)	0 (0%)	
Norway	25 (19.7%)	14 (17.1%)	11 (24.4%)	
The Netherlands	18 (14.2%)	11 (13.4%)	7 (15.6%)	
Portugal	34 (26.8%)	16 (19.5%)	18 (40.0%)	
Type of centre, n (%)				0.000
University hospital	18 (14.2%)	18 (22.0%)	0 (0%)	
Non-university hospital	49 (38.6%)	49 (59.8%)	0 (0%)	
Primary care centre (≥ 5 physicians)	33 (26.0%)	5 (6.1%)	28 (62.2%)	
Individual office/office with few healthcare professionals	36 (28.3%)	16 (19.5%)	20 (44.4%)	
Work setting, n (%)				0.383
Public centre	74 (58.3%)	48 (58.5%)	26 (57.3%)	
Private centre	24 (18.9%)	13 (15.9%)	11 (24.4%)	
Both	29 (22.8%)	21 (25.6%)	8 (17.8%)	
Age, Mean ± SD	46.3 ± 10.2	45.5 ± 11.1	46.8 ± 9.7	0.497
Years of experience, n (%)				0.324
3-5 years	13 (10.2%)	8 (9.8%)	5 (11.1%)	
5-10 years	29 (22.8%)	15 (18.3%)	14 (31.1%)	
10-15 years	24 (18.9%)	18 (22.0%)	6 (13.3%)	
>15 years	61 (48.0%)	41 (50.0%)	20 (44.4%)	
COPD patients seen				
Total COPD patients, mean	150	205	49	0.000
1st visit patients, %	21.5%	25.1%	14.9%	0.000
Follow-up patients, %	78.5%	74.9%	85.1%	0.000
Current smoker patients, %	54%	54.1%	53.7%	0.939

Comorbidities of their COPD patients, %				
Pulmonary pathology				
Asthma	15.3%	14.9%	16.1%	0.436
Chronic bronchitis	46.1%	46.4%	45.6%	0.875
Pulmonary hypertension	12.3%	15.2%	6.9%	0.000
Mental health				
Anxiety / depression	32.4%	34.1%	29.4%	0.213
Dementia	6.7%	6.7%	6.7%	0.977
Metabolic disease				
Dyslipidaemia	39.2%	37.2%	42.8%	0.187
Diabetes	35.7%	34.5%	37.9%	0.517
Obesity	20.5%	20.1%	21.4%	0.324
Osteoporosis	21.9%	22.9%	20.0%	0.360
Cardiovascular				
Hypertension	49.4%	49.0%	50.2%	0.757
Coronary heart disease	31.2%	34.4%	25.4%	0.003
Heart failure	23.1%	25.8%	18.2%	0.009
Other comorbidities				
Renal comorbidities	12.6%	11.9%	13.8%	0.301
Gastroenterological comorbidities	21.7%	21.4%	22.4%	0.733
Osteoarthritis, degenerative joint disease	25.1%	20.5%	33.6%	0.000
No comorbidities	9.0%	10.8%	5.6%	0.002

Supplementary Table 2. Importance of the sources for treatment decisions. Global results and sub-analysis by medical speciality and by country

**Sources for treatment decisions** *(items are displayed in order of importance)*	Total sample (N=127)	Results by speciality			Results by country						
		GPs (N=45)	Pulmonologists (N=82)	p value	Belgium (N=26)	Finland (N=7)	Greece (N=17)	Norway (N=25)	Portugal (N=34)	The Netherlands (N=18)	p value
	**Average score**	**Average score**			Average score						
	**Mean (SD)**	**Mean (SD)**			Mean (SD)						
GOLD Report (Global initiative for Chronic Obstructive Lung Disease)	2.6 (1.6)	2.8 (1.6)	2.5 (1.6)	p=0.527	2.6 (1.8)	2.4 (1.0)	2.8 (1.7)	2.5 (1.3)	2.4 (1.8)	2.8 (1.4)	0.964
My own experience	2.7 (1.0)	2.8 (1.0)	2.7 (1.1)	p=0.866	2.5 (0.9)	3.3 (0.8)	2.7 (1.2)	2.4 (1.2)	3 (0.8)	2.9 (1.1)	0.156
A national guideline	2.7 (1.2)	2.8 (1.4)	2.6 (1.0)	p=**0.014**	3.1 (0.8)	1.6 (1.5)	2.6 (0.9)	2.5 (1.4)	2.9 (1.0)	2.4 (1.3)	**0.027**
A local guideline	3.4 (1.4)	3.2 (1.6)	3.6 (1.3)	p=**0.029**	3.4 (1.6)	3.6 (1.3)	3.1 (1.2)	3.8 (1.4)	3.3 (1.5)	3.3 (1.5)	0.72
My colleagues advise	3.6 (1.5)	3.4 (1.4)	3.6 (1.5)	p=0.203	3.3 (1.5)	4.1 (1.2)	3.7 (1.8)	3.8 (1.1)	3.4 (1.5)	3.5 (1.7)	0.764

For the interpretation of the Average Score, sources were defined by 1 "The most important source " and 7 "the least important source".

Supplementary Table 3. Use of GOLD for treatment initiation and follow-up of COPD treatments. Global results and sub-analysis by medical speciality, by country and by age of the participants.

		Results by speciality			Results by country							Results by age of the participants		
	Total sample (N=127)	GPs (N=45)	Pulmonologists (N=82)	p value	Belgium (N=26)	Finland (N=7)	Greece (N=17)	Norway (N=25)	Portugal (N=34)	The Netherlands (N=18)	p value	<40 years old (N=38)	≥40 years old (N=89)	p value
**Are you familiar with the GOLD (A, B, C, D) classification for initial therapy?**				-							-			-
Yes	100.0%	100.0%	100.0%		100.0%	100.0%	100.0%	100.0%	100.0%	100.0%		100.0%	100.0%	
No	0.0%	0.0%	0.0%		0.0%	0.0%	0.0%	0.0%	0.0%	0.0%		0.0%	0.0%	
**Do you usually use it?**				0.71							**0.008**			0.227
Yes	89.8%	91.0%	89.0%		100.0%	71.4%	94.1%	80.0%	100.0%	72.2%		94.7%	87.6%	
No	10.2%	9.0%	11.0%			28.6%	5.9%	20.0%		27.8%		5.3%	12.4%	
**Are you familiar with the 2020 GOLD follow-up pharmacological treatment algorithm for COPD?**				**0.001**							**0.011**			0.418
Yes	82.7%	89.0%	71.0%		88.5%	85.7%	88.2%	64.0%	100.0%	61.1%		86.8%	80.9%	
No	17.3%	11.0%	29.0%		11.5%	14.3%	11.8%	36.0%		38.9%		13.2%	19.1%	
**Do you decide on follow-up treatment based upon the suggested treatable traits?**				0.257							0.225			0.533
Yes	49.5%	53.4%	40.6%		52.2%	16.7%	46.7%	31.3%	58.8%	63.6%		54.5%	47.2%	
No	1.9%	2.7%			4.3%					9.1%			2.8%	
Sometimes	48.6%	43.8%	59.4%		43.5%	83.3%	53.3%	68.8%	41.2%	27.3%		45.5%	50.0%	

Supplementary Table 4. Distribution of COPD patients according to the GOLD (A, B, C, D) classification and the initial treatment prescribed. Global results and sub-analysis by medical speciality, by country and by age of the participants.

			By speciality			By country							By age		
GOLD Group	Initial treatment prescribed	Total sample (N=127)	GPs (N=45)	Pulmonologists (N=82)	p value	Belgium (N=26)	Finland (N=7)	Greece (N=17)	Norway (N=25)	Portugal (N=34)	The Netherlands (N=18)	P value	< 40 years old (N=38)	≥ 40 years old (N=89)	p value
**Group A**	Short-acting inhaled bronchodilator as only medication	26.0%	27.0%	28.4%	0.008	**45.0%**	26.0%	10.0%	**39.0%**	15.0%	19.0%	**0**	26.2%	26.3%	**0.990**
	LAMA monotherapy	**39.0%**	36.1%	34.7%	0.504	25.0%	**37.0%**	**49.0%**	24.0%	**52.0%**	**43.0%**	**0.002**	43.3%	36.8%	**0.304**
	LABA monotherapy	13.0%	17.3%	15.9%	0.043	17.0%	8.0%	14.0%	10.0%	14.0%	12.0%	0.642	16.1%	11.9%	**0.194**
	LABA/ICS	5.0%	3.4%	3.9%	0.139	5.0%	3.0%	7.0%	6.0%	2.0%	7.0%	0.293	4.3%	5.2%	**0.589**
	LABA/LAMA	13.0%	14.2%	14.9%	0.658	7.0%	5.0%	20.0%	16.0%	13.0%	15.0%	0.211	6.0%	16.2%	**0.005**
	LABA/LAMA/ICS	1.0%	1.6%	1.8%	0.504	0.0%	0.0%	0.0%	3.0%	1.0%	2.0%	0.168	0.0%	1.7%	**0.058**
	ICS	1.0%	0.0%	0.0%	0.677	0.0%	7.0%	0.0%	0.0%	0.0%	1.0%	**0.011**	1.8%		**0.086**
	Other	2.0%	0.0%	0.0%	0.216	0.0%	14.0%	0.0%	2.0%	2.0%		0.101	2.1%	1.8%	**0.901**
**Group B**	Short-acting inhaled bronchodilator as only medication	3.0%	3.0%	4.4%	0.413	3.0%	7.0%	0.0%	10.0%	1.0%	2.0%	**0.006**	2.4%	4.0%	**0.390**
	LAMA monotherapy	25.0%	25.1%	25.3%	0.960	33.0%	**33.0%**	14.0%	25.0%	23.0%	26.0%	0.237	26.7%	24.5%	**0.654**

	LABA monotherapy	11.0%	7.8%	16.8%	0.003	15.0%	9.0%	7.0%	13.0%	9.0%	11.0%	0.552	11.8%	10.6%	**0.699**
	LABA/ICS	8.0%	9.4%	6.4%	0.136	10.0%	11.0%	9.0%	7.0%	5.0%	13.0%	0.108	8.9%	8.0%	**0.660**
	LABA/LAMA	**45.0%**	46.8%	43.2%	0.531	**36.0%**	**34.0%**	**61.0%**	**37.0%**	**59.0%**	**35.0%**	**0.001**	43.8%	46.2%	**0.683**
	LABA/LAMA/ICS	5.0%	6.4%	3.4%	0.062	3.0%	3.0%	8.0%	8.0%	4.0%	6.0%	0.313	5.3%	5.4%	**0.936**
	ICS	0.0%	0.0%	0.0%	0.527	0.0%	4.0%	0.0%	0.0%	0.0%	1.0%	**0.007**	0.8%		**0.128**
	Other	1.0%	1.2%	0.0%	0.545	0.0%	0.0%	0.0%	0.0%	0.0%	6.0%	0.206	0.0%	1.1%	**0.617**
**Group C**	Short-acting inhaled bronchodilator as only medication	1.0%	1.5%	0.7%	0.438	1.0%	1.0%	0.0%	5.0%	0.0%	0.0%	**0.024**	0.0%	1.8%	**0.099**
	LAMA monotherapy	17.0%	16.4%	18.0%	0.768	23.0%	9.0%	6.0%	14.0%	22.0%	16.0%	0.281	21.2%	15.1%	**0.261**
	LABA monotherapy	3.0%	2.4%	3.8%	0.196	3.0%	6.0%	3.0%	3.0%	2.0%	4.0%	0.529	2.4%	3.1%	**0.533**
	LABA/ICS	18.0%	16.8%	20.0%	0.444	17.0%	**31.0%**	14.0%	16.0%	15.0%	27.0%	0.218	13.0%	20.1%	**0.096**
	LABA/LAMA	**42.0%**	43.0%	40.6%	0.669	**42.0%**	26.0%	**58.0%**	**37.0%**	**44.0%**	**37.0%**	0.159	46.9%	40.1%	**0.255**
	LABA/LAMA/ICS	18.0%	19.2%	16.7%	0.548	15.0%	26.0%	19.0%	25.0%	16.0%	15.0%	0.457	16.4%	19.2%	**0.497**
	ICS	0.0%	0.0%	0.0%	0.751	0.0%	1.0%	0.0%	0.0%	0.0%	1.0%	0.233	0.0%	0.0%	**0.628**
	Other	0.0%	0.0%	0.0%	0.381	0.0%	0.0%	0.0%	0.0%	0.0%	1.0%	0.127	0.0%	0.0%	**0.425**

**Group D**	Short-acting inhaled bronchodilator as only medication	0.0%	0.5%	0.0%	0.652	0.0%	0.0%	0.0%	2.0%	0.0%	0.0%	**0.011**	0.0%	0.6%	**0.216**
	LAMA monotherapy	3.0%	3.2%	3.6%	0.752	3.0%	5.0%		6.0%	3.0%	4.0%	0.184	3.1%	3.4%	**0.788**
	LABA monotherapy	1.0%	1.4%	1.1%	0.626	1.0%	1.0%		3.0%	1.0%	2.0%	0.082	0.8%	1.5%	**0.319**
	LABA/ICS	12.0%	11.6%	13.3%	0.591	12.0%	18.0%	13.0%	11.0%	8.0%	18.0%	0.417	12.0%	12.2%	**0.969**
	LABA/LAMA	33.0%	31.7%	35.4%	0.467	42.0%	19.0%	32.0%	22.0%	42.0%	23.0%	**0.006**	38.5%	30.5%	**0.123**
	LABA/LAMA/ICS	49.0%	50.2%	45.9%	0.464	42.0%	56.0%	54.0%	56.0%	47.0%	45.0%	0.533	45.1%	50.4%	**0.375**
	ICS	0.0%	0.0%	0.0%	0.490	0.0%	0.0%	0.0%	0.0%	0.0%	0.0%	0.303	0.0%	0.0%	**0.515**
	Other	1.0%	1.5%	0.0%	0.545	0.0%	1.0%	0.0%	0.0%	0.0%	7.0%	0.262	0.5%	1.4%	**0.617**

Supplementary Table 5. Clinicians' opinion on the use of fixed dose LAMA/LABA in COPD treatment. Global results and sub-analysis by medical speciality.

Items are displayed in order of higher frequency			Completely disagree (1-3)	Neutral (4-6)	Completely agree (7-9)	Don't know	p value
			%				
LABA/ICS should be the initial treatment for COPD maintenance therapy before LAMA/LABA	Total sample		78.0%	17.3%	3.1%	1.6%	0.943
	Medical speciality	General Practitioners	77.8%	17.8%	2.2%	2.2%	
		Pulmonologists	78.0%	17.1%	3.7%	1.2%	
LABA/ICS is a better choice to prevent exacerbations compared to LAMA/LABA	Total sample		55.1%	36.2%	8.7%		0.482
	Medical speciality	General Practitioners	62.2%	31.1%	6.7%		
		Pulmonologists	51.2%	39.0%	9.8%		
LAMA should be the initial treatment for COPD maintenance therapy before LAMA/LABA	Total sample		14.2%	30.7%	55.1%		0.115
	Medical speciality	General Practitioners	13.3%	20.0%	66.7%		
		Pulmonologists	14.6%	36.6%	48.8%		
LAMA/LABA improve quality of life compared to LABA/ICS	Total sample		3.1%	32.3%	63.8%	0.8%	0.218
	Medical speciality	General Practitioners	2.2%	22.2%	75.6%		
		Pulmonologists	3.7%	37.8%	57.3%	1.2%	
LAMA/LABA improve physical activity compared to LABA/ICS	Total sample		3.1%	27.6%	67.7%	1.6%	0.934
	Medical speciality	General Practitioners	2.2%	28.9%	66.7%	2.2%	
		Pulmonologists	3.7%	26.8%	68.3%	1.2%	
LAMA/LABA improve physical activity compared to LAMA monotherapy	Total sample		3.1%	22.8%	73.2%	0.8%	0.251
	Medical speciality	General Practitioners		24.4%	73.3%	2.2%	
		Pulmonologists	4.9%	22.0%	73.2%		
LAMA/LABA improve quality of life compared to LAMA monotherapy	Total sample		3.1%	16.5%	79.5%	0.8%	0.551
	Medical speciality	General Practitioners	2.2%	17.8%	77.8%	2.2%	
		Pulmonologists	3.7%	15.9%	80.5%		
LAMA/LABA improve breathlessness compared to LABA/ICS	Total sample		1.6%	14.2%	84.3%		0.420
	Medical speciality	General Practitioners	2.2%	8.9%	88.9%		
		Pulmonologists	1.2%	17.1%	81.7%		
For some patients, LAMA/LABA can be the initial COPD maintenance treatment	Total sample		4.7%	9.4%	85.0%	0.8%	0.368
	Medical speciality	General Practitioners	4.4%	13.3%	80.0%	2.2%	
		Pulmonologists	4.9%	7.3%	87.8%		
LAMA/LABA improve breathlessness compared to LAMA monotherapy	Total sample		3.9%	7.1%	88.2%	0.8%	0.444

	Medical speciality	General Practitioners	2.2%	8.9%	86.7%	2.2%	
		Pulmonologists					

Supplementary Table 6. Clinicians' opinion on the use of fixed dose LAMA/LABA in COPD treatment. Global results and sub-analysis by country.

		Completely disagree (1-3)	Neutral (4-6)	Completely agree (7-9)	Don't know	p value
Items are displayed in order of higher frequency		%				
LABA/ICS should be the initial treatment for COPD maintenance therapy before LAMA/LABA	Total sample	78.0%	17.3%	3.1%	1.6%	0.792
	Belgium	73.1%	15.4%	7.7%	3.8%	
	Finland	85.7%	14.3%			
	Greece	64.7%	35.3%			
	Norway	84.0%	16.0%			
	Portugal	79.4%	14.7%	2.9%	2.9%	
	The Netherlands	83.3%	11.1%	5.6%		
LABA/ICS is a better choice to prevent exacerbations compared to LAMA/LABA	Total sample	55.1%	36.2%	8.7%		0.180
	Belgium	61.5%	30.8%	7.7%		
	Finland	42.9%	28.6%	28.6%		
	Greece	47.1%	52.9%			
	Norway	40.0%	44.0%	16.0%		
	Portugal	70.6%	23.5%	5.9%		
	The Netherlands	50.0%	44.4%	5.6%		
LAMA should be the initial treatment for COPD maintenance therapy before LAMA/LABA	Total sample	14.2%	30.7%	55.1%		0.576
	Belgium	7.7%	26.9%	65.4%		
	Finland	14.3%	57.1%	28.6%		
	Greece	17.6%	47.1%	35.3%		
	Norway	20.0%	24.0%	56.0%		
	Portugal	11.8%	29.4%	58.8%		
	The Netherlands	16.7%	22.2%	61.1%		
LAMA/LABA improve quality of life compared to LABA/ICS	Total sample	3.1%	32.3%	63.8%	0.8%	0.203
	Belgium	3.8%	26.9%	69.2%		
	Finland	14.3%	42.9%	42.9%		
	Greece		35.3%	64.7%		
	Norway	8.0%	44.0%	48.0%		
	Portugal		20.6%	79.4%		
	The Netherlands		38.9%	55.6%	5.6%	
LAMA/LABA improve physical activity compared to LABA/ICS	Total sample	3.1%	27.6%	67.7%	1.6%	0.019
	Belgium	3.8%	34.6%	61.5%		
	Finland	14.3%	14.3%	57.1%	14.3%	

	Greece		23.5%	70.6%	5.9%	
	Norway	8.0%	48.0%	44.0%		
	Portugal		17.6%	82.4%		
	The Netherlands		16.7%	83.3%		
LAMA/LABA improve physical activity compared to LAMA monotherapy	Total sample	3.1%	22.8%	73.2%	0.8%	0.292
	Belgium	3.8%	34.6%	61.5%		
	Finland		14.3%	85.7%		
	Greece		11.8%	88.2%		
	Norway	8.0%	40.0%	52.0%		
	Portugal	2.9%	14.7%	79.4%	2.9%	
	The Netherlands		11.1%	88.9%		
LAMA/LABA improve quality of life compared to LAMA monotherapy	Total sample	3.1%	16.5%	79.5%	0.8%	0.436
	Belgium	3.8%	19.2%	76.9%		
	Finland		14.3%	85.7%		
	Greece	5.9%	11.8%	82.4%		
	Norway	8.0%	28.0%	64.0%		
	Portugal		8.8%	91.2%		
	The Netherlands		16.7%	77.8%	5.6%	
LAMA/LABA improve breathlessness compared to LABA/ICS	Total sample	1.6%	14.2%	84.3%		0.114
	Belgium		15.4%	84.6%		
	Finland	14.3%	14.3%	71.4%		
	Greece		23.5%	76.5%		
	Norway	4.0%	20.0%	76.0%		
	Portugal		2.9%	97.1%		
	The Netherlands		16.7%	83.3%		
For some patients, LAMA/LABA can be the initial COPD maintenance treatment	Total sample	4.7%	9.4%	85.0%	0.8%	0.062
	Belgium	15.4%	23.1%	61.5%		
	Finland		14.3%	85.7%		
	Greece			100.0%		
	Norway	8.0%	8.0%	84.0%		
	Portugal		2.9%	94.1%	2.9%	
	The Netherlands		11.1%	88.9%		
LAMA/LABA improve breathlessness compared to LAMA monotherapy	Total sample	3.9%	7.1%	88.2%	0.8%	0.415
	Belgium	7.7%		92.3%		
	Finland			100.0%		
	Greece		5.9%	94.1%		
	Norway	8.0%	16.0%	76.0%		

	Portugal	2.9%	2.9%	91.2%	2.9%	
	The Netherlands		16.7%	83.3%		

Supplementary Table 7. Clinicians' opinion on the risks and benefits of ICS treatment in COPD patients. Global results and sub-analysis by medical speciality.

			Completely disagree (1-3)	Neutral (4-6)	Completely agree (7-9)	Don't know	p value
			%				
**a. When considering ICS prescription, I take into account:** (*Items are displayed in order of higher frequency*)							
Comorbidities alone.	Total sample		70.1%	27.6%	2.4%		0.837
	Medical speciality	General Practitioners	73.3%	24.4%	2.2%		
		Pulmonologists	68.3%	29.3%	2.4%		
Blood eosinophil count alone.	Total sample		66.1%	22.0%	9.4%	2.4%	0.075
	Medical speciality	General Practitioners	68.9%	15.6%	8.9%	6.7%	
		Pulmonologists	64.6%	25.6%	9.8%		
Exacerbation risk alone.	Total sample		44.1%	45.7%	10.2%		0.374
	Medical speciality	General Practitioners	48.9%	37.8%	13.3%		
		Pulmonologists	41.5%	50.0%	8.5%		
Combination of exacerbation risk and blood eosinophil count.	Total sample		3.9%	26.0%	68.5%	1.6%	0.021
	Medical speciality	General Practitioners	6.7%	35.6%	53.3%	4.4%	
		Pulmonologists	2.4%	20.7%	76.8%		
Comorbidities, blood eosinophil count and exacerbation risks.	Total sample		3.9%	24.4%	70.1%	1.6%	0.243
	Medical speciality	General Practitioners	2.2%	24.4%	68.9%	4.4%	
		Pulmonologists	4.9%	24.4%	70.7%		
Uncertainty of concomitant asthma diagnosis.	Total sample		1.6%	21.3%	77.2%		0.887
	Medical speciality	General Practitioners	2.2%	20.0%	77.8%		

		Pulmonologists	1.2%	22.0%	76.8%		
Bruising negatively impacts quality of life of COPD patients treated with ICS	Total sample		15.7%	66.9%	12.6%	4.7%	0.030
	Medical speciality	General Practitioners	15.6%	73.3%	2.2%	8.9%	
		Pulmonologists	15.9%	63.4%	18.3%	2.4%	
ICS treatment increase the risk of diabetes.	Total sample		20.5%	63.8%	15.0%	0.8%	0.552
	Medical speciality	General Practitioners	22.2%	60.0%	15.6%	2.2%	
		Pulmonologists	19.5%	65.9%	14.6%		
Special attention should be given to the risk/benefit ratio of ICS treatment because it has a negative impact on diabetes control.	Total sample		20.5%	63.0%	15.7%	0.8%	0.293
	Medical speciality	General Practitioners	13.3%	73.3%	13.3%		
		Pulmonologists	24.4%	57.3%	17.1%	1.2%	
Treatment with ICS increases the risk of tuberculosis	Total sample		26.8%	50.4%	16.5%	6.3%	0.428
	Medical speciality	General Practitioners	24.4%	48.9%	15.6%	11.1%	
		Pulmonologists	28.0%	51.2%	17.1%	3.7%	
Long-term ICS treatment in patients with low blood eosinophil count increases the risk of exacerbations probably linked with changes in the airway microbiome.	Total sample		11.8%	56.7%	18.9%	12.6%	0.002
	Medical speciality	General Practitioners	6.7%	55.6%	11.1%	26.7%	
		Pulmonologists	14.6%	57.3%	23.2%	4.9%	
ICS side effects could be more prominent in patients with COPD who have no history of asthma.	Total sample		15.7%	56.7%	21.3%	6.3%	0.017
	Medical speciality	General Practitioners	13.3%	51.1%	20.0%	15.6%	
		Pulmonologists	17.1%	59.8%	22.0%	1.2%	
Special care should be taken with the prescription of ICS to a patient suffering from osteoporosis, since it could increase the risk for fractures by reducing bone density.	Total sample		8.7%	55.1%	35.4%	0.8%	0.474
	Medical speciality	General Practitioners	11.1%	51.1%	35.6%	2.2%	
		Pulmonologists	7.3%	57.3%	35.4%		

Oral candidiasis negatively impacts quality of life of COPD patients treated with ICS	Total sample		6.3%	48.0%	44.9%	0.8%	0.118
	Medical speciality	General Practitioners	11.1%	51.1%	35.6%	2.2%	
		Pulmonologists	3.7%	46.3%	50.0%		
Treatment with ICS is less beneficial when patients are smokers.	Total sample		11.0%	25.2%	56.7%	7.1%	0.002
	Medical speciality	General Practitioners	4.4%	28.9%	48.9%	17.8%	
		Pulmonologists	14.6%	23.2%	61.0%	1.2%	
Special attention should be given to the risk-benefit ratio of ICS in COPD patients with osteopenia / fractures	Total sample		6.3%	34.6%	59.1%		0.356
	Medical speciality	General Practitioners	8.9%	40.0%	51.1%		
		Pulmonologists	4.9%	31.7%	63.4%		
Bruising is not uncommon in ICS-treated patients	Total sample		11.8%	26.0%	59.1%	3.1%	0.143
	Medical speciality	General Practitioners	8.9%	33.3%	51.1%	6.7%	
		Pulmonologists	13.4%	22.0%	63.4%	1.2%	
Treatment with ICS increases the risk of pneumonia.	Total sample		3.9%	26.8%	69.3%		0.483
	Medical speciality	General Practitioners	2.2%	22.2%	75.6%		
		Pulmonologists	4.9%	29.3%	65.9%		
Oral candidiasis is not uncommon in ICS-treated patients	Total sample		4.7%	22.8%	72.4%		0.667
	Medical speciality	General Practitioners	6.7%	20.0%	73.3%		
		Pulmonologists	3.7%	24.4%	72.0%		
It is good to re-assess older ICS prescriptions to see if it is still indicated and/or efficacious.	Total sample		6.3%	18.1%	73.2%	2.4%	0.119
	Medical speciality	General Practitioners		20.0%	75.6%	4.4%	
		Pulmonologists	9.8%	17.1%	72.0%	1.2%	
	Total sample		3.1%	18.1%	78.7%		0.760

Co-morbidities should be considered when selecting COPD treatment	Medical speciality	General Practitioners	2.2%	15.6%	82.2%		
		Pulmonologists	3.7%	19.5%	76.8%		
**LABA/ICS may decrease exacerbations to a greater extent than a LABA/LAMA combination for patients with:** *(Items are displayed in order of higher frequency)*							
Low exacerbation risk & low eosinophil blood concentrations	Total sample		78.7%	14.2%	4.7%	2.4%	0.721
	Medical speciality	General Practitioners	77.8%	13.3%	4.4%	4.4%	
		Pulmonologists	79.3%	14.6%	4.9%	1.2%	
High exacerbation risk & low eosinophil blood concentrations	Total sample		32.3%	52.8%	12.6%	2.4%	0.103
	Medical speciality	General Practitioners	26.7%	53.3%	13.3%	6.7%	
		Pulmonologists	35.4%	52.4%	12.2%		
Low exacerbation risk & high eosinophil blood concentrations	Total sample		11.8%	56.7%	28.3%	3.1%	0.015
	Medical speciality	General Practitioners	11.1%	62.2%	17.8%	8.9%	
		Pulmonologists	12.2%	53.7%	34.1%		
High exacerbation risk (>2 exacerbations and/or 1 hospitalization in the previous year) & high eosinophil blood concentrations	Total sample		4.7%	12.6%	81.1%	1.6%	0.723
	Medical speciality	General Practitioners	2.2%	11.1%	84.4%	2.2%	
		Pulmonologists	6.1%	13.4%	79.3%	1.2%	

Supplementary Table 8. Clinicians' opinion on the risks and benefits of ICS treatment in COPD patients. Global results and sub-analysis by country.

		Completely disagree (1-3)	Neutral (4-6)	Completely agree (7-9)	Don't know	
		%				p value
When considering ICS prescription, I take into account: *(Items are displayed in order of higher frequency)*						
Comorbidities alone.		70.1%	27.6%	2.4%		0.142
	Belgium	53.8%	46.2%			
	Finland	85.7%	14.3%			
	Greece	58.8%	35.3%	5.9%		
	Norway	84.0%	16.0%			
	Portugal	82.4%	14.7%	2.9%		
	The Netherlands	55.6%	38.9%	5.6%		
Blood eosinophil count alone.		66.1%	22.0%	9.4%	2.4%	0.001
	Belgium	57.7%	26.9%	11.5%	3.8%	
	Finland	57.1%	28.6%	14.3%		
	Greece	52.9%	47.1%			
	Norway	76.0%	20.0%	4.0%		
	Portugal	88.2%	8.8%		2.9%	
	The Netherlands	38.9%	16.7%	38.9%	5.6%	
Exacerbation risk alone.		44.1%	45.7%	10.2%		0.007
	Belgium	38.5%	57.7%	3.8%		
	Finland	71.4%	14.3%	14.3%		
	Greece	23.5%	70.6%	5.9%		
	Norway	60.0%	32.0%	8.0%		
	Portugal	52.9%	41.2%	5.9%		
	The Netherlands	22.2%	44.4%	33.3%		
Combination of exacerbation risk and blood eosinophil count.		3.9%	26.0%	68.5%	1.6%	0.047
	Belgium		26.9%	69.2%	3.8%	
	Finland		28.6%	71.4%		
	Greece	5.9%	41.2%	52.9%		
	Norway	16.0%	36.0%	48.0%		
	Portugal		14.7%	85.3%		
	The Netherlands		16.7%	77.8%	5.6%	
Comorbidities, blood eosinophil count and exacerbation risks.		3.9%	24.4%	70.1%	1.6%	0.562
	Belgium	3.8%	23.1%	69.2%	3.8%	

	Finland			100.0%		
	Greece	5.9%	29.4%	64.7%		
	Norway	8.0%	36.0%	56.0%		
	Portugal		17.6%	82.4%		
	The Netherlands	5.6%	27.8%	61.1%	5.6%	
Uncertainty of concomitant asthma diagnosis.		1.6%	21.3%	77.2%		0.002
	Belgium		26.9%	73.1%		
	Finland			100.0%		
	Greece		11.8%	88.2%		
	Norway	8.0%	48.0%	44.0%		
	Portugal		8.8%	91.2%		
	The Netherlands		16.7%	83.3%		
Bruising negatively impacts quality of life of COPD patients treated with ICS		15.7%	66.9%	12.6%	4.7%	0.201
	Belgium	11.5%	61.5%	15.4%	11.5%	
	Finland	14.3%	71.4%	14.3%		
	Greece	35.3%	52.9%	11.8%		
	Norway	16.0%	72.0%	12.0%		
	Portugal	17.6%	73.5%	2.9%	5.9%	
	The Netherlands		66.7%	27.8%	5.6%	
ICS treatment increase the risk of diabetes.		20.5%	63.8%	15.0%	0.8%	0.839
	Belgium	23.1%	57.7%	19.2%		
	Finland	14.3%	57.1%	28.6%		
	Greece	23.5%	52.9%	23.5%		
	Norway	24.0%	64.0%	8.0%	4.0%	
	Portugal	20.6%	67.6%	11.8%		
	The Netherlands	11.1%	77.8%	11.1%		
Special attention should be given to the risk/benefit ratio of ICS treatment because it has a negative impact on diabetes control.		20.5%	63.0%	15.7%	0.8%	0.348
	Belgium	26.9%	57.7%	15.4%		
	Finland	14.3%	42.9%	42.9%		
	Greece	23.5%	47.1%	23.5%	5.9%	
	Norway	28.0%	60.0%	12.0%		
	Portugal	11.8%	76.5%	11.8%		
	The Netherlands	16.7%	72.2%	11.1%		
Treatment with ICS increases the risk of tuberculosis		26.8%	50.4%	16.5%	6.3%	0.505
	Belgium	30.8%	42.3%	23.1%	3.8%	
	Finland	14.3%	71.4%	14.3%		
	Greece	17.6%	52.9%	29.4%		
	Norway	32.0%	52.0%	8.0%	8.0%	

	Portugal	20.6%	55.9%	17.6%	5.9%	
	The Netherlands	38.9%	38.9%	5.6%	16.7%	
Long-term ICS treatment in patients with low blood eosinophil count increases the risk of exacerbations probably linked with changes in the airway microbiome.		11.8%	56.7%	18.9%	12.6%	0.457
	Belgium	15.4%	50.0%	19.2%	15.4%	
	Finland	14.3%	28.6%	42.9%	14.3%	
	Greece	5.9%	58.8%	35.3%		
	Norway	20.0%	56.0%	12.0%	12.0%	
	Portugal	5.9%	67.6%	14.7%	11.8%	
	The Netherlands	11.1%	55.6%	11.1%	22.2%	
ICS side effects could be more prominent in patients with COPD who have no history of asthma.		15.7%	56.7%	21.3%	6.3%	0.268
	Belgium	11.5%	53.8%	23.1%	11.5%	
	Finland		57.1%	42.9%		
	Greece	35.3%	41.2%	23.5%		
	Norway	28.0%	56.0%	12.0%	4.0%	
	Portugal	5.9%	67.6%	20.6%	5.9%	
	The Netherlands	11.1%	55.6%	22.2%	11.1%	
Special care should be taken with the prescription of ICS to a patient suffering from osteoporosis, since it could increase the risk for fractures by reducing bone density.		8.7%	55.1%	35.4%	0.8%	0.609
	Belgium	19.2%	50.0%	26.9%	3.8%	
	Finland		71.4%	28.6%		
	Greece		64.7%	35.3%		
	Norway	8.0%	44.0%	48.0%		
	Portugal	5.9%	58.8%	35.3%		
	The Netherlands	11.1%	55.6%	33.3%		
Oral candidiasis negatively impacts quality of life of COPD patients treated with ICS		6.3%	48.0%	44.9%	0.8%	0.666
	Belgium	7.7%	38.5%	53.8%		
	Finland	14.3%	42.9%	42.9%		
	Greece	5.9%	47.1%	47.1%		
	Norway	12.0%	44.0%	44.0%		
	Portugal	2.9%	58.8%	38.2%		
	The Netherlands		50.0%	44.4%	5.6%	
Treatment with ICS is less beneficial when patients are smokers.		11.0%	25.2%	56.7%	7.1%	0.266
	Belgium	19.2%	26.9%	42.3%	11.5%	
	Finland	14.3%	14.3%	71.4%		
	Greece	23.5%	35.3%	41.2%		
	Norway	16.0%	24.0%	52.0%	8.0%	
	Portugal		20.6%	70.6%	8.8%	
	The Netherlands		27.8%	66.7%	5.6%	

Special attention should be given to the risk-benefit ratio of ICS in COPD patients with osteopenia / fractures		6.3%	34.6%	59.1%		0.444
	Belgium	15.4%	42.3%	42.3%		
	Finland		28.6%	71.4%		
	Greece		23.5%	76.5%		
	Norway	8.0%	40.0%	52.0%		
	Portugal	2.9%	35.3%	61.8%		
	The Netherlands	5.6%	27.8%	66.7%		
Bruising is not uncommon in ICS-treated patients		11.8%	26.0%	59.1%	3.1%	0.011
	Belgium	7.7%	34.6%	46.2%	11.5%	
	Finland			100.0%		
	Greece	29.4%	35.3%	35.3%		
	Norway	4.0%	24.0%	72.0%		
	Portugal	20.6%	26.5%	50.0%	2.9%	
	The Netherlands		16.7%	83.3%		
Treatment with ICS increases the risk of pneumonia.		3.9%	26.8%	69.3%		0.500
	Belgium	3.8%	30.8%	65.4%		
	Finland		42.9%	57.1%		
	Greece	5.9%	41.2%	52.9%		
	Norway	8.0%	28.0%	64.0%		
	Portugal		14.7%	85.3%		
	The Netherlands	5.6%	22.2%	72.2%		
Oral candidiasis is not uncommon in ICS-treated patients		4.7%	22.8%	72.4%		0.275
	Belgium	3.8%	38.5%	57.7%		
	Finland		14.3%	85.7%		
	Greece	11.8%	35.3%	52.9%		
	Norway	4.0%	20.0%	76.0%		
	Portugal	5.9%	11.8%	82.4%		
	The Netherlands		16.7%	83.3%		
It is good to re-assess older ICS prescriptions to see if it is still indicated and/or efficacious.		6.3%	18.1%	73.2%	2.4%	0.281
	Belgium	7.7%	7.7%	84.6%		
	Finland	14.3%	14.3%	71.4%		
	Greece	11.8%	35.3%	52.9%		
	Norway	12.0%	24.0%	60.0%	4.0%	
	Portugal		20.6%	76.5%	2.9%	
	The Netherlands		5.6%	88.9%	5.6%	
Co-morbidities should be considered when selecting COPD treatment		3.1%	18.1%	78.7%		0.006
	Belgium	3.8%	46.2%	50.0%		

	Finland			100.0%		
	Greece	5.9%	5.9%	88.2%		
	Norway	8.0%	20.0%	72.0%		
	Portugal		8.8%	91.2%		
	The Netherlands		11.1%	88.9%		
LABA/ICS may decrease exacerbations to a greater extent than a LABA/LAMA combination for patients with: *(Items are displayed in order of higher frequency)*						
Low exacerbation risk & low eosinophil blood concentrations		78.7%	14.2%	4.7%	2.4%	0.187
	Belgium	84.6%	7.7%	3.8%	3.8%	
	Finland	85.7%			14.3%	
	Greece	58.8%	29.4%	11.8%		
	Norway	76.0%	16.0%	4.0%	4.0%	
	Portugal	88.2%	5.9%	5.9%		
	The Netherlands	72.2%	27.8%			
High exacerbation risk & low eosinophil blood concentrations		32.3%	52.8%	12.6%	2.4%	0.382
	Belgium	42.3%	42.3%	11.5%	3.8%	
	Finland	42.9%	42.9%	14.3%		
	Greece	35.3%	64.7%			
	Norway	24.0%	56.0%	20.0%		
	Portugal	35.3%	50.0%	14.7%		
	The Netherlands	16.7%	61.1%	11.1%	11.1%	
Low exacerbation risk & high eosinophil blood concentrations		11.8%	56.7%	28.3%	3.1%	0.547
	Belgium	15.4%	53.8%	26.9%	3.8%	
	Finland		42.9%	57.1%		
	Greece	11.8%	52.9%	35.3%		
	Norway	20.0%	60.0%	16.0%	4.0%	
	Portugal	8.8%	64.7%	26.5%		
	The Netherlands	5.6%	50.0%	33.3%	11.1%	
High exacerbation risk (>2 exacerbations and/or 1 hospitalization in the previous year) & high eosinophil blood concentrations		4.7%	12.6%	81.1%	1.6%	0.588
	Belgium	7.7%	11.5%	73.1%	7.7%	
	Finland			100.0%		
	Greece	5.9%	23.5%	70.6%		
	Norway	4.0%	12.0%	84.0%		
	Portugal	2.9%	14.7%	82.4%		
	The Netherlands	5.6%	5.6%	88.9%		

Supplementary Table 9. Importance of the criteria for the selection of an initial COPD treatment. Global results and sub-analysis by medical speciality.

			Not important (1-3)	Neutral (4-6)	Important (7-9)	Don't know	
Criteria for the selection of an initial COPD treatment<br><br>*(Items are displayed in order of appearance at the questionnaire)*			%				p value
Age	Total sample		52.8%	33.9%	12.6%	0.8%	0.324
	Medical speciality	General Practitioners	55.6%	26.7%	15.6%	2.2%	
		Pulmonologists	51.2%	37.8%	11.0%		
Smoking status	Total sample		22.8%	35.4%	41.7%		0.691
	Medical speciality	General Practitioners	20.0%	33.3%	46.7%		
		Pulmonologists	24.4%	36.6%	39.0%		
Breathlessness	Total sample		3.9%	7.1%	89.0%		0.657
	Medical speciality	General Practitioners	2.2%	8.9%	88.9%		
		Pulmonologists	4.9%	6.1%	89.0%		
Exercise limitation	Total sample		4.7%	22.0%	72.4%	0.8%	0.045
	Medical speciality	General Practitioners		31.1%	66.7%	2.2%	
		Pulmonologists	7.3%	17.1%	75.6%		
Previous exacerbations	Total sample		2.4%	5.5%	92.1%		0.213
	Medical speciality	General Practitioners		8.9%	91.1%		
		Pulmonologists	3.7%	3.7%	92.7%		
History of pneumonia	Total sample		9.4%	29.9%	60.6%		0.506
	Medical speciality	General Practitioners	13.3%	26.7%	60.0%		
		Pulmonologists	7.3%	31.7%	61.0%		
Blood eosinophil count	Total sample		7.1%	26.8%	65.4%	0.8%	0.356
	Medical speciality	General Practitioners	8.9%	31.1%	57.8%	2.2%	
		Pulmonologists	6.1%	24.4%	69.5%		
Cardiovascular	Total sample		10.2%	33.1%	55.9%	0.8%	0.268
	Medical speciality	General Practitioners	11.1%	24.4%	62.2%	2.2%	
		Pulmonologists	9.8%	37.8%	52.4%		

Diabetes	Total sample		14.2%	61.4%	23.6%	0.8%	0.228
	Medical speciality	General Practitioners	11.1%	55.6%	31.1%	2.2%	
		Pulmonologists	15.9%	64.6%	19.5%		
Osteoporosis/osteopenia	Total sample		18.9%	58.3%	22.0%	0.8%	0.232
	Medical speciality	General Practitioners	24.4%	57.8%	15.6%	2.2%	
		Pulmonologists	15.9%	58.5%	25.6%		
Current asthma	Total sample		4.7%	7.9%	87.4%		0.395
	Medical speciality	General Practitioners	2.2%	11.1%	86.7%		
		Pulmonologists	6.1%	6.1%	87.8%		
History of asthma	Total sample		5.5%	21.3%	73.2%		0.014
	Medical speciality	General Practitioners	4.4%	35.6%	60.0%		
		Pulmonologists	6.1%	13.4%	80.5%		
Anxiety/depression	Total sample		22.8%	59.1%	16.5%	1.6%	0.862
	Medical speciality	General Practitioners	22.2%	62.2%	13.3%	2.2%	
		Pulmonologists	23.2%	57.3%	18.3%	1.2%	
Dementia	Total sample		11.0%	48.8%	39.4%	0.8%	0.042
	Medical speciality	General Practitioners	20.0%	46.7%	31.1%	2.2%	
		Pulmonologists	6.1%	50.0%	43.9%		
Osteoarthritis / degenerative joint disease	Total sample		43.3%	44.9%	9.4%	2.4%	0.657
	Medical speciality	General Practitioners	42.2%	42.2%	11.1%	4.4%	
		Pulmonologists	43.9%	46.3%	8.5%	1.2%	
The type of inhaler	Total sample		2.4%	16.5%	80.3%	0.8%	0.306
	Medical speciality	General Practitioners	2.2%	22.2%	73.3%	2.2%	
		Pulmonologists	2.4%	13.4%	84.1%		
Posology	Total sample		14.2%	34.6%	43.3%	7.9%	0.444
	Medical speciality	General Practitioners	8.9%	42.2%	42.2%	6.7%	
		Pulmonologists	17.1%	30.5%	43.9%	8.5%	
Ability to inhale	Total sample		1.6%	3.9%	93.7%	0.8%	0.633
	Medical speciality	General Practitioners		4.4%	95.6%		
		Pulmonologists	2.4%	3.7%	92.7%	1.2%	

Carbon footprint	Total sample		44.1%	38.6%	14.2%	3.1%	0.185
	Medical speciality	General Practitioners	55.6%	31.1%	8.9%	4.4%	
		Pulmonologists	37.8%	42.7%	17.1%	2.4%	
Price of the medicine	Total sample		26.0%	48.8%	24.4%	0.8%	0.333
	Medical speciality	General Practitioners	22.2%	55.6%	20.0%	2.2%	
		Pulmonologists	28.0%	45.1%	26.8%		
Reimbursement criteria	Total sample		23.6%	26.8%	44.9%	4.7%	0.050
	Medical speciality	General Practitioners	15.6%	40.0%	37.8%	6.7%	
		Pulmonologists	28.0%	19.5%	48.8%	3.7%	
GOLD Report	Total sample		5.5%	22.8%	70.9%	0.8%	0.196
	Medical speciality	General Practitioners		24.4%	75.6%		
		Pulmonologists	8.5%	22.0%	68.3%	1.2%	
National/local guidelines	Total sample		8.7%	16.5%	73.2%	1.6%	0.040
	Medical speciality	General Practitioners	4.4%	8.9%	82.2%	4.4%	
		Pulmonologists	11.0%	20.7%	68.3%		

Supplementary Table 10. Importance of the criteria for the selection of an initial COPD treatment. Global results and sub-analysis by country.

	Country	Not important (1-3)	Neutral (4-6)	Important (7-9)	Don't know	
Criteria for the selection of an initial COPD treatment<br><br>*(Items are displayed in order of appearance at the questionnaire)*		%				p value
Age	Total sample	52.8%	33.9%	12.6%	0.8%	0.523
	Belgium	69.2%	26.9%	3.8%		
	Finland	28.6%	42.9%	28.6%		
	Greece	35.3%	47.1%	17.6%		
	Norway	52.0%	36.0%	8.0%	4.0%	
	Portugal	55.9%	32.4%	11.8%		
	The Netherlands	50.0%	27.8%	22.2%		
Smoking status	Total sample	22.8%	35.4%	41.7%		0.208
	Belgium	23.1%	38.5%	38.5%		
	Finland		42.9%	57.1%		
	Greece	23.5%	29.4%	47.1%		
	Norway	44.0%	20.0%	36.0%		
	Portugal	17.6%	47.1%	35.3%		
	The Netherlands	11.1%	33.3%	55.6%		
Breathlessness	Total sample	3.9%	7.1%	89.0%		0.000
	Belgium	7.7%		92.3%		
	Finland		42.9%	57.1%		
	Greece			100.0%		
	Norway	8.0%	24.0%	68.0%		
	Portugal	2.9%		97.1%		
	The Netherlands			100.0%		
Exercise limitation	Total sample	4.7%	22.0%	72.4%	0.8%	0.167
	Belgium	11.5%	23.1%	65.4%		
	Finland		28.6%	71.4%		
	Greece		17.6%	82.4%		
	Norway	12.0%	36.0%	48.0%	4.0%	
	Portugal		14.7%	85.3%		
	The Netherlands		16.7%	83.3%		
Previous exacerbations	Total sample	2.4%	5.5%	92.1%		0.380

	Belgium	7.7%		92.3%		
	Finland			100.0%		
	Greece		5.9%	94.1%		
	Norway	4.0%	12.0%	84.0%		
	Portugal		2.9%	97.1%		
	The Netherlands		11.1%	88.9%		
History of pneumonia	Total sample	9.4%	29.9%	60.6%		0.006
	Belgium		46.2%	53.8%		
	Finland	14.3%		85.7%		
	Greece	23.5%	47.1%	29.4%		
	Norway	20.0%	24.0%	56.0%		
	Portugal	2.9%	17.6%	79.4%		
	The Netherlands	5.6%	33.3%	61.1%		
Blood eosinophil count	Total sample	7.1%	26.8%	65.4%	0.8%	0.629
	Belgium	3.8%	34.6%	61.5%		
	Finland			100.0%		
	Greece	11.8%	29.4%	58.8%		
	Norway	12.0%	36.0%	52.0%		
	Portugal	2.9%	20.6%	73.5%	2.9%	
	The Netherlands	11.1%	22.2%	66.7%		
Cardiovascular	Total sample	10.2%	33.1%	55.9%	0.8%	0.005
	Belgium	11.5%	50.0%	38.5%		
	Finland		28.6%	71.4%		
	Greece		47.1%	52.9%		
	Norway	28.0%	40.0%	28.0%	4.0%	
	Portugal	5.9%	20.6%	73.5%		
	The Netherlands	5.6%	11.1%	83.3%		
Diabetes	Total sample	14.2%	61.4%	23.6%	0.8%	0.297
	Belgium	15.4%	65.4%	19.2%		
	Finland		57.1%	42.9%		
	Greece	11.8%	64.7%	23.5%		
	Norway	32.0%	48.0%	16.0%	4.0%	
	Portugal	2.9%	67.6%	29.4%		
	The Netherlands	16.7%	61.1%	22.2%		
Osteoporosis/osteopenia	Total sample	18.9%	58.3%	22.0%	0.8%	0.482
	Belgium	23.1%	53.8%	23.1%		
	Finland		57.1%	42.9%		

	Greece	5.9%	70.6%	23.5%		
	Norway	36.0%	56.0%	8.0%		
	Portugal	14.7%	58.8%	23.5%	2.9%	
	The Netherlands	16.7%	55.6%	27.8%		
Current asthma	Total sample	4.7%	7.9%	87.4%		0.420
	Belgium	7.7%	15.4%	76.9%		
	Finland			100.0%		
	Greece		11.8%	88.2%		
	Norway	12.0%	4.0%	84.0%		
	Portugal		5.9%	94.1%		
	The Netherlands	5.6%	5.6%	88.9%		
History of asthma	Total sample	5.5%	21.3%	73.2%		0.057
	Belgium	19.2%	26.9%	53.8%		
	Finland			100.0%		
	Greece		23.5%	76.5%		
	Norway	8.0%	16.0%	76.0%		
	Portugal		23.5%	76.5%		
	The Netherlands		22.2%	77.8%		
Anxiety/depression	Total sample	22.8%	59.1%	16.5%	1.6%	0.054
	Belgium	34.6%	53.8%	11.5%		
	Finland		71.4%	28.6%		
	Greece	11.8%	70.6%	17.6%		
	Norway	40.0%	60.0%			
	Portugal	20.6%	58.8%	17.6%	2.9%	
	The Netherlands	5.6%	50.0%	38.9%	5.6%	
Dementia	Total sample	11.0%	48.8%	39.4%	0.8%	0.336
	Belgium	3.8%	57.7%	38.5%		
	Finland		42.9%	57.1%		
	Greece	5.9%	52.9%	41.2%		
	Norway	24.0%	28.0%	48.0%		
	Portugal	5.9%	55.9%	35.3%	2.9%	
	The Netherlands	22.2%	50.0%	27.8%		
Osteoarthritis / degenerative joint disease	Total sample	43.3%	44.9%	9.4%	2.4%	0.394
	Belgium	53.8%	38.5%	7.7%		
	Finland	28.6%	57.1%	14.3%		
	Greece	47.1%	52.9%			
	Norway	44.0%	48.0%	8.0%		

	Portugal	32.4%	47.1%	17.6%	2.9%	
	The Netherlands	50.0%	33.3%	5.6%	11.1%	
The type of inhaler	Total sample	2.4%	16.5%	80.3%	0.8%	0.009
	Belgium	3.8%	30.8%	65.4%		
	Finland			100.0%		
	Greece			100.0%		
	Norway	8.0%	32.0%	60.0%		
	Portugal		2.9%	97.1%		
	The Netherlands		22.2%	72.2%	5.6%	
Posology	Total sample	14.2%	34.6%	43.3%	7.9%	0.000
	Belgium	15.4%	53.8%	30.8%		
	Finland		14.3%	85.7%		
	Greece	5.9%	35.3%	58.8%		
	Norway	36.0%	36.0%	12.0%	16.0%	
	Portugal		23.5%	73.5%	2.9%	
	The Netherlands	22.2%	33.3%	16.7%	27.8%	
Ability to inhale	Total sample	1.6%	3.9%	93.7%	0.8%	0.538
	Belgium	3.8%	7.7%	88.5%		
	Finland			100.0%		
	Greece		5.9%	94.1%		
	Norway	4.0%	8.0%	88.0%		
	Portugal			100.0%		
	The Netherlands			94.4%	5.6%	
Carbon footprint	Total sample	44.1%	38.6%	14.2%	3.1%	0.008
	Belgium	61.5%	23.1%	15.4%		
	Finland		71.4%	28.6%		
	Greece	23.5%	64.7%	11.8%		
	Norway	72.0%	24.0%		4.0%	
	Portugal	35.3%	44.1%	17.6%	2.9%	
	The Netherlands	33.3%	33.3%	22.2%	11.1%	
Price of the medicine	Total sample	26.0%	48.8%	24.4%	0.8%	0.005
	Belgium	19.2%	53.8%	26.9%		
	Finland		71.4%	28.6%		
	Greece	35.3%	47.1%	17.6%		
	Norway	52.0%	44.0%	4.0%		
	Portugal	5.9%	52.9%	41.2%		
	The Netherlands	38.9%	33.3%	22.2%	5.6%	

Reimbursement criteria	Total sample	23.6%	26.8%	44.9%	4.7%	0.037
	Belgium	19.2%	30.8%	50.0%		
	Finland		28.6%	71.4%		
	Greece	23.5%	23.5%	47.1%	5.9%	
	Norway	40.0%	20.0%	40.0%		
	Portugal	8.8%	38.2%	47.1%	5.9%	
	The Netherlands	44.4%	11.1%	27.8%	16.7%	
GOLD Report	Total sample	5.5%	22.8%	70.9%	0.8%	0.184
	Belgium	11.5%	15.4%	69.2%	3.8%	
	Finland	14.3%	28.6%	57.1%		
	Greece		17.6%	82.4%		
	Norway	8.0%	36.0%	56.0%		
	Portugal		11.8%	88.2%		
	The Netherlands	5.6%	38.9%	55.6%		
National/local guidelines	Total sample	8.7%	16.5%	73.2%	1.6%	0.061
	Belgium	7.7%	30.8%	61.5%		
	Finland		14.3%	85.7%		
	Greece	17.6%	17.6%	64.7%		
	Norway	24.0%	8.0%	68.0%		
	Portugal		17.6%	79.4%	2.9%	
	The Netherlands		5.6%	88.9%	5.6%	

Supplementary Table 11. Multinomial logistic regression model showing the variables associated with the initial treatment decision.

**Criteria**	**Variables**	**LAMA monotherapy**		**LABA/LAMA**		**LABA/ICS**		**TT (LAMA/LABA/ICS)**	
		**OR**	**P**	**OR**	**P**	**OR**	**P**	**OR**	**p**
**Lung function**	FEV<50%	0.634	0.19	1.001	0.999	0.75	0.409	1.574	0.188
	FEV≥50%								
**Symptoms**	Severe	**0.307**	**0.014**	**2.632**	**0.031**	1.221	0.669	**9.955**	**<0.0001**
	Moderate	0.489	0.067	1.56	0.237	0.686	0.34	**2.623**	**0.017**
	Mild								
**Number of exacerbations in the last year**	1 or more with hospitalisation	**0.201**	**<0.0001**	0.857	0.711	**2.564**	**0.031**	**7.733**	**<0.0001**
	1 or more without hospitalisation	0.445	0.056	0.941	0.881	1.637	0.254	**4.126**	**0.001**
	None								
**Blood eosinophil count**	<100 eos / µl	0.816	0.679	0.873	0.774	0.516	0.224	0.499	0.168
	100–300 eos / µl	1.344	0.643	1.6	0.451	3.017	0.094	3.085	0.078
	≥ 300 eos / µl	**0.185**	**0.001**	**0.18**	**<0.0001**	**2.999**	**0.024**	1.468	0.418
	Unknown blood eosinophil count								

Supplementary Table 12. Relevant criteria for the initial treatment decision - Univariate analysis.

	LAMA monotherapy (N=292)	LAMA/LABA (N=741)	LABA/ICS (N=243)	LAMA/LABA/ICS (N=382)	Others (N=31)	p value
**Smoking status**						0.793
Current smoker	46.2%	50.1%	50.6%	49.7%	58.1%	
Non-smoker / Former smoker	53.8%	49.9%	49.4%	50.3%	41.9%	
**Lung function**						<0.0001
FEV1 <50%	42.1%	51.4%	43.6%	60.7%	35.5%	
FEV1 ≥50%	57.9%	48.6%	56.4%	39.3%	64.5%	
**Symptoms (cough, sputum, breathlessness)**						<0.0001
Mild	60.3%	26.9%	45.7%	13.6%	58.1%	
Moderate	28.1%	36.3%	29.2%	30.1%	32.3%	
Severe	11.6%	36.8%	25.1%	56.3%	9.7%	
**Number of exacerbations in the last year**						<0.0001
None	56.2%	37.4%	28.4%	12.8%	35.5%	
1 or more without hospitalisation	29.8%	31.4%	30.5%	32.5%	35.5%	

1 or more with hospitalisation	14.0%	31.2%	41.2%	54.7%	29.0%	
**Blood eosinophil count**						<0.0001
<100 eosinophils/μl	31.2%	36.2%	9.1%	14.9%	32.3%	
100-300 eosinophils/μl	24.0%	23.8%	17.3%	26.2%	19.4%	
≥ 300 eosinophils/μl	15.1%	10.8%	60.9%	39.5%	16.1%	

From the multinomial logistic regression model, the following assessments were gathered:

- The presence of severe symptoms, 1 or more exacerbations leading to hospitalization and >300 eos/μl are associated to the decision of not prescribing LAMA monotherapy.
- The presence of severe symptoms associated to LABA/LAMA prescription and >300 eos/μl are associated to the decision of not prescribing LAMA/LABA.
- The presence of 1 or more exacerbations leading to hospitalization and >300 eos/μl are associated to a LABA/ICS prescription.
- The presence of severe or moderate symptoms, 1 or more exacerbations leading to hospitalisation are associated to the prescription of triple therapy.

Supplementary Table 13. Opinion on the current use of inhaled corticosteroids. Global results and sub-analysis by medical specialty.

			Completely disagree (1-3)	Neutral (4-6)	Completely agree (7-9)	Don't know	p value
(Items are displayed in order of higher frequency)			%				
ICS are not over-prescribed in the primary care setting.	Total sample		79.5%	12.6%	7.9%		0.220
	Medical speciality	General Practitioners	71.1%	17.8%	11.1%		
		Pulmonologists	84.1%	9.8%	6.1%		
There is not enough evidence on the benefits of ICS withdrawal.	Total sample		60.6%	29.1%	8.7%	1.6%	0.010
	Medical speciality	General Practitioners	80.0%	15.6%	4.4%		
		Pulmonologists	50.0%	36.6%	11.0%	2.4%	
ICS are not over-prescribed in the specialty care.	Total sample		33.9%	51.2%	13.4%	1.6%	0.680
	Medical speciality	General Practitioners	40.0%	46.7%	11.1%	2.2%	
		Pulmonologists	30.5%	53.7%	14.6%	1.2%	
Pulmonologists feel insecure with ICS withdrawal.	Total sample		20.5%	59.1%	14.2%	6.3%	0.001
	Medical speciality	General Practitioners	15.6%	55.6%	11.1%	17.8%	
		Pulmonologists	23.2%	61.0%	15.9%		
The health care system facilitates ICS prescription in comparison to other treatments	Total sample		54.3%	23.6%	15.7%	6.3%	0.011
	Medical speciality	General Practitioners	73.3%	17.8%	6.7%	2.2%	
		Pulmonologists	43.9%	26.8%	20.7%	8.5%	
Physicians are familiar with international recommendations regarding ICS treatment restrictions.	Total sample		18.9%	54.3%	25.2%	1.6%	0.421
	Medical speciality	General Practitioners	17.8%	46.7%	33.3%	2.2%	
		Pulmonologists	19.5%	58.5%	20.7%	1.2%	
Local and national guidelines are timely updated according to latest evidence.	Total sample		15.7%	29.1%	54.3%	0.8%	0.685
	Medical speciality	General Practitioners	15.6%	24.4%	60.0%		
		Pulmonologists	15.9%	31.7%	51.2%	1.2%	

GPs feel insecure with ICS withdrawal.	Total sample		5.5%	20.5%	70.9%	3.1%	0.845
	Medical speciality	General Practitioners	6.7%	22.2%	66.7%	4.4%	
		Pulmonologists	4.9%	19.5%	73.2%	2.4%	
There is enough time in daily clinical practice to…<br>*(Items are displayed in order of frequency)*							
to assess factors that indicate that continuation of ICS is still adequate as maintenance treatment.	Total sample		10.2%	48.8%	40.9%		0.298
	Medical speciality	General Practitioners	15.6%	48.9%	35.6%		
		Pulmonologists	7.3%	48.8%	43.9%		
to assess factors that indicate if ICS are adequate for treatment initiation.	Total sample		11.8%	41.7%	46.5%		0.455
	Medical speciality	General Practitioners	15.6%	44.4%	40.0%		
		Pulmonologists	9.8%	40.2%	50.0%		
There is not enough evidence supporting ICS withdrawal for…<br>*(Items are displayed in order of higher frequency)*							
for patients with low/medium blood eosinophil count (100-300 cells/µl).	Total sample		20.5%	67.7%	8.7%	3.1%	0.030
	Medical speciality	General Practitioners	17.8%	68.9%	4.4%	8.9%	
		Pulmonologists	22.0%	67.1%	11.0%		
for COPD patients with co-morbidities.	Total sample		24.4%	59.8%	11.0%	4.7%	0.610
	Medical speciality	General Practitioners	24.4%	62.2%	6.7%	6.7%	
		Pulmonologists	24.4%	58.5%	13.4%	3.7%	
for patients with low blood eosinophil count (<100 cells/µl).	Total sample		65.4%	21.3%	10.2%	3.1%	0.042
	Medical speciality	General Practitioners	64.4%	20.0%	6.7%	8.9%	
		Pulmonologists	65.9%	22.0%	12.2%		
for patients with high blood eosinophil count (>300 cells/µl).	Total sample		15.0%	15.7%	64.6%	4.7%	0.002
	Medical speciality	General Practitioners	6.7%	11.1%	68.9%	13.3%	
		Pulmonologists	19.5%	18.3%	62.2%		

Supplementary Table 14. Opinion on the current use of inhaled corticosteroids. Global results and sub-analysis by country.

		Completely disagree (1-3)	Neutral (4-6)	Completely agree (7-9)	Don't know	p value
(Items are displayed in order of higher frequency)		%				
ICS are not over-prescribed in the primary care setting.	Total sample	79.5%	12.6%	7.9%		0.340
	Belgium	84.6%	11.5%	3.8%		
	Finland	42.9%	42.9%	14.3%		
	Greece	88.2%	5.9%	5.9%		
	Norway	84.0%	8.0%	8.0%		
	Portugal	79.4%	8.8%	11.8%		
	The Netherlands	72.2%	22.2%	5.6%		
There is not enough evidence on the benefits of ICS withdrawal.	Total sample	60.6%	29.1%	8.7%	1.6%	0.014
	Belgium	76.9%	15.4%	7.7%		
	Finland	28.6%	57.1%	14.3%		
	Greece	29.4%	47.1%	23.5%		
	Norway	52.0%	44.0%		4.0%	
	Portugal	79.4%	8.8%	8.8%	2.9%	
	The Netherlands	55.6%	38.9%	5.6%		
ICS are not over-prescribed in the specialty care.	Total sample	33.9%	51.2%	13.4%	1.6%	0.735
	Belgium	34.6%	53.8%	11.5%		
	Finland		85.7%	14.3%		
	Greece	35.3%	47.1%	11.8%	5.9%	
	Norway	28.0%	56.0%	16.0%		
	Portugal	41.2%	44.1%	14.7%		
	The Netherlands	38.9%	44.4%	11.1%	5.6%	
Pulmonologists feel insecure with ICS withdrawal.	Total sample	20.5%	59.1%	14.2%	6.3%	0.604
	Belgium	30.8%	42.3%	19.2%	7.7%	
	Finland	14.3%	85.7%			
	Greece	17.6%	58.8%	23.5%		
	Norway	16.0%	72.0%	8.0%	4.0%	
	Portugal	17.6%	52.9%	17.6%	11.8%	

	The Netherlands	22.2%	66.7%	5.6%	5.6%	
The health care system facilitates ICS prescription in comparison to other treatments	Total sample	54.3%	23.6%	15.7%	6.3%	0.000
	Belgium	50.0%	26.9%	23.1%		
	Finland	42.9%	57.1%			
	Greece	17.6%	41.2%	29.4%	11.8%	
	Norway	68.0%	20.0%	8.0%	4.0%	
	Portugal	73.5%	14.7%	11.8%		
	The Netherlands	44.4%	11.1%	16.7%	27.8%	
Physicians are familiar with international recommendations regarding ICS treatment restrictions.	Total sample	18.9%	54.3%	25.2%	1.6%	0.036
	Belgium	19.2%	53.8%	26.9%		
	Finland	14.3%	71.4%	14.3%		
	Greece	23.5%	70.6%	5.9%		
	Norway	36.0%	36.0%	24.0%	4.0%	
	Portugal	5.9%	70.6%	20.6%	2.9%	
	The Netherlands	16.7%	27.8%	55.6%		
Local and national guidelines are timely updated according to latest evidence.	Total sample	15.7%	29.1%	54.3%	0.8%	0.347
	Belgium	11.5%	38.5%	50.0%		
	Finland		28.6%	71.4%		
	Greece	5.9%	41.2%	52.9%		
	Norway	36.0%	16.0%	48.0%		
	Portugal	14.7%	29.4%	52.9%	2.9%	
	The Netherlands	11.1%	22.2%	66.7%		
GPs feel insecure with ICS withdrawal.		5.5%	20.5%	70.9%	3.1%	0.638
	Belgium	7.7%	15.4%	73.1%	3.8%	
	Finland		57.1%	42.9%		
	Greece	5.9%	11.8%	82.4%		
	Norway	4.0%	20.0%	72.0%	4.0%	
	Portugal	8.8%	14.7%	73.5%	2.9%	
	The Netherlands		33.3%	61.1%	5.6%	
There is enough time in daily clinical practice to… *(Items are displayed in order of higher frequency)*						

to assess factors that indicate that continuation of ICS is still adequate as maintenance treatment.	Total sample	10.2%	48.8%	40.9%		0.516
	Belgium	3.8%	53.8%	42.3%		
	Finland	28.6%	28.6%	42.9%		
	Greece	5.9%	64.7%	29.4%		
	Norway	20.0%	40.0%	40.0%		
	Portugal	8.8%	47.1%	44.1%		
	The Netherlands	5.6%	50.0%	44.4%		
to assess factors that indicate if ICS are adequate for treatment initiation.	Total sample	11.8%	41.7%	46.5%		0.983
	Belgium	7.7%	38.5%	53.8%		
	Finland	14.3%	42.9%	42.9%		
	Greece	11.8%	47.1%	41.2%		
	Norway	16.0%	48.0%	36.0%		
	Portugal	11.8%	35.3%	52.9%		
	The Netherlands	11.1%	44.4%	44.4%		
There is not enough evidence supporting ICS withdrawal for… *(Items are displayed in order of higher frequency)*						
for patients with low/medium blood eosinophil count (100-300 cells/µl).	Total sample	20.5%	67.7%	8.7%	3.1%	0.176
	Belgium	11.5%	69.2%	11.5%	7.7%	
	Finland	14.3%	71.4%	14.3%		
	Greece	41.2%	41.2%	17.6%		
	Norway	32.0%	64.0%	4.0%		
	Portugal	20.6%	70.6%	5.9%	2.9%	
	The Netherlands		88.9%	5.6%	5.6%	
for COPD patients with co-morbidities.	Total sample	24.4%	59.8%	11.0%	4.7%	0.412
	Belgium	19.2%	61.5%	15.4%	3.8%	
	Finland	28.6%	42.9%	14.3%	14.3%	
	Greece	23.5%	70.6%	5.9%		
	Norway	40.0%	48.0%	8.0%	4.0%	
	Portugal	20.6%	67.6%	11.8%		
	The Netherlands	16.7%	55.6%	11.1%	16.7%	
	Total sample	65.4%	21.3%	10.2%	3.1%	0.445

for patients with low blood eosinophil count (<100 cells/µl).	Belgium	57.7%	19.2%	15.4%	7.7%	
	Finland	42.9%	42.9%	14.3%		
	Greece	76.5%	17.6%	5.9%		
	Norway	60.0%	24.0%	16.0%		
	Portugal	82.4%	11.8%	2.9%	2.9%	
	The Netherlands	50.0%	33.3%	11.1%	5.6%	
for patients with high blood eosinophil count (>300 cells/µl).	Total sample	15.0%	15.7%	64.6%	4.7%	0.103
	Belgium	11.5%	19.2%	57.7%	11.5%	
	Finland		14.3%	85.7%		
	Greece	23.5%	23.5%	52.9%		
	Norway	28.0%	24.0%	48.0%		
	Portugal	5.9%	5.9%	85.3%	2.9%	
	The Netherlands	16.7%	11.1%	61.1%	11.1%	

Supplementary Table 15. Participants experience on ICS withdrawal in COPD patients. Global results and sub-analysis by country.

			Results by country						
		Total sample	Belgium	Finland	Greece	Norway	Portugal	The Netherlands	p value
**Patients' distribution per HCP, in the last year (n=127)**	**N of respondents**	**127**	**26**	**7**	**17**	**25**	**34**	**18**	
Total COPD patients seen in the last year, mean		300	415	263	203	215	187	572	>0.0001
ICS-treated patients, N (%)		117 (37.1%)	139 (34.6)	126 (45.7)	73 (37.9)	89 (40.2)	65 (29.8)	257 (46.2)	0.030
ICS-treated patients eligible for ICS withdrawal, %		22.0%	26.0%	17.9%	20.8%	17.8%	22.9%	23.3%	0.760
**Have you ever withdrawn ICS treatment from any COPD patient? Yes, n (%)**		**119 (93.7)**	**25 (96%)**	**7 (100%)**	**16 (94%)**	**24 (96%)**	**29 (85%)**	**18 (100%)**	**0.283**
Patients withdrawn from ICS treatment, %		17.2%	21.8%	16.7%	23.4%	10.5%	17.8%	13.3%	0.108
Patients that refused ICS withdrawal, %		8.6%	14.6%	5.9%	6.9%	7.0%	4.1%	12.4%	0.219
**Patients' distribution per HCP (only those who have withdrawn an ICS treatment in the last year)**	**N of respondents**	**111**	**25**	**7**	**15**	**21**	**25**	**18**	
Reason for ICS withdrawal									
No longer indication for ICS, %		49.5%	59.8%	32.9%	52.7%	38.1%	46.0%	56.9%	0.091
Lack of response to ICS, %		27.1%	17.4%	40.0%	35.7%	33.6%	26.5%	21.4%	0.154
Pneumonia, %		11.8%	12.8%	17.9%	2.0%	13.3%	17.4%	6.7%	0.185
Fear of adverse events to ICS, %		11.2%	8.8%	9.3%	9.7%	14.0%	10.0%	15.0%	0.697
Other, %		0.5%	1.2%	0.0%	0.0%	1.0%	0.0%	0.0%	0.546
Outcome of ICS withdrawal									
Remained with no exacerbations within 6 months after ICS withdrawal, %		75.0%	73.0%	72.0%	78.0%	66.0%	76.0%	86.0%	0.321
Needed ICS treatment reintroduction within 6 months after ICS withdrawal, %		16.0%	18.0%	21.0%	14.0%	17.0%	13.0%	17.0%	0.693
**Reasons for ICS re-introduction**	**N of respondents**	**81**	**22**	**6**	**12**	**11**	**19**	**11**	
Patient preference, %		16.0%	16.0%	4.0%	8.0%	25.0%	20.0%	15.0%	0.403
Increased symptoms, %		37.0%	35.0%	56.0%	41.0%	39.0%	28.0%	37.0%	0.354
Exacerbation, %		42.0%	48.0%	38.0%	38.0%	25.0%	51.0%	43.0%	0.221
Other, %		5.0%	1.0%	2.0%	13.0%	12.0%	2.0%	5.0%	0.271

Supplementary Table 16. Participant's opinion on how ICS therapy should be withdrawn from their COPD patients. Global results and sub-analysis by specialty.

		Results by speciality		
	Total sample (N=127)	General Practitioners (N=45)	Pulmonologists (N=82)	p value
In your decision to withdraw ICS therapy, which parameter do you rely on most? *(Multiple choice option)*				0.385
Frequency of exacerbations	35.4%	42.2%	31.7%	
Level of blood eosinophil counts	1.6%	2.2%	1.2%	
Always both in combination	48.8%	44.4%	51.2%	
None of these two	3.9%		6.1%	
Other factors	10.2%	11.1%	9.8%	
For what reasons do you think ICS-treated patients can be eligible for ICS withdrawal? *(Multiple choice option)*				0.274
Patients with pneumonia	68.5%	73.3%	65.9%	0.385
No longer indication for ICS	87.4%	93.3%	84.1%	0.136
Lack of response to ICS	88.2%	86.7%	89.0%	0.694
Patients without asthma	39.4%	46.7%	35.4%	0.212
Patients with diabetes	10.2%	11.1%	9.8%	0.810
Patients with osteoporosis/osteopenia	17.3%	17.8%	17.1%	0.920
Patients with cardiovascular disease	1.6%	4.4%		0.054
Patients with ICS-related side effects	81.1%	86.7%	78.0%	0.235
Patients should never be withdrawn from ICS treatment	0.0%	0.0%	0.0%	-
How confident you are in the following situations:				
Withdrawal in case of no longer indication for ICS. Confident, %	87.4%	91.1%	85.4%	0.312
ICS withdrawal in case of lack of response to ICS. Confident, %	77.2%	82.2%	74.4%	0.388
ICS withdrawal in case of pneumonia. Confident.	63.0%	77.8%	54.9%	0.023
In your personal opinion, how should ICS be withdrawn in patients on LABA/ICS therapy? *(Multiple choice option)*				0.001
Gradual ICS dose reduction without adding any other treatment in all patients	0.8%	0.0%	1.2%	0.457
Gradual ICS dose reduction without adding any other treatment in patients on high dose ICS	3.9%	0.0%	6.1%	0.091
Abrupt ICS withdrawal without adding any other treatment	7.9%	6.7%	8.5%	0.708
Gradual dose reduction and LAMA/LABA treatment introduction at the same time	55.1%	68.9%	47.6%	0.021
Gradual dose reduction and LAMA/LABA treatment once ICS is completely withdrawn	0.8%	2.2%	0.0%	0.175
Abrupt ICS withdrawal and LAMA/LABA treatment once ICS is completely withdrawn	59.1%	37.8%	70.7%	0.000
ICS should not be withdrawn from patients on LABA/ICS	0.0%	2.2%	1.2%	-
Other	1.6%	0.0%	3.2%	0.664
In your personal opinion, how should ICS be withdrawn in patients on triple therapy (LAMA/LABA/ICS)? *(Multiple choice option)*				<0.0001
Gradual ICS dose reduction maintaining LAMA/LABA combination	28.3%	55.6%	13.4%	<0.0001

Abrupt ICS withdrawal maintaining LAMA/LABA combination	74.0%	46.7%	89.0%	<0.0001
ICS should not be withdrawn from patients on LABA/ICS	0.0%	0.0%	0.0%	-
Other	0.8%	2.2%	0.0%	0.175
**In your personal opinion, what drives the choice of LAMA/LABA combination when withdrawing ICS in patients who were on triple therapy?** *(Multiple choice option)*				0.116
Same device	89.8%	97.8%	85.4%	0.027
Same LAMA	6.3%	8.9%	4.9%	0.374
Same LABA	6.3%	8.9%	4.9%	0.374
Switch to a potentially more effective LAMA	9.4%	6.7%	11.0%	0.427
Switch to a potentially more effective LABA	5.5%	2.2%	7.3%	0.229
Other	2.4%		3.7%	0.194
**In your personal opinion, after ICS withdrawal, how should patients with COPD be monitored?** *(Multiple choice option)*				0.001
No specific follow-up	0.0%			-
Planned follow-up visit / call 1 month after ICS withdrawal	73.2%	86.7%	65.9%	0.011
Planned visit for spirometry	33.9%	24.4%	39.0%	0.097
Patients should have the possibility to communicate with me or another healthcare professional in case of questions	56.7%	73.3%	47.6%	0.005
Patients should have the possibility to call me or another healthcare professional in case of complaints	32.3%	42.2%	26.8%	0.076
Other	0.8%		1.2%	0.457
**Please indicate the reasons that could indicate the need for reintroducing ICS** *(Multiple choice option)*				0.026
Worsening of COPD symptoms (such as breathlessness)	68.5%	75.6%	64.6%	0.205
Exacerbations after ICS withdrawal	95.3%	88.9%	98.8%	0.012
Persistent adverse events after ICS withdrawal	5.5%	11.1%	2.4%	0.041
Significant worsening of spirometry	66.1%	62.2%	68.3%	0.489
Patient preference	7.9%	6.7%	8.5%	0.708
Others	0.0%	0.0%	0.0%	-
**What should be done in such cases?** *(Multiple choice option)*				0.034
Back to the ICS treatment at the same doses than before	35.4%	22.2%	42.7%	
Back to the ICS treatment at lower doses than before	63.8%	75.6%	57.3%	
Keep the ICS withdrawal	0.8%	2.2%	0.0%	
Other	0.0%	0.0%	0.0%	-

Supplementary Table 17. Participant's opinion on how ICS therapy should be withdrawn from their COPD patients. Global results and sub-analysis by country.

		**Results by country**							
	**Total sample (N=127)**	**Belgium (N=26)**	**Finland (N=7)**	**Greece (N=17)**	**Norway (N=25)**	**Portugal (N=34)**	**The Netherlands (N=18)**	**p value**
**In your decision to withdraw ICS therapy, which parameter do you rely on most?**								0.137
Frequency of exacerbations	35.4%	38.5%	0.0%	23.5%	40.0%	32.4%	55.6%	
Level of blood eosinophil count	1.6%	3.8%	0.0%			2.9%		
Always both in combination	48.8%	42.3%	85.7%	41.2%	48.0%	61.8%	27.8%	
None of these two	3.9%	3.8%	14.3%	17.6%				
Other factors	10.2%	11.5%		17.6%	12.0%	2.9%	16.7%	
**For what reasons do you think ICS-treated patients can be eligible for ICS withdrawal?**								0.054
Patients with pneumonia	68.5%	69.0%	71.0%	53.0%	64.0%	85.0%	56.0%	0.155
No longer indication for ICS	87.4%	88.0%	100.0%	65.0%	96.0%	85.0%	94.0%	0.040
Lack of response to ICS	88.2%	88.0%	86.0%	88.0%	92.0%	94.0%	72.0%	0.312
Patients without asthma	39.4%	23.0%	29.0%	29.0%	56.0%	29.0%	72.0%	0.006
Patients with diabetes	10.2%	15.0%	14.0%	12.0%	4.0%	12.0%	6.0%	0.778
Patients with osteoporosis/osteopenia	17.3%	23.0%	29.0%	12.0%	16.0%	21.0%	6.0%	0.607
Patients with cardiovascular disease	1.6%					3.0%	6.0%	0.631
Patients with ICS-related side effects	81.1%	92.0%	86.0%	76.0%	76.0%	82.0%	72.0%	0.571
Patients should never be withdrawn from ICS treatment	0.0%	0.0%	0.0%	0.0%	0.0%	0.0%	0.0%	-

**How confident you are in the following situations:**								
Withdrawal in case of no longer indication for ICS. Confident, %	87.4%	88.5%	57.1%	76.5%	92.0%	91.2%	94.4%	0.115
ICS withdrawal in case of lack of response to ICS. Confident, %	77.2%	69.2%	100.0%	82.4%	76.0%	82.4%	66.7%	0.885
ICS withdrawal in case of pneumonia. Confident, %	63.0%	61.5%	57.1%	52.9%	52.0%	85.3%	50.0%	0.002
**In your personal opinion, how should ICS be withdrawn in patients on LABA/ICS therapy? %**								0.033
Gradual ICS dose reduction without adding any other treatment in all patients	0.8%	0.0%	0.0%	5.9%	0.0%	0.0%	0.0%	0.259
Gradual ICS dose reduction without adding any other treatment in patients on high dose ICS	3.9%	3.8%	28.6%	5.9%	4.0%	0.0%	0.0%	0.019
Abrupt ICS withdrawal without adding any other treatment	7.9%	3.8%	0.0%	5.9%	12.0%	2.9%	22.2%	0.150
Gradual dose reduction and LAMA/LABA treatment introduction at the same time	55.1%	53.8%	71.4%	47.1%	48.0%	67.6%	44.4%	0.452
Gradual dose reduction and LAMA/LABA treatment once ICS is completely withdrawn	0.8%	0.0%	0.0%	0.0%	0.0%	2.9%	0.0%	0.737
Abrupt ICS withdrawal and LAMA/LABA treatment once ICS is completely withdrawn	59.1%	65.4%	71.4%	70.6%	60.0%	47.1%	55.6%	0.559
ICS should not be withdrawn from patients on LABA/ICS	0.0%	0.0%	0.0%	0.0%	0.0%	0.0%	0.0%	-
Other	1.6%	0.0%	0.0%	0.0%	0.0%	0.0%	11.1%	0.031
**In your personal opinion, how should ICS be withdrawn in patients on triple therapy (LAMA/LABA/ICS)?**								0.094
Gradual ICS dose reduction maintaining LAMA/LABA combination	28.3%	23.1%	28.6%	23.5%	28.0%	47.1%	5.6%	0.051
Abrupt ICS withdrawal maintaining LAMA/LABA combination	74.0%	76.9%	85.7%	76.5%	72.0%	61.8%	88.9%	0.601
ICS should not be withdrawn from patients on LABA/ICS	0.0%	0.0%	0.0%	0.0%	0.0%	0.0%	0.0%	0.000
Other	0.8%	0.0%	0.0%	0.0%	0.0%	0.0%	5.6%	0.296

**In your personal opinion, what drives the choice of LAMA/LABA combination when withdrawing ICS in patients who were on triple therapy?**								0.000
Same device	89.8%	92.3%	100.0%	52.9%	96.0%	97.1%	94.4%	0.000
Same LAMA	6.3%	3.8%	0.0%	5.9%	4.0%	8.8%	11.1%	0.844
Same LABA	6.3%	3.8%	0.0%	5.9%	4.0%	8.8%	11.1%	0.844
Switch to a potentially more effective LAMA	9.4%	11.5%	0.0%	23.5%	12.0%	5.9%	0.0%	0.194
Switch to a potentially more effective LABA	5.5%	0.0%	0.0%	23.5%	4.0%	2.9%	5.6%	0.023
Other	2.4%	0.0%	0.0%	11.8%	0.0%	2.9%	0.0%	0.135
**In your personal opinion, after ICS withdrawal, how should patients with COPD be monitored?**								0.069
No specific follow-up	0.0%	0.0%	0.0%	0.0%	0.0%	0.0%	0.0%	-
Planned follow-up visit / call 1 month after ICS withdrawal	73.2%	53.8%	85.7%	76.5%	76.0%	82.4%	72.2%	0.208
Planned visit for spirometry	33.9%	30.8%	28.6%	64.7%	40.0%	29.4%	11.1%	0.031
Patients should have the possibility to communicate with me or another healthcare professional in case of questions	56.7%	53.8%	57.1%	47.1%	48.0%	70.6%	55.6%	0.524
Patients should have the possibility to call me or another healthcare professional in case of complaints	32.3%	50.0%	0.0%	23.5%	24.0%	35.3%	33.3%	0.126
Other	0.8%	3.8%	0.0%	0.0%	0.0%	0.0%	0.0%	0.562
**Please indicate the reasons that could indicate the need for reintroducing ICS**								0.027
Worsening of COPD symptoms (such as breathlessness)	68.5%	76.9%	85.7%	70.6%	80.0%	58.8%	50.0%	0.173
Exacerbations after ICS withdrawal	95.3%	92.3%	100.0%	94.1%	96.0%	97.1%	94.4%	0.945
Persistent adverse events after ICS withdrawal	5.5%	3.8%	28.6%	0.0%	4.0%	8.8%	0.0%	0.071
Significant worsening of spirometry	66.1%	61.5%	71.4%	82.4%	80.0%	61.8%	44.4%	0.129

Patient preference	7.9%	3.8%	14.3%	0.0%	16.0%	0.0%	22.2%	0.010
Others	0.0%	0.0%	0.0%	0.0%	0.0%	0.0%	0.0%	-
**What should be done in such cases?**								0.094
Back to the ICS treatment at the same doses than before	35.4%	46.2%	14.3%	58.8%	40.0%	23.5%	22.2%	
Back to the ICS treatment at lower doses than before	63.8%	53.8%	85.7%	41.2%	60.0%	76.5%	72.2%	
Keep the ICS withdrawal	0.8%	0.0%	0.0%	0.0%	0.0%	0.0%	5.6%	
Other	0.0%	0.0%	0.0%	0.0%	0.0%	0.0%	0.0%	-

Supplementary Table 18. Potential impact of COPD treatment optimization. Global results and sub-analysis by medical speciality.

			Completely disagree (1-3)	Neutral (4-6)	Completely agree (7-9)	Don't know	p value
			%				
a. Reducing the use of non-indicated ICS in COPD patients could result in…. *(Items are displayed in order of higher frequency)*							
Worsening of quality of life	Total sample		65.4%	29.1%	5.5%		0.599
	Medical speciality	General Practitioners	71.1%	24.4%	4.4%		
		Pulmonologists	62.2%	31.7%	6.1%		
Improvement of treatment adherence			9.4%	69.3%	20.5%	0.8%	0.805
	Medical speciality	General Practitioners	8.9%	73.3%	17.8%		
		Pulmonologists	9.8%	67.1%	22.0%	1.2%	
A reduction of adverse events such as infections			2.4%	18.9%	76.4%	2.4%	0.467
	Medical speciality	General Practitioners	4.4%	13.3%	80.0%	2.2%	
		Pulmonologists	1.2%	22.0%	74.4%	2.4%	
A better outcome of co-existing diseases			0.8%	22.0%	76.4%	0.8%	0.773
	Medical speciality	General Practitioners		22.2%	77.8%		
		Pulmonologists	1.2%	22.0%	75.6%	1.2%	
A reduction of bacterial exacerbations				20.5%	78.0%	1.6%	0.317
	Medical speciality	General Practitioners		13.3%	84.4%	2.2%	
		Pulmonologists		24.4%	74.4%	1.2%	
b. Increasing the use of LABA/LAMA in patients for whom LABA/ICS is not indicated could result in…. *(Items are displayed in order of higher frequency)*							
Increased adverse events			72.4%	18.9%	7.9%	0.8%	0.314
	Medical speciality	General Practitioners	77.8%	15.6%	4.4%	2.2%	

		Pulmonologists	69.5%	20.7%	9.8%		
Reduced exacerbations			3.1%	26.8%	70.1%		0.602
	Medical speciality	General Practitioners	4.4%	22.2%	73.3%		
		Pulmonologists	2.4%	29.3%	68.3%		
A better control of COPD			3.1%	13.4%	82.7%	0.8%	0.362
	Medical speciality	General Practitioners	2.2%	8.9%	86.7%	2.2%	
		Pulmonologists	3.7%	15.9%	80.5%		
Increased clinical response to treatment			2.4%	15.0%	82.7%		0.925
	Medical speciality	General Practitioners	2.2%	13.3%	84.4%		
		Pulmonologists	2.4%	15.9%	81.7%		
c. In patients with further exacerbations with high eosinophil count, stepping up the inhaled treatment to LABA/LAMA/ICS (triple therapy) from LABA/LAMA may….<br>*(Items are displayed in order of higher frequency)*							
Increase adverse events (such as infections)			12.6%	63.0%	22.8%	1.6%	0.109
	Medical speciality	General Practitioners	6.7%	62.2%	26.7%	4.4%	
		Pulmonologists	15.9%	63.4%	20.7%		
Improve lung function			2.4%	27.6%	68.5%	1.6%	0.087
	Medical speciality	General Practitioners	2.2%	17.8%	75.6%	4.4%	
		Pulmonologists	2.4%	32.9%	64.6%		
Increase clinical response to treatment			2.4%	13.4%	81.9%	2.4%	0.028
	Medical speciality	General Practitioners	4.4%	6.7%	82.2%	6.7%	
		Pulmonologists	1.2%	17.1%	81.7%		
Prevent exacerbations			2.4%	12.6%	83.5%	1.6%	0.286
	Medical speciality	General Practitioners	2.2%	11.1%	82.2%	4.4%	
		Pulmonologists	2.4%	13.4%	84.1%		
Control COPD better			2.4%	11.0%	84.3%	2.4%	0.122

	Medical speciality	General Practitioners	2.2%	8.9%	82.2%	6.7%	
		Pulmonologists	2.4%	12.2%	85.4%		

Supplementary Table 19. Potential impact of COPD treatment optimization. Global results and sub-analysis by country.

		Completely disagree (1-3)	Neutral (4-6)	Completely agree (7-9)	Don't know	p value
		%				
a. Reducing the use of non-indicated ICS in COPD patients could result in…. *(Items are displayed in order of higher frequency)*						
Worsening of quality of life	Total sample	65%	29%	6%		0.042
	Belgium	69%	23%	8%		
	Finland	57%	43%			
	Greece	41%	59%			
	Norway	72%	24%	4%		
	Portugal	76%	12%	12%		
	The Netherlands	56%	44%			
Improvement of treatment adherence	Total sample	9%	69%	20%	0.8%	0.531
	Belgium	19%	54%	27%		
	Finland		71%	29%		
	Greece	6%	71%	24%		
	Norway	4%	80%	16%		
	Portugal	12%	74%	15%		
	The Netherlands	6%	67%	22%	5.6%	
A reduction of adverse events such as infections	Total sample	2%	19%	76%	2.4%	0.084
	Belgium	4%	38%	54%	3.8%	
	Finland		14%	71%	14.3%	
	Greece		12%	88%		
	Norway		24%	72%	4.0%	
	Portugal	3%	3%	94%		
	The Netherlands	6%	22%	72%		
A better outcome of co-existing diseases	Total sample	1%	22%	76%	0.8%	0.565
	Belgium	4%	35%	62%		
	Finland		14%	86%		
	Greece		24%	76%		
	Norway		20%	76%	4.0%	
	Portugal		12%	88%		
	The Netherlands		28%	72%		
A reduction of bacterial exacerbations	Total sample		20%	78%	1.6%	0.127
	Belgium		15%	81%	3.8%	
	Finland		43%	57%		

	Greece		35%	65%		
	Norway		24%	76%		
	Portugal		3%	94%	2.9%	
	The Netherlands		33%	67%		
b. Increasing the use of LABA/LAMA in patients for whom LABA/ICS is not indicated could result in….<br><br>*(Items are displayed in order of higher frequency)*						
Increased adverse events	Total sample	72%	19%	8%	0.8%	0.006
	Belgium	65%	23%	12%		
	Finland	43%	57%			
	Greece	82%	18%			
	Norway	80%	16%	4%		
	Portugal	91%	3%	6%		
	The Netherlands	39%	33%	22%	5.6%	
Reduced exacerbations	Total sample	3%	27%	70%		0.102
	Belgium		23%	77%		
	Finland		14%	86%		
	Greece	6%	41%	53%		
	Norway	8%	32%	60%		
	Portugal		12%	88%		
	The Netherlands	6%	44%	50%		
A better control of COPD	Total sample	3%	13%	83%	0.8%	0.171
	Belgium	4%	23%	73%		
	Finland			100%		
	Greece		12%	88%		
	Norway	8%	24%	68%		
	Portugal		3%	97%		
	The Netherlands	6%	11%	78%	5.6%	
Increased clinical response to treatment	Total sample	2%	15%	83%		0.216
	Belgium	4%	27%	69%		
	Finland			100%		
	Greece		12%	88%		
	Norway	4%	24%	72%		
	Portugal		3%	97%		
	The Netherlands	6%	17%	78%		
c. In patients with further exacerbations with high eosinophil count, stepping up the inhaled treatment to LABA/LAMA/ICS (triple therapy) from LABA/LAMA may….<br><br>*(Items are displayed in order of higher frequency)*						
Increase adverse events (such as infections)	Total sample	13%	63%	23%	1.6%	0.521
	Belgium	15%	50%	31%	3.8%	
	Finland	14%	71%	14%		
	Greece	18%	71%	12%		

	Norway	8%	68%	24%		
	Portugal	18%	68%	15%		
	The Netherlands		56%	39%	5.6%	
Improve lung function	Total sample	2%	28%	69%	1.6%	0.082
	Belgium	4%	31%	62%	3.8%	
	Finland		14%	86%		
	Greece		35%	65%		
	Norway	4%	48%	48%		
	Portugal		6%	94%		
	The Netherlands	6%	33%	56%	5.6%	
Increase clinical response to treatment	Total sample	2%	13%	82%	2.4%	0.073
	Belgium	4%	27%	65%	3.8%	
	Finland			86%	14.3%	
	Greece		18%	82%		
	Norway	8%	12%	80%		
	Portugal			97%	2.9%	
	The Netherlands		22%	78%		
Prevent exacerbations	Total sample	2%	13%	83%	1.6%	0.220
	Belgium	4%	15%	81%		
	Finland			86%	14.3%	
	Greece		24%	76%		
	Norway	8%	12%	80%		
	Portugal		6%	91%	2.9%	
	The Netherlands		17%	83%		
Control COPD better	Total sample	2%	11%	84%	2.4%	0.170
	Belgium	4%	15%	77%	3.8%	
	Finland			86%	14.3%	
	Greece		12%	88%		
	Norway	8%	16%	76%		
	Portugal			97%	2.9%	
	The Netherlands		22%	78%		

Supplementary Table 20. Resources to optimize the COPD patient management and treatment. Global results and sub-analysis by medical speciality.

(Items are displayed in order of higher frequency)			Completely disagree (1-3)	Neutral (4-6)	Completely agree (7-9)	Don't know	p value
			%				
A guidance tool on how to withdraw ICS in each type of patient.	Total sample		3.9%	28.3%	67.7%		0.764
	Medical speciality	General Practitioners	4.4%	24.4%	71.1%		
		Pulmonologists	3.7%	30.5%	65.9%		
Faster updates of local guidelines.	Total sample		3.9%	26.8%	69.3%		0.680
	Medical speciality	General Practitioners	4.4%	31.1%	64.4%		
		Pulmonologists	3.7%	24.4%	72.0%		
Decrease waiting time regarding referral from GPs to specialists	Total sample		3.1%	26.8%	69.3%	0.8%	0.144
	Medical speciality	General Practitioners	4.4%	37.8%	57.8%		
		Pulmonologists	2.4%	20.7%	75.6%	1.2%	
Collaboration/ support to GPs from pulmonologists.	Total sample		7.9%	20.5%	71.7%		0.454
	Medical speciality	General Practitioners	4.4%	24.4%	71.1%		
		Pulmonologists	9.8%	18.3%	72.0%		
Improvement of doctor-patient communication that helps the physician discuss patient preference related to treatment (shared decision)	Total sample		5.5%	21.3%	73.2%		0.691
	Medical speciality	General Practitioners	4.4%	17.8%	77.8%		
		Pulmonologists	6.1%	23.2%	70.7%		
Have access to a patient support program that helps them to Identify their symptoms and report them to the HCP.	Total sample		3.9%	19.7%	74.0%	2.4%	0.129
	Medical speciality	General Practitioners	2.2%	28.9%	64.4%	4.4%	
		Pulmonologists	4.9%	14.6%	79.3%	1.2%	
Have access to a patient support program that helps them to receive more information about their illness.	Total sample		1.6%	23.6%	74.8%		0.893
	Medical speciality	General Practitioners	2.2%	24.4%	73.3%		
		Pulmonologists	1.2%	23.2%	75.6%		

Optimize the referral from GPs to specialists	Total sample		4.7%	19.7%	75.6%		0.009
	Medical speciality	General Practitioners	8.9%	31.1%	60.0%		
		Pulmonologists	2.4%	13.4%	84.1%		
Integrate a tool to assess comorbidities and drug interactions in a medical software to trigger alerts.	Total sample		4.7%	18.9%	76.4%		0.580
	Medical speciality	General Practitioners	2.2%	17.8%	80.0%		
		Pulmonologists	6.1%	19.5%	74.4%		
Increase patient consultation time.	Total sample		4.7%	16.5%	77.2%	1.6%	0.048
	Medical speciality	General Practitioners	2.2%	6.7%	91.1%		
		Pulmonologists	6.1%	22.0%	69.5%	2.4%	
Specifically train physicians on the management of COPD patients.	Total sample		5.5%	15.7%	78.0%	0.8%	0.358
	Medical speciality	General Practitioners	2.2%	15.6%	80.0%	2.2%	
		Pulmonologists	7.3%	15.9%	76.8%		
Have access to a patient support program that helps them to cope with their illness.	Total sample		2.4%	18.9%	78.7%		0.253
	Medical speciality	General Practitioners	2.2%	26.7%	71.1%		
		Pulmonologists	2.4%	14.6%	82.9%		
Align the different COPD-guidelines.	Total sample		3.9%	17.3%	78.7%		0.912
	Medical speciality	General Practitioners	4.4%	15.6%	80.0%		
		Pulmonologists	3.7%	18.3%	78.0%		
Have access to a validated tool that helps HCPs identify patients who would benefit from ICS vs. those in whom it may be inappropriate.	Total sample		3.9%	15.0%	80.3%	0.8%	0.501
	Medical speciality	General Practitioners	2.2%	15.6%	80.0%	2.2%	
		Pulmonologists	4.9%	14.6%	80.5%		
Incorporate recommendations for the treatment and management of the multi-morbid COPD patient in the current guidelines.	Total sample		2.4%	15.0%	81.9%	0.8%	0.276
	Medical speciality	General Practitioners		17.8%	80.0%	2.2%	
		Pulmonologists	3.7%	13.4%	82.9%		

Have access to a patient support program that helps them to stop smoking.	Total sample		2.4%	12.6%	85.0%		0.000
	Medical speciality	General Practitioners		28.9%	71.1%		
		Pulmonologists	3.7%	3.7%	92.7%		

Supplementary Table 21. Resources to optimize the COPD patient management and treatment. Global results and sub-analysis by country.

(Items are displayed in order of higher frequency)		Completely disagree (1-3)	Neutral (4-6)	Completely agree (7-9)	Don't know	p value
		%				
A guidance tool on how to withdraw ICS in each type of patient.	Total sample	4%	28%	68%		0.227
	Belgium	8%	35%	58%		
	Finland		29%	71%		
	Greece	6%	29%	65%		
	Norway	8%	40%	52%		
	Portugal		12%	88%		
	The Netherlands		33%	67%		
Faster updates of local guidelines.		4%	27%	69%		0.005
	Belgium	4%	35%	62%		
	Finland		29%	71%		
	Greece	6%	24%	71%		
	Norway	12%	52%	36%		
	Portugal		9%	91%		
	The Netherlands		17%	83%		
Decrease waiting time regarding referral from GPs to specialists		3%	27%	69%	0.8%	0.073
	Belgium	4%	31%	65%		
	Finland		29%	71%		
	Greece			94%	5.9%	
	Norway	8%	36%	56%		
	Portugal	3%	18%	79%		
	The Netherlands		50%	50%		
Collaboration/ support to GPs from pulmonologists.		8%	20%	72%		0.000
	Belgium	8%	15%	77%		
	Finland		29%	71%		
	Greece	35%	29%	35%		
	Norway	4%	40%	56%		
	Portugal	3%	12%	85%		
	The Netherlands		6%	94%		
Improvement of doctor-patient communication that helps the physician discuss patient		6%	21%	73%		0.196
	Belgium	8%	35%	58%		
	Finland		29%	71%		

preference related to treatment (shared decision)	Greece	12%	12%	76%		
	Norway	12%	20%	68%		
	Portugal		12%	88%		
	The Netherlands		28%	72%		
Have access to a patient support program that helps them to Identify their symptoms and report them to the HCP.		4%	20%	74%	2.4%	0.031
	Belgium	12%	23%	65%		
	Finland		43%	57%		
	Greece		6%	94%		
	Norway	4%	28%	56%	12.0%	
	Portugal	3%	12%	85%		
	The Netherlands		22%	78%		
Have access to a patient support program that helps them to receive more information about their illness.		2%	24%	75%		0.380
	Belgium		31%	69%		
	Finland		29%	71%		
	Greece	6%	24%	71%		
	Norway	4%	32%	64%		
	Portugal		9%	91%		
	The Netherlands		28%	72%		
Optimize the referral from GPs to specialists		5%	20%	76%		0.494
	Belgium	4%	27%	69%		
	Finland		43%	57%		
	Greece	6%		94%		
	Norway	4%	28%	68%		
	Portugal	6%	15%	79%		
	The Netherlands	6%	17%	78%		
Integrate a tool to assess comorbidities and drug interactions in a medical software to trigger alerts.		5%	19%	76%		0.583
	Belgium	8%	19%	73%		
	Finland		43%	57%		
	Greece	6%	24%	71%		
	Norway	8%	24%	68%		
	Portugal	3%	9%	88%		
	The Netherlands		17%	83%		
Increase patient consultation time.		5%	17%	77%	1.6%	0.038
	Belgium	12%	19%	69%		
	Finland		14%	86%		
	Greece		6%	94%		
	Norway	4%	40%	52%	4.0%	

	Portugal	6%	3%	91%		
	The Netherlands		17%	78%	5.6%	
Specifically train physicians on the management of COPD patients.		6%	16%	78%	0.8%	0.268
	Belgium	15%	23%	62%		
	Finland		14%	86%		
	Greece		18%	82%		
	Norway	4%	24%	72%		
	Portugal		6%	91%	2.9%	
	The Netherlands	11%	11%	78%		
Have access to a patient support program that helps them to cope with their illness.		2%	19%	79%		0.736
	Belgium	8%	27%	65%		
	Finland		14%	86%		
	Greece		18%	82%		
	Norway		20%	80%		
	Portugal	3%	15%	82%		
	The Netherlands		17%	83%		
Align the different COPD-guidelines.		4%	17%	79%		0.003
	Belgium	4%	27%	69%		
	Finland			100%		
	Greece	6%	24%	71%		
	Norway	12%	36%	52%		
	Portugal			100%		
	The Netherlands		11%	89%		
Have access to a validated tool that helps HCPs identify patients who would benefit from ICS vs. those in whom it may be inappropriate.		4%	15%	80%	0.8%	0.039
	Belgium	4%	27%	69%		
	Finland		14%	71%	14.3%	
	Greece		18%	82%		
	Norway	4%	8%	88%		
	Portugal	3%	9%	88%		
	The Netherlands	11%	17%	72%		
Incorporate recommendations for the treatment and management of the multi-morbid COPD patient in the current guidelines.		2%	15%	82%	0.8%	0.541
	Belgium	8%	19%	69%	3.8%	
	Finland			100%		
	Greece		12%	88%		
	Norway	4%	24%	72%		
	Portugal		12%	88%		

	The Netherlands		11%	89%		
Have access to a patient support program that helps them to stop smoking.		2%	13%	85%		0.683
	Belgium	8%	15%	77%		
	Finland		14%	86%		
	Greece		6%	94%		
	Norway	4%	16%	80%		
	Portugal		9%	91%		
	The Netherlands		17%	83%		

Supplementary Table 22. Economic impact of COPD treatment. Global results and sub-analysis by medical speciality.

			Completely disagree (1-3)	Neutral (4-6)	Completely agree (7-9)	Don't know	p value
(Items are displayed in order of higher frequency)			%				
Medicines represent the most significant source of costs in management of COPD patients (which also includes visits to healthcare professionals, exacerbations, pneumonia).	Total sample		61.4%	21.3%	16.5%	0.8%	0.199
	Medical speciality	General Practitioners	73.3%	13.3%	13.3%		
		Pulmonologists	54.9%	25.6%	18.3%	1.2%	
At same perceived clinical added value, I prescribe the cheapest product.			16.5%	28.3%	55.1%		0.104
	Medical speciality	General Practitioners	8.9%	24.4%	66.7%		
		Pulmonologists	20.7%	30.5%	48.8%		
I am sensitive to information on the financial implications of COPD management on the healthcare expenditure.			14.2%	24.4%	61.4%		0.227
	Medical speciality	General Practitioners	15.6%	15.6%	68.9%		
		Pulmonologists	13.4%	29.3%	57.3%		
Pneumonia represents a financial burden for healthcare expenditures.			2.4%	11.8%	85.0%	0.8%	0.758
	Medical speciality	General Practitioners	2.2%	8.9%	88.9%		
		Pulmonologists	2.4%	13.4%	82.9%	1.2%	
Exacerbations represent a financial burden for healthcare expenditures.			0.8%	5.5%	92.9%	0.8%	0.085
	Medical speciality	General Practitioners	2.2%	11.1%	86.7%		
		Pulmonologists		2.4%	96.3%	1.2%	

Supplementary Table 23. Economic impact of COPD treatment. Global results and sub-analysis by country.

		Completely disagree (1-3)	Neutral (4-6)	Completely agree (7-9)	Don't know	p value
(Items are displayed in order of higher frequency)		%				
Medicines represent the most significant source of costs in management of COPD patients (which also includes visits to healthcare professionals, exacerbations, pneumonia).	Total sample	61%	21%	17%	0.8%	0.067
	Belgium	58%	15%	27%		
	Finland	43%	43%	14%		
	Greece	76%	12%	12%		
	Norway	60%	24%	12%	4.0%	
	Portugal	79%	12%	9%		
	The Netherlands	28%	44%	28%		
At same perceived clinical added value, I prescribe the cheapest product.	Total sample	17%	28%	55%		0.001
	Belgium	12%	38%	50%		
	Finland		14%	86%		
	Greece	29%	24%	47%		
	Norway	28%	36%	36%		
	Portugal	6%	9%	85%		
	The Netherlands	22%	50%	28%		
I am sensitive to information on the financial implications of COPD management on the healthcare expenditure.	Total sample	14%	24%	61%		0.000
	Belgium	12%	35%	54%		
	Finland	14%		86%		
	Greece	24%	24%	53%		
	Norway	32%	28%	40%		
	Portugal		6%	94%		
	The Netherlands	11%	50%	39%		
Pneumonia represents a financial burden for healthcare expenditures.	Total sample	2%	12%	85%	0.8%	0.097
	Belgium		19%	81%		
	Finland			100%		
	Greece	12%	12%	71%	5.9%	
	Norway	4%	12%	84%		

	Portugal		3%	97%		
	The Netherlands		22%	78%		
Exacerbations represent a financial burden for healthcare expenditures.	Total sample	1%	6%	93%	0.8%	0.270
	Belgium		12%	88%		
	Finland			100%		
	Greece			94%	5.9%	
	Norway	4%	12%	84%		
	Portugal			100%		
	The Netherlands		6%	94%		

Supplementary Table 24. Inhaler selection: importance of patient and device characteristics. Global results and sub-analysis by medical speciality.

			Not important at all (1-3)	Neutral (4-6)	Extremely important (7-9)	Don't know	p value
			%				
Patient characteristics<br>*(Items are displayed in order of importance)*							
Age of the patient	Total sample		8.7%	19.7%	71.7%		0.375
	Medical speciality	General Practitioners	13.3%	17.8%	68.9%		
		Pulmonologists	6.1%	20.7%	73.2%		
Patient literacy/ability to understand medical information / Instruction on inhaler use	Total sample		2.4%	18.1%	79.5%		0.672
	Medical speciality	General Practitioners	2.2%	22.2%	75.6%		
		Pulmonologists	2.4%	15.9%	81.7%		
Patient inspiratory flow rate (measured or estimated)	Total sample		3.9%	15.0%	81.1%		0.363
	Medical speciality	General Practitioners	4.4%	8.9%	86.7%		
		Pulmonologists	3.7%	18.3%	78.0%		
Severity of the disease	Total sample		2.4%	15.7%	81.9%		0.463
	Medical speciality	General Practitioners	4.4%	13.3%	82.2%		
		Pulmonologists	1.2%	17.1%	81.7%		
Patient preference for the device	Total sample		2.4%	15.0%	82.7%		0.264
	Medical speciality	General Practitioners		11.1%	88.9%		
		Pulmonologists	3.7%	17.1%	79.3%		

Hand dexterity			4.7%	10.2%	84.3%	0.8%	0.337
	Medical speciality	General Practitioners	6.7%	13.3%	77.8%	2.2%	
		Pulmonologists	3.7%	8.5%	87.8%		
Device characteristics<br>*(Items are displayed in order of importance)*							
Lack of additives (e.g., lactose)	Total sample		17.3%	58.3%	23.6%	0.8%	0.284
	Medical speciality	General Practitioners	22.2%	57.8%	17.8%	2.2%	
		Pulmonologists	14.6%	58.5%	26.8%		
Size and shape			10.2%	55.9%	33.9%		0.971
	Medical speciality	General Practitioners	11.1%	55.6%	33.3%		
		Pulmonologists	9.8%	56.1%	34.1%		
Low carbon footprint	Total sample		21.3%	33.1%	44.9%	0.8%	0.402
	Medical speciality	General Practitioners	28.9%	28.9%	42.2%		
		Pulmonologists	17.1%	35.4%	46.3%	1.2%	
Re-usability (use of refills enabling longer use of device)	Total sample		11.8%	34.6%	53.5%		0.515
	Medical speciality	General Practitioners	13.3%	40.0%	46.7%		
		Pulmonologists	11.0%	31.7%	57.3%		
Propellant-free	Total sample		12.6%	32.3%	55.1%		0.070
	Medical speciality	General Practitioners	13.3%	44.4%	42.2%		
		Pulmonologists	12.2%	25.6%	62.2%		
Locking mechanism when empty	Total sample		6.3%	25.2%	68.5%		0.374
	Medical speciality	General Practitioners	2.2%	26.7%	71.1%		
		Pulmonologists	8.5%	24.4%	67.1%		
Multi-dose	Total sample		6.3%	18.1%	75.6%		0.224
	Medical speciality	General Practitioners	4.4%	11.1%	84.4%		

		Pulmonologists	7.3%	22.0%	70.7%		
Presence of dose counter	Total sample		3.1%	18.9%	77.2%	0.8%	0.514
	Medical speciality	General Practitioners	4.4%	13.3%	82.2%		
		Pulmonologists	2.4%	22.0%	74.4%	1.2%	
Level of lung deposition	Total sample		3.9%	15.0%	80.3%	0.8%	0.459
	Medical speciality	General Practitioners	4.4%	11.1%	82.2%	2.2%	
		Pulmonologists	3.7%	17.1%	79.3%		
Easy inhalation / low inspiratory effort	Total sample		0.8%	5.5%	93.7%		0.698
	Medical speciality	General Practitioners		4.4%	95.6%		
		Pulmonologists	1.2%	6.1%	92.7%		
Easy coordination of actuation and inhalation	Total sample		0.8%	2.4%	96.1%	0.8%	0.414
	Medical speciality	General Practitioners			100.0%		
		Pulmonologists	1.2%	3.7%	93.9%	1.2%	

Supplementary Table 25. Inhaler selection: importance of patient and device characteristics. Global results and sub-analysis by country.

		Not important at all (1-3)	Neutral (4-6)	Extremely important (7-9)	Don't know	p value
		%				
Patient characteristics *(Items are displayed in order of importance)*						
Age of the patient	Total sample	9%	20%	72%		0.118
	Belgium	8%	15%	77%		
	Finland		14%	86%		
	Greece		24%	76%		
	Norway	24%	24%	52%		
	Portugal	6%	12%	82%		
	The Netherlands	6%	33%	61%		
Patient literacy/ability to understand medical information / Instruction on inhaler use	Total sample	2%	18%	80%		0.024
	Belgium	4%	38%	58%		
	Finland	14%	14%	71%		
	Greece			100%		
	Norway	4%	12%	84%		
	Portugal		12%	88%		
	The Netherlands		28%	72%		
Patient inspiratory flow rate (measured or estimated)	Total sample	4%	15%	81%		0.026
	Belgium	4%	8%	88%		
	Finland			100%		
	Greece		18%	82%		
	Norway	8%	36%	56%		
	Portugal		6%	94%		
	The Netherlands	11%	17%	72%		
Severity of the disease		2%	16%	82%		0.039
	Belgium		19%	81%		
	Finland		14%	86%		
	Greece	6%	6%	88%		
	Norway	8%	36%	56%		
	Portugal		9%	91%		
	The Netherlands		6%	94%		
Patient preference for the device	Total sample	2%	15%	83%		0.093
	Belgium	8%	19%	73%		

	Finland		14%	86%		
	Greece	6%	35%	59%		
	Norway		4%	96%		
	Portugal		9%	91%		
	The Netherlands		17%	83%		
Hand dexterity		5%	10%	84%	0.8%	0.000
	Belgium		12%	88%		
	Finland		14%	86%		
	Greece			100%		
	Norway	24%	24%	48%	4.0%	
	Portugal		6%	94%		
	The Netherlands		6%	94%		
Device characteristics *(Items are displayed in order of importance)*						
Lack of additives (e.g., lactose)	Total sample	17%	58%	24%	0.8%	0.149
	Belgium	35%	54%	12%		
	Finland	14%	57%	29%		
	Greece	12%	65%	24%		
	Norway	16%	72%	8%	4.0%	
	Portugal	9%	50%	41%		
	The Netherlands	17%	56%	28%		
Size and shape	Total sample	10%	56%	34%		0.924
	Belgium	15%	54%	31%		
	Finland		71%	29%		
	Greece	12%	47%	41%		
	Norway	16%	52%	32%		
	Portugal	6%	59%	35%		
	The Netherlands	6%	61%	33%		
Low carbon footprint	Total sample	21%	33%	45%	0.8%	0.012
	Belgium	38%	38%	23%		
	Finland	14%	29%	57%		
	Greece	18%	29%	53%		
	Norway	32%	48%	20%		
	Portugal	9%	21%	71%		
	The Netherlands	11%	33%	50%	5.6%	
Re-usability (use of refills enabling longer use of device)	Total sample	12%	35%	54%		0.010
	Belgium	15%	38%	46%		

	Finland		14%	86%		
	Greece	6%	35%	59%		
	Norway	20%	60%	20%		
	Portugal	9%	15%	76%		
	The Netherlands	11%	39%	50%		
Propellant-free	Total sample	13%	32%	55%		0.000
	Belgium	15%	58%	27%		
	Finland	14%		86%		
	Greece	6%	18%	76%		
	Norway	20%	64%	16%		
	Portugal	6%	12%	82%		
	The Netherlands	17%	17%	67%		
Locking mechanism when empty		6%	25%	69%		0.000
	Belgium	8%	31%	62%		
	Finland	14%	14%	71%		
	Greece	18%	12%	71%		
	Norway	8%	60%	32%		
	Portugal		6%	94%		
	The Netherlands		22%	78%		
Multi-dose	Total sample	6%	18%	76%		0.252
	Belgium	4%	27%	69%		
	Finland			100%		
	Greece	12%	12%	76%		
	Norway	8%	28%	64%		
	Portugal	9%	6%	85%		
	The Netherlands		28%	72%		
Presence of dose counter	Total sample	3%	19%	77%	0.8%	0.031
	Belgium	4%	23%	73%		
	Finland		14%	86%		
	Greece		29%	71%		
	Norway	8%	40%	48%	4.0%	
	Portugal		6%	94%		
	The Netherlands	6%		94%		
Level of lung deposition	Total sample	4%	15%	80%	0.8%	0.036
	Belgium	4%	27%	69%		
	Finland			100%		

	Greece			100%		
	Norway	12%	32%	56%		
	Portugal		6%	91%	2.9%	
	The Netherlands	6%	11%	83%		
Easy inhalation / low inspiratory effort	Total sample	1%	6%	94%		0.006
	Belgium	4%	4%	92%		
	Finland			100%		
	Greece			100%		
	Norway		24%	76%		
	Portugal			100%		
	The Netherlands			100%		
Easy coordination of actuation and inhalation	Total sample	1%	2%	96%	0.8%	0.839
	Belgium	4%	4%	92%		
	Finland			100%		
	Greece			100%		
	Norway		4%	92%	4.0%	
	Portugal		3%	97%		
	The Netherlands			100%		

Supplementary Table 26. Participant's knowledge and opinions on COPD devices characteristics. Global results and sub-analysis by medical speciality and by country.

	Device	High level of lung deposition	Independent of the patient's inspiratory flow rate	Effective when patient is limited in inspiratory capacity (e.g., during exacerbation)	Propellant-free	Low carbon footprint	Reusable	I am most experienced with…	Easy for the patient to use	High patient satisfaction	Easy coordination
		n (%)									
**Global results**											
**Total sample (N=127)**	DPI (dry powder inhaler)	8 (6,3%)	3 (2,4%)	1 (0,8%)	93 (73,2%)	31 (24,4%)	16 (12,6%)	89 (70,1%)	106 (84,1%)	76 (59,8%)	107 (84,3%)
	pMDI (pressured metered dose inhaler)	26 (20,5%)	32 (25,4%)	42 (33,3%)	4 (3,1%)	3 (2,4%)	5 (3,9%)	55 (43,3%)	9 (7,1%)	19 (15%)	6 (4,7%)
	Soft mist inhaler	121 (95,3%)	118 (93,7%)	111 (88,1%)	36 (28,3%)	35 (27,6%)	97 (76,4%)	71 (55,9%)	40 (31,7%)	75 (59,1%)	49 (38,6%)
	Don't know	0 (0%)	1 (0,8%)	1 (0,8%)	19 (15%)	72 (56,7%)	20 (15,7%)	2 (1,6%)	2 (1,6%)	10 (7,9%)	2 (1,6%)
**Results by speciality**											
**Pulmonologists (N=82)**	DPI (dry powder inhaler)	2.4%	2.4%	1.2%	78.0%	24.4%	11.0%	73.2%	87.8%	58.5%	87.8%
	pMDI (pressured metered dose inhaler)	19.5%	23.2%	34.1%	4.9%	3.7%	2.4%	50.0%	7.3%	15.9%	3.7%
	Soft mist inhaler	95.1%	93.9%	87.8%	29.3%	32.9%	80.5%	56.1%	25.6%	54.9%	32.9%
	Don't know	0.0%	1.2%	1.2%	8.5%	53.7%	12.2%	1.2%	1.2%	8.5%	2.4%
**GPs (N=45)**	DPI (dry powder inhaler)	13.3%	2.3%	0.0%	64.4%	24.4%	15.6%	64.4%	77.3%	62.2%	77.8%
	pMDI (pressured metered dose inhaler)	22.2%	29.5%	31.8%	0.0%	0.0%	6.7%	31.1%	6.8%	13.3%	6.7%

	Soft mist inhaler	95.6%	93.2%	88.6%	26.7%	17.8%	68.9%	55.6%	43.2%	66.7%	48.9%
	Don’t know	0.0%	0.0%	0.0%	26.7%	62.2%	22.2%	2.2%	2.3%	6.7%	0.0%
p value		0.112	0.881	0.883	0.013	0.207	0.178	0.242	0.154	0.714	0.135
**Results by country**											
**Belgium (N=26)**	DPI (dry powder inhaler)	0.0%	0.0%	0.0%	73.1%	23.1%	15.4%	73.1%	84.6%	61.5%	88.5%
	pMDI (pressured metered dose inhaler)	11.5%	19.2%	23.1%	0.0%	0.0%	3.8%	42.3%	7.7%	15.4%	3.8%
	Soft mist inhaler	100.0%	100.0%	100.0%	34.6%	26.9%	84.6%	61.5%	38.5%	57.7%	42.3%
	Don’t know	0.0%	0.0%	0.0%	15.4%	61.5%	7.7%	0.0%	0.0%	19.2%	0.0%
**Finland (N=7)**	DPI (dry powder inhaler)	14.3%	0.0%	0.0%	85.7%	28.6%	0.0%	71.4%	85.7%	71.4%	100.0%
	pMDI (pressured metered dose inhaler)	14.3%	28.6%	42.9%	0.0%	0.0%	0.0%	14.3%	0.0%	0.0%	0.0%
	Soft mist inhaler	100.0%	100.0%	100.0%	14.3%	0.0%	71.4%	42.9%	28.6%	57.1%	14.3%
	Don’t know	0.0%	0.0%	0.0%	14.3%	71.4%	28.6%	0.0%	0.0%	0.0%	0.0%
**Greece (N=17)**	DPI (dry powder inhaler)	0.0%	0.0%	0.0%	88.2%	29.4%	11.8%	64.7%	82.4%	58.8%	82.4%
	pMDI (pressured metered dose inhaler)	11.8%	5.9%	11.8%	5.9%	0.0%	0.0%	47.1%	0.0%	17.6%	0.0%
	Soft mist inhaler	100.0%	100.0%	94.1%	29.4%	41.2%	82.4%	47.1%	41.2%	64.7%	47.1%
	Don’t know	0.0%	0.0%	0.0%	0.0%	47.1%	5.9%	5.9%	0.0%	0.0%	5.9%
**Norway (N=25)**	DPI (dry powder inhaler)	8.0%	8.0%	4.0%	64.0%	32.0%	4.0%	60.0%	76.0%	44.0%	80.0%
	pMDI (pressured metered dose inhaler)	20.0%	28.0%	32.0%	4.0%	12.0%	4.0%	32.0%	12.0%	24.0%	0.0%
	Soft mist inhaler	92.0%	88.0%	92.0%	24.0%	20.0%	64.0%	72.0%	40.0%	64.0%	44.0%

	Don’t know	0.0%	4.0%	4.0%	24.0%	52.0%	32.0%	0.0%	4.0%	12.0%	4.0%
**Portugal (N=34)**	DPI (dry powder inhaler)	8.8%	0.0%	0.0%	70.6%	8.8%	20.6%	82.4%	87.9%	64.7%	85.3%
	pMDI (pressured metered dose inhaler)	5.9%	24.2%	33.3%	0.0%	0.0%	5.9%	44.1%	6.1%	2.9%	0.0%
	Soft mist inhaler	100.0%	93.9%	87.9%	41.2%	35.3%	73.5%	61.8%	27.3%	64.7%	44.1%
	Don’t know	0.0%	0.0%	0.0%	14.7%	58.8%	17.6%	2.9%	3.0%	5.9%	0.0%
**The Netherlands (N=18)**	DPI (dry powder inhaler)	11.1%	5.6%	0.0%	72.2%	38.9%	11.1%	61.1%	88.9%	66.7%	77.8%
	pMDI (pressured metered dose inhaler)	72.2%	50.0%	66.7%	11.1%	0.0%	5.6%	66.7%	11.1%	27.8%	27.8%
	Soft mist inhaler	77.8%	83.3%	55.6%	5.6%	22.2%	83.3%	27.8%	11.1%	38.9%	16.7%
	Don’t know	0.0%	0.0%	0.0%	16.7%	55.6%	5.6%	0.0%	0.0%	0.0%	0.0%
p value		**>0.0001**	0.108	**0.001**	0.263	0.105	0.435	0.153	0.853	0.172	**0.006**

Supplementary Table 27. Clinicians' opinion on the potential impact of COPD treatment optimization. Global results and sub-analysis by medical speciality.

			Completely disagree (1-3)	Neutral (4-6)	Completely agree (7-9)	Don't know	p value
			%				
**Switching from both LABA/ICS or LAMA/LABA/ICS to LABA/LAMA should be considered if…** *(Items are displayed in order of higher frequency)*							
history or current pneumonia	Total sample		3.1%	24.4%	72.4%		0.601
	Medical speciality	General Practitioners	2.2%	20.0%	77.8%		
		Pulmonologists	3.7%	26.8%	69.5%		
there are other side effects possibly related to ICS	Total sample		2.4%	23.6%	74.0%		0.306
	Medical speciality	General Practitioners		20.0%	80.0%		
		Pulmonologists	3.7%	25.6%	70.7%		
there has not been a therapeutic response to ICS treatment.	Total sample		2.4%	15.7%	81.9%		0.010
	Medical speciality	General Practitioners	6.7%	6.7%	86.7%		
		Pulmonologists		20.7%	79.3%		
ICS therapy is no longer indicated.	Total sample		3.1%	5.5%	91.3%		0.128
	Medical speciality	General Practitioners	6.7%	2.2%	91.1%		
		Pulmonologists	1.2%	7.3%	91.5%		
**ICS discontinuation should be considered in patients with:** *(Items are displayed in order of higher frequency)*							
Diabetes, even if eosinophil count >= 300 cells /µl	Total sample		19.7%	67.7%	7.1%	5.5%	0.004
	Medical speciality	General Practitioners	15.6%	71.1%		13.3%	
		Pulmonologists	22.0%	65.9%	11.0%	1.2%	
Osteoporosis, even if eosinophil count >= 300 cells /µl	Total sample		17.3%	70.1%	8.7%	3.9%	0.006
	Medical speciality	General Practitioners	8.9%	73.3%	6.7%	11.1%	
		Pulmonologists	22.0%	68.3%	9.8%		
Smoker patients, even if eosinophil count >= 300 cells /µl	Total sample		20.5%	66.9%	9.4%	3.1%	0.044
	Medical speciality	General Practitioners	17.8%	66.7%	6.7%	8.9%	

		Pulmonologists	22.0%	67.1%	11.0%		
Patients with low eosinophil count (n=<100), even if they suffer 1 severe exacerbation with hospitalization a year	Total sample		33.1%	50.4%	13.4%	3.1%	0.044
	Medical speciality	General Practitioners	28.9%	46.7%	15.6%	8.9%	
		Pulmonologists	35.4%	52.4%	12.2%		
No therapeutic response, even if eosinophil count >= 300 cells /µl	Total sample		16.5%	60.6%	19.7%	3.1%	0.008
	Medical speciality	General Practitioners	8.9%	68.9%	13.3%	8.9%	
		Pulmonologists	20.7%	56.1%	23.2%		
Adverse events, even if eosinophil count >= 300 cells /µl	Total sample		11.0%	56.7%	28.3%	3.9%	0.010
	Medical speciality	General Practitioners	8.9%	60.0%	20.0%	11.1%	
		Pulmonologists	12.2%	54.9%	32.9%		
Smoker patients, even if eosinophil count is 100-300 cells/µl	Total sample		9.4%	43.3%	44.9%	2.4%	0.038
	Medical speciality	General Practitioners	4.4%	37.8%	51.1%	6.7%	
		Pulmonologists	12.2%	46.3%	41.5%		
Diabetes, even if eosinophil count is 100-300 cells/µl	Total sample		11.0%	37.0%	47.2%	4.7%	0.022
	Medical speciality	General Practitioners	4.4%	42.2%	42.2%	11.1%	
		Pulmonologists	14.6%	34.1%	50.0%	1.2%	
Osteoporosis, even if eosinophil count is 100-300 cells/µl	Total sample		8.7%	40.2%	48.0%	3.1%	0.032
	Medical speciality	General Practitioners	4.4%	37.8%	48.9%	8.9%	
		Pulmonologists	11.0%	41.5%	47.6%		
Patients with low eosinophil count (n=<100) even if they suffer 1 moderate exacerbation a year	Total sample		9.4%	39.4%	48.0%	3.1%	0.006
	Medical speciality	General Practitioners	4.4%	28.9%	57.8%	8.9%	
		Pulmonologists	12.2%	45.1%	42.7%		
Adverse events, even if eosinophil count is 100-300 cells/µl	Total sample		3.1%	20.5%	73.2%	3.1%	0.016
	Medical speciality	General Practitioners		15.6%	75.6%	8.9%	
		Pulmonologists	4.9%	23.2%	72.0%		

No therapeutic response, even if eosinophil count is 100-300 cells/µl	Total sample		2.4%	20.5%	74.0%	3.1%	0.021
	Medical speciality	General Practitioners		15.6%	75.6%	8.9%	
		Pulmonologists	3.7%	23.2%	73.2%		

Supplementary Table 28. Clinicians' opinion on the potential impact of COPD treatment optimization. Global results and sub-analysis by country.

		Completely disagree (1-3)	Neutral (4-6)	Completely agree (7-9)	Don't know	p value
		%				
**Switching from both LABA/ICS or LAMA/LABA/ICS to LABA/LAMA should be considered if…** *(Items are displayed in order of higher frequency)*						
history or current pneumonia	Total sample	3%	24%	72%		0.105
	Belgium		31%	69%		
	Finland		29%	71%		
	Greece	6%	35%	59%		
	Norway	8%	24%	68%		
	Portugal		6%	94%		
	The Netherlands	6%	39%	56%		
there are other side effects possibly related to ICS (n=like osteoporosis, cataract…)	Total sample	2%	24%	74%		0.368
	Belgium	8%	35%	58%		
	Finland		14%	86%		
	Greece	6%	18%	76%		
	Norway		20%	80%		
	Portugal		18%	82%		
	The Netherlands		33%	67%		
there has not been a therapeutic response to ICS treatment.	Total sample	2%	16%	82%		0.061
	Belgium		38%	62%		
	Finland			100%		
	Greece		12%	88%		
	Norway	4%	12%	84%		
	Portugal	6%	6%	88%		
	The Netherlands		17%	83%		
ICS therapy is no longer indicated.	Total sample	3%	6%	91%		0.049
	Belgium	4%	19%	77%		
	Finland			100%		
	Greece		12%	88%		
	Norway			100%		
	Portugal	6%		94%		
	The Netherlands	6%		94%		
**ICS discontinuation should be considered in patients with:** *(Items are displayed in order of higher frequency)*						
Diabetes, even if eosinophil count >= 300 cells /µl	Total sample	20%	68%	7%	5.5%	0.191

	Belgium	23%	62%	8%	7.7%	
	Finland	29%	71%			
	Greece	6%	71%	24%		
	Norway	16%	72%	4%	8.0%	
	Portugal	26%	71%	3%		
	The Netherlands	17%	61%	6%	16.7%	
Osteoporosis, even if eosinophil count >= 300 cells /µl	Total sample	17%	70%	9%	3.9%	0.728
	Belgium	23%	58%	12%	7.7%	
	Finland	14%	86%			
	Greece	18%	76%	6%		
	Norway	12%	72%	12%	4.0%	
	Portugal	24%	68%	9%		
	The Netherlands	6%	78%	6%	11.1%	
Smoker patients, even if eosinophil count >= 300 cells /µl	Total sample	20%	67%	9%	3.1%	0.223
	Belgium	27%	50%	19%	3.8%	
	Finland		100%			
	Greece	18%	71%	12%		
	Norway	16%	80%		4.0%	
	Portugal	29%	62%	9%		
	The Netherlands	11%	67%	11%	11.1%	
Patients with low eosinophil count (n=<100), even if they suffer 1 severe exacerbation with hospitalization a year	Total sample	33%	50%	13%	3.1%	0.257
	Belgium	35%	42%	19%	3.8%	
	Finland	29%	57%	14%		
	Greece	41%	59%			
	Norway	12%	68%	16%	4.0%	
	Portugal	35%	50%	15%		
	The Netherlands	50%	28%	11%	11.1%	
No therapeutic response, even if eosinophil count >= 300 cells /µl	Total sample	17%	61%	20%	3.1%	0.454
	Belgium	27%	42%	23%	7.7%	
	Finland	29%	57%	14%		
	Greece	18%	65%	18%		
	Norway	16%	64%	20%		
	Portugal	9%	68%	24%		
	The Netherlands	11%	67%	11%	11.1%	
Adverse events, even if eosinophil count >= 300 cells /µl	Total sample	11%	57%	28%	3.9%	0.670
	Belgium	15%	46%	31%	7.7%	

	Finland	14%	71%	14%		
	Greece		65%	35%		
	Norway	12%	60%	24%	4.0%	
	Portugal	9%	56%	35%		
	The Netherlands	17%	56%	17%	11.1%	
Smoker patients, even if eosinophil count is 100-300 cells/µl	Total sample	9%	43%	45%	2.4%	0.217
	Belgium	12%	35%	50%	3.8%	
	Finland		57%	43%		
	Greece	6%	59%	35%		
	Norway	8%	64%	28%		
	Portugal	12%	35%	53%		
	The Netherlands	11%	22%	56%	11.1%	
Diabetes, even if eosinophil count is 100-300 cells/µl	Total sample	11%	37%	47%	4.7%	0.277
	Belgium	12%	23%	58%	7.7%	
	Finland		29%	71%		
	Greece	12%	35%	53%		
	Norway	16%	44%	36%	4.0%	
	Portugal	9%	38%	53%		
	The Netherlands	11%	50%	22%	16.7%	
Osteoporosis, even if eosinophil count is 100-300 cells/µl	Total sample	9%	40%	48%	3.1%	0.130
	Belgium	12%	23%	58%	7.7%	
	Finland		29%	71%		
	Greece	6%	53%	41%		
	Norway	12%	40%	48%		
	Portugal	9%	35%	56%		
	The Netherlands	6%	67%	17%	11.1%	
Patients with low eosinophil count (n=<100) even if they suffer 1 moderate exacerbation a year	Total sample	9%	39%	48%	3.1%	0.177
	Belgium	4%	42%	50%	3.8%	
	Finland	14%	43%	43%		
	Greece	29%	47%	24%		
	Norway	12%	36%	48%	4.0%	
	Portugal	3%	35%	62%		
	The Netherlands	6%	39%	44%	11.1%	
Adverse events, even if eosinophil count is 100-300 cells/µl	Total sample	3%	20%	73%	3.1%	0.016
	Belgium	4%	15%	73%	7.7%	
	Finland		29%	71%		

	Greece		24%	76%		
	Norway	4%	36%	60%		
	Portugal		3%	97%		
	The Netherlands	11%	33%	44%	11.1%	
No therapeutic response, even if eosinophil count is 100-300 cells/µl	Total sample	2%	20%	74%	3.1%	0.153
	Belgium		23%	69%	7.7%	
	Finland		14%	86%		
	Greece		24%	76%		
	Norway	8%	28%	64%		
	Portugal		9%	91%		
	The Netherlands	6%	28%	56%	11.1%	

Supplementary Table 29. Important aspects for treatment decisions. Global results and sub-analysis by medical speciality and by country.

Aspects for treatment initiation decisions	Total sample (N=127)	Results by speciality			Results by country						
		GPs (N=45)	Pulmonologists (N=82)	p value	Belgium (N=26)	Finland (N=7)	Greece (N=17)	Norway (N=25)	Portugal (N=34)	The Netherlands (N=18)	P value
	**Average score**	**Average score**			Average score						
	**Mean (SD)**	**Mean (SD)**			Mean (SD)						
**Reducing symptoms (e.g., breathlessness)**	3.0 (1.7)	3.4 (1.7)	2.8 (1.6)	**0.019**	2.8 (1.7)	3.6 (0.8)	3.2 (1.6)	2.7 (1.6)	3.5 (1.8)	2.6 (1.6)	0.336
**Increasing patient's quality of life**	3.2 (2.2)	3.1 (2.4)	3.2 (2.2)	0.227	3.4 (2.3)	2.3 (1.4)	3.0 (2.4)	3.2 (2.4)	3.8 (2.3)	2.4 (1.9)	0.303
**Reducing future risk of exacerbation**	3.5 (1.6)	3.6 (1.6)	3.4 (1.7)	0.66	3.7 (1.8)	3.4 (2.4)	3.3 (1.5)	2.9 (1)	3.4 (1.7)	4.3 (1.5)	0.167
**Improving exercise tolerance**	4.0 (1.5)	4.0 (1.6)	4.0 (1.4)	0.685	3.8 (1.3)	4.6 (1.7)	4.6 (1.7)	4.0 (1.5)	4.1 (1.5)	3.2 (1.2)	0.091
**Reducing the risk of adverse events that may affect patient's health in the future**	4.5 (2.1)	4.1 (2.1)	4.7 (2.1)	**0.031**	5.2 (2.0)	4.4 (2.1)	4.4 (2.3)	4.4 (2)	3.5 (2.3)	5.6 (1.4)	**0.01**
**Choosing the right inhaler according to patient's ability and clinical condition**	4.7 (1.8)	4.7 (1.8)	4.7 (1.8)	0.527	4.3 (1.8)	5.0 (2.1)	4.6 (2.1)	5.6 (1.6)	4.4 (1.6)	4.6 (1.9)	0.122
**Optimizing lung function**	5.1 (1.9)	5.0 (2.0)	5.2 (1.9)	0.773	4.8 (2.1)	4.7 (2.6)	4.9 (1.6)	5.2 (1.6)	5.4 (2.0)	5.4 (2.0)	0.828

For the interpretation of the Average Score, aspects were defined by: 1 "The most important aspect " and 7 "the least important aspect".
