# Supplementary material for: Investigating the rationale for COPD maintenance therapy prescription across Europe, findings from a multi-country study
Source: NPJ Prim Care Respir Med. 2023 May 3;33:18. doi: 10.1038/s41533-023-00334-x (PMC10154184; doi:10.1038/s41533-023-00334-x)
Supplement: Supplementary file 3 — Appendix 1 [file 41533_2023_334_MOESM3_ESM.pdf]

# 1. EXPERT PANELISTS AND COPD PATIENTS PROFILE

We kindly ask you to reply to the following questions with the aim to define your profile as well as of your COPD patients. Questions of Section 1 will not be repeated in the 2nd wave.

1.1 Age: ___ years old

1.2 Please indicate your medical specialty (*single answer)*:

- ❑ General practitioner
- ❑ Internal medicine
- ❑ Pulmonologist
- ❑ Other, please, specify: ________

1.3 What type of center do you work at? (*multiple choice)*

- o University hospital
- o Non-university hospital
- o Primary care center (≥ 5 physicians)
- o Individual office/office with few healthcare professionals

1.4 Do you work at a public, private or both type of centers? (*single answer)*

- ❑ Public center
- ❑ Private center
- ❑ Both

1.5 In which country are you based? (*single answer)*

- ❑ Belgium
- ❑ Finland
- ❑ Greece
- ❑ Netherlands
- ❑ Norway
- ❑ Portugal

1.6 For how long have you been treating patients with COPD? (*single answer)*

- ❑ 3-5 years
- ❑ 5-10 years
- ❑ 10-15 years
- ❑ >15 years

1.7 On average, how many individual COPD patients did you personally see, treat or diagnose in the last **6 months**?)?
*(please think of the number of patients, not visits)*

_____ Number of patients

1.8 Among your [show answer from Q1.7] COPD patients, please classify them, approximately, according to the type of patients:

a) First visit patients (newly diagnosed and treatment initiation decisions made): ____%
b) Follow-up patients (followed-up either by yourself or your staff reporting to you to make treatment decisions): ____%

1.9 Among your [show answer from Q1.7] COPD patients visited last 6 months, what percentage are current smokers, approximately?
____%

1.10 According to your estimation, among your [show answer from Q1.7] COPD patients, please indicate, approximately, which percentage of them suffer the following comorbidities:

| | |
|---|---|
| **Pulmonary pathology** | |
| Asthma | % |
| Chronic bronchitis | % |
| Pulmonary hypertension | % |
| **Mental health** | |
| Anxiety / depression | % |
| Dementia | % |
| **Metabolic disease** | |
| Dyslipidemia | % |
| Obesity | % |
| Osteoporosis | % |
| **Cardiovascular** | |
| Hypertension | % |
| Coronary heart disease | % |
| Heart failure | % |
| **Other comorbidities** | |
| Renal comorbidities (including chronic kidney failure) | % |
| Gastroenterological comorbidities (including dyspepsia and Gastroesophageal reflux) | % |
| Osteoarthritis, degenerative joint disease | % |
| **No comorbidities** | |
| No comorbidities | % |

**the total can sum> 100%*

1.11 Please indicate how frequently you use the following tests for your stable COPD patients. Please, mark the best fitting frequency per row. *(single answer)*

| | Frequency | | | | |
|---|---|---|---|---|---|
| | **Every 3 months** | **Every 6 months** | **Once a year** | **Less than once a year** | **Never** |
| CCQ / CAT test and/or mMRC scale | ☐ | ☐ | ☐ | ☐ | ☐ |
| St George's Respiratory Questionnaire | ☐ | ☐ | ☐ | ☐ | ☐ |
| Physical examination | ☐ | ☐ | ☐ | ☐ | ☐ |
| Spirometry | ☐ | ☐ | ☐ | ☐ | ☐ |
| Bronchodilator reversibility tests | ☐ | ☐ | ☐ | ☐ | ☐ |
| Blood eosinophils count | ☐ | ☐ | ☐ | ☐ | ☐ |
| X-ray/X-thorax | ☐ | ☐ | ☐ | ☐ | ☐ |
| CT scan | ☐ | ☐ | ☐ | ☐ | ☐ |

*CCQ: Clinical COPD Questionnaire; CAT: COPD Assessment Test™: mMRC: modified Medical Research Council dyspnea questionnaire*

1.12 In reference to the blood eosinophil count, please indicate when do you usually measure it (*multiple choice*):

- o At treatment initiation
- o When considering a change of therapy
- o Routinely in a treated patient
- o At the time of exacerbation
- o After an exacerbation
- o Never
- o Other, please specify: _______________

1.13 Among your [show answer from Q1.7] COPD patients seen in the last **6 months**, please indicate the approximate percentage of patients for whom eosinophils have been measured:

______%

## 2. CURRENT MANAGEMENT OF COPD PATIENTS

2.1. What are your treatment decisions based on? *(please rank them from the most important source (1) to the least (5))*

- ❑ My own experience
- ❑ A national guideline
- ❑ A local guideline
- ❑ GOLD Report (Global Initiative for Chronic Obstructive Lung Disease)
- ❑ My colleagues' advise

### COPD treatment: initiation

2.2. Are you familiar with the **GOLD (A, B, C, D) classification for initial therapy**? (*single answer)*

- ❑ Yes
- ❑ No

**If yes:*

2.2.1. Do you usually use it? (*single answer)*

- ❑ Yes
- ❑ No

**If 2.2=No, the following text and questions 2.3 and 2.4 will be skipped.*

***The next questions are related to the 2020 GOLD Report (Global Initiative for Obstructive Lung disease) classification (A, B, C, D) system based on symptom burden and risk of exacerbation.***

Please click here for your review as a bibliographical support.

*(THIS IS A POP UP THAT WILL APPEAR AT THE WEB SITE IN CASE THEY CLICK)*

| **Moderate or severe exacerbation history** | | |
|---|---|---|
| **≥ 2 or ≥ 1 leading to hospital admission** | **C** | **D** |
| **0 or 1 (not leading to hospital admission)** | **A** | **B** |
| | **mMRC 0-1 CAT <10** | **mMRC ≥ 2 CAT ≥ 10** |
| | **Symptoms** | |

*mMRC: modified Medical Research Council dyspnea questionnaire;*
*CAT™: COPD Assessment Test™.*

*Global Initiative for Chronic Obstructive Lung Disease. Global strategy for the diagnosis, management, and prevention of chronic obstructive pulmonary disease 2020 report. https://goldcopd.org/wp-content/uploads/2019/11/GOLD-2020-REPORT-ver1.0wms.pdf.*

2.3. Among your [show answer from (**Q1.7***Q1.8**)/(100)] **new** COPD patients seen in the **last 6 months**, please classify them, approximately, according to the A, B, C, D classification:

| | |
|---|---|
| Group A | % |
| Group B | % |
| Group C | % |
| Group D | % |
| | **the total must sum 100%* |

2.4. Please distribute your [show answer from (**Q1.7***Q1.8**)/(100)], **new** COPD patients seen in the **last 6 months** according to the **type of treatment they are initially prescribed, per GOLD category, approximately** *(independently of being fixed or free dose combination):*

| | | |
|---|---|---|
| **GOLD Group A** | Short-acting inhaled bronchodilator as only medication | % |
| | Monotherapy with a long acting muscarinic antagonist (LAMA) | % |
| | Monotherapy with a long acting beta-agonist (LABA) | % |
| | Combination therapy with LABA/ICS | % |
| | Combination therapy with LABA/LAMA | |
| | Triple therapy with LABA/LAMA/ICS | % |
| | Monotherapy with ICS | % |
| | Other, please specify: ______________ | % |
| | **the total must sum 100%* | |
| **GOLD Group B** | Short-acting inhaled bronchodilator as only medication | % |
| | Monotherapy with a long acting muscarinic antagonist (LAMA) | % |
| | Monotherapy with a long acting beta-agonist (LABA) | % |
| | Combination therapy with LABA/ICS | % |
| | Combination therapy with LABA/LAMA | |
| | Triple therapy with LABA/LAMA/ICS | % |
| | Monotherapy with ICS | % |
| | Other, please specify: ___________ | % |
| | **the total must sum 100%* | |
| **GOLD Group C** | Short-acting inhaled bronchodilator as only medication | % |
| | Monotherapy with a long acting muscarinic antagonist (LAMA) | % |
| | Monotherapy with a long acting beta-agonist (LABA) | % |
| | Combination therapy with LABA/ICS | % |
| | Combination therapy with LABA/LAMA | |
| | Triple therapy with LABA/LAMA/ICS | % |
| | Monotherapy with ICS | % |
| | Other, please, specify: _________ | % |
| | **the total must sum 100%* | |
| **GOLD Group D** | Short-acting inhaled bronchodilator as only medication | % |
| | Monotherapy with a long acting muscarinic antagonist (LAMA) | % |
| | Monotherapy with a long acting beta-agonist (LABA) | % |

| | | |
|---|---|---|
| | Combination therapy with LABA/ICS | % |
| | Combination therapy with LABA/LAMA | |
| | Triple therapy with LABA/LAMA/ICS | % |
| | Monotherapy with ICS | % |
| | Other, please, specify: ___________ | % |
| | **the total must sum 100%* | |

2.5. Which aspects are the most important for you when **initiating a COPD treatment**? Please order the following aspects according to their importance *(being 1 the most important and 7 the least important):*

- ❑ Reducing the risk of adverse events that may affect patient's health in the future
- ❑ Reducing future risk of exacerbations
- ❑ Reducing symptoms (e.g. breathlessness)
- ❑ Improving exercise tolerance
- ❑ Choosing the right inhaler according to patient's ability and clinical condition
- ❑ Optimizing lung function
- ❑ Increasing patient's quality of life

2.6. From your point of view, how important are the following criteria for the selection of an **initial COPD treatment**? *(being 1 not important at all and 9 extremely important)*

| | **Not important at all** | | | | | | | **Extremely important** | **Don't know** |
|---|---|---|---|---|---|---|---|---|---|
| | 1 | 2 | 3 | 4 | 5 | 6 | 7 | 8 | 9 | |
| a. Age | ☐ | ☐ | ☐ | ☐ | ☐ | ☐ | ☐ | ☐ | ☐ | ☐ |
| b. Smoking status | ☐ | ☐ | ☐ | ☐ | ☐ | ☐ | ☐ | ☐ | ☐ | ☐ |
| c. Breathlessness | ☐ | ☐ | ☐ | ☐ | ☐ | ☐ | ☐ | ☐ | ☐ | ☐ |
| d. Exercise limitation | ☐ | ☐ | ☐ | ☐ | ☐ | ☐ | ☐ | ☐ | ☐ | ☐ |
| e. Previous exacerbations | ☐ | ☐ | ☐ | ☐ | ☐ | ☐ | ☐ | ☐ | ☐ | ☐ |
| f. History of pneumonia | ☐ | ☐ | ☐ | ☐ | ☐ | ☐ | ☐ | ☐ | ☐ | ☐ |
| g. Blood eosinophil count | ☐ | ☐ | ☐ | ☐ | ☐ | ☐ | ☐ | ☐ | ☐ | ☐ |
| h. Co-morbidities: | | | | | | | | | | |
| i. Cardiovascular | ☐ | ☐ | ☐ | ☐ | ☐ | ☐ | ☐ | ☐ | ☐ | ☐ |
| ii. Diabetes | ☐ | ☐ | ☐ | ☐ | ☐ | ☐ | ☐ | ☐ | ☐ | ☐ |
| iii. Osteoporosis/ osteopenia | ☐ | ☐ | ☐ | ☐ | ☐ | ☐ | ☐ | ☐ | ☐ | ☐ |
| iv. Current asthma | ☐ | ☐ | ☐ | ☐ | ☐ | ☐ | ☐ | ☐ | ☐ | ☐ |
| v. History of asthma | ☐ | ☐ | ☐ | ☐ | ☐ | ☐ | ☐ | ☐ | ☐ | ☐ |
| vi. Anxiety/ Depression | ☐ | ☐ | ☐ | ☐ | ☐ | ☐ | ☐ | ☐ | ☐ | ☐ |
| vii. Dementia | ☐ | ☐ | ☐ | ☐ | ☐ | ☐ | ☐ | ☐ | ☐ | ☐ |

| | | | | | | | | | | |
|---|---|---|---|---|---|---|---|---|---|---|
| viii. Osteoarthritis/ degenerative joint disease | ☐ | ☐ | ☐ | ☐ | ☐ | ☐ | ☐ | ☐ | ☐ | ☐ |
| i. The type of inhaler device | ☐ | ☐ | ☐ | ☐ | ☐ | ☐ | ☐ | ☐ | ☐ | ☐ |
| j. Posology | ☐ | ☐ | ☐ | ☐ | ☐ | ☐ | ☐ | ☐ | ☐ | ☐ |
| k. Ability to inhale | ☐ | ☐ | ☐ | ☐ | ☐ | ☐ | ☐ | ☐ | ☐ | ☐ |
| l. Carbon footprint of the inhaler | ☐ | ☐ | ☐ | ☐ | ☐ | ☐ | ☐ | ☐ | ☐ | ☐ |
| m. Price of the medicine | ☐ | ☐ | ☐ | ☐ | ☐ | ☐ | ☐ | ☐ | ☐ | ☐ |
| n. Reimbursement criteria | ☐ | ☐ | ☐ | ☐ | ☐ | ☐ | ☐ | ☐ | ☐ | ☐ |
| o. GOLD Report | ☐ | ☐ | ☐ | ☐ | ☐ | ☐ | ☐ | ☐ | ☐ | ☐ |
| p. National/local guidelines | ☐ | ☐ | ☐ | ☐ | ☐ | ☐ | ☐ | ☐ | ☐ | ☐ |
| q. Others. Please, specify: _________ | ☐ | ☐ | ☐ | ☐ | ☐ | ☐ | ☐ | ☐ | ☐ | ☐ |

2.7. Please review the following **patient profiles** and decide whether you would prescribe LAMA, LAMA/LABA, LABA/ICS, TRIPLE THERAPY or another alternative **as initial treatment.**

| |
|---|
| UP TO 144 DIFFERENT PATIENT PROFILES WILL BE CREATED BASED ON THE RANDOM MIX OF THE FOLLOWING CRITERIA (every panelist will have to classify up to 12 different profiles): |

1. Smoking status:
   a. Current smoker
   b. Non-smoker / Former smoker
2. Lung Function
   a. $FEV_1 < 50\%$
   b. $FEV_1 >= 50\%$
3. Symptoms (cough, sputum, breathlessness)
   a. Mild
   b. Moderate
   c. Severe
4. Number of the exacerbations in the last year
   a. None
   b. 1 or more without hospitalization
   c. 1 or more with hospitalization
5. Blood eosinophil count:
   a. < 100 eosinophil / µl
   b. 100-300 eosinophil / µl
   c. ≥300 eosinophil / µl
   d. Data missing / unknown

| |
|---|
| TREATMENT CLASSIFICATION: |

| | | |
|---|---|---|
| LAMA/LABA therapy | LABA/ICS therapy | TRIPLE THERAPY (LAMA/LABA/ICS) |
| LAMA only | OTHERS. Specify | Don't know / More data needed. Please, indicate what information is missing: ____ |

## COPD treatment: maintenance

2.8. Are you familiar with the **2020 GOLD follow-up pharmacological treatment algorithm for COPD**? (*single answer)*

- ❑ Yes
- ❑ No

**If yes:*

2.8.1. Do you decide on follow-up treatment based upon the suggested treatable traits? (*single answer)*

- ❑ Yes
- ❑ No
- ❑ Sometimes

Please click here for your review as a bibliographical support for the new 2020 GOLD follow-up pharmacological treatment algorithm for COPD

1. Response to initial treatment is appropriate, maintain it.
2. If not:
   - ✓ Consider the predominant treatable trait to target (dyspnea or exacerbations)
   - ✓ Place patient in box corresponding to current treatment & follow indications
   - ✓ Assess response, adjust and review

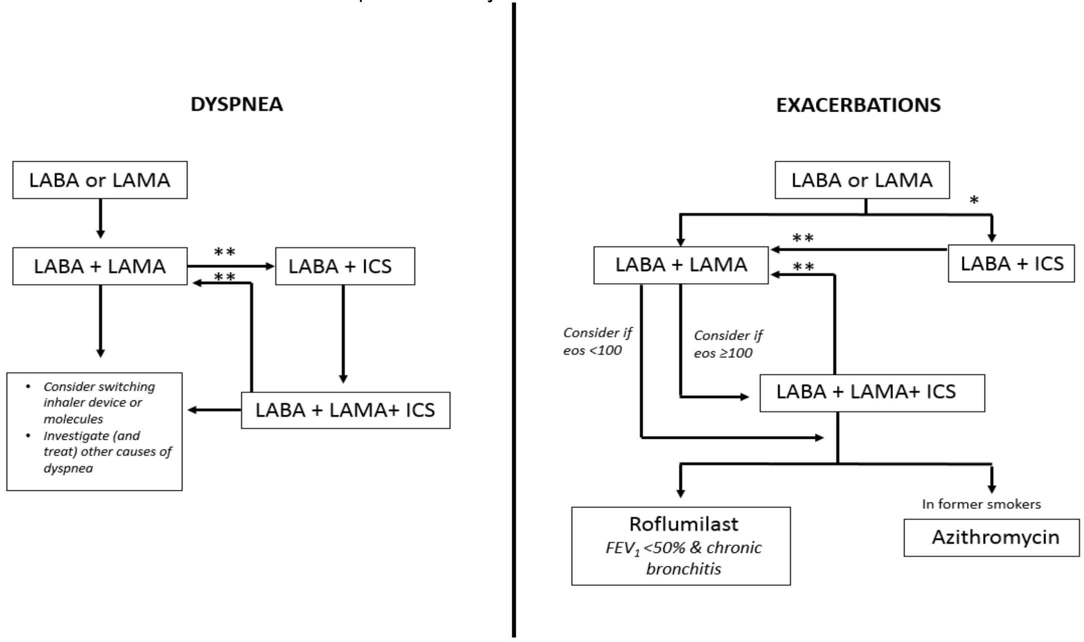


Eos= blood eosinophil count (cells/ µl)
*Consider if eos ≥ 300 or eos ≥100 AND ≥2 moderate exacerbations / 1 hospitalization
**Consider de-escalation of ICS or switch if pneumonia, inappropriate original indication or lack of response to ICS

*Global Initiative for Chronic Obstructive Lung Disease. Global strategy for the diagnosis, management, and prevention of chronic obstructive pulmonary disease 2020 report. https://goldcopd.org/wp-content/uploads/2019/11/GOLD-2020-REPORT-ver1.0wms.pdf.*

2.9. Please indicate your level of agreement regarding the following statements concerning the **use of fixed dose LAMA/LABA in COPD treatment**. *(being 1 completely disagree and 9 completely agree)*

| | 1 Completely disagree | 2 | 3 | 4 | 5 | 6 | 7 | 8 | 9 Completely agree | Don't know |
|---|---|---|---|---|---|---|---|---|---|---|
| a. LAMA/LABA improve breathlessness compared to LAMA monotherapy. | ☐ | ☐ | ☐ | ☐ | ☐ | ☐ | ☐ | ☐ | ☐ | ☐ |
| b. LAMA/LABA improve breathlessness compared to LABA/ICS. | ☐ | ☐ | ☐ | ☐ | ☐ | ☐ | ☐ | ☐ | ☐ | ☐ |
| c. LAMA should be the initial treatment for COPD maintenance therapy before LAMA/LABA. | ☐ | ☐ | ☐ | ☐ | ☐ | ☐ | ☐ | ☐ | ☐ | ☐ |
| d. For some patients, LAMA/LABA can be the initial COPD maintenance treatment. | ☐ | ☐ | ☐ | ☐ | ☐ | ☐ | ☐ | ☐ | ☐ | ☐ |
| e. LABA/ICS should be the initial treatment for COPD maintenance therapy before LAMA/LABA. | ☐ | ☐ | ☐ | ☐ | ☐ | ☐ | ☐ | ☐ | ☐ | ☐ |
| f. LABA/ICS is a better choice to prevent exacerbations compared to LAMA/LABA. | ☐ | ☐ | ☐ | ☐ | ☐ | ☐ | ☐ | ☐ | ☐ | ☐ |
| g. LAMA/LABA improve physical activity compared to LAMA monotherapy. | ☐ | ☐ | ☐ | ☐ | ☐ | ☐ | ☐ | ☐ | ☐ | ☐ |
| h. LAMA/LABA improve physical activity compared to LABA/ICS. | ☐ | ☐ | ☐ | ☐ | ☐ | ☐ | ☐ | ☐ | ☐ | ☐ |
| i. LAMA/LABA improve quality of life compared to LAMA monotherapy. | ☐ | ☐ | ☐ | ☐ | ☐ | ☐ | ☐ | ☐ | ☐ | ☐ |
| j. LAMA/LABA improve quality of life compared to LABA/ICS. | ☐ | ☐ | ☐ | ☐ | ☐ | ☐ | ☐ | ☐ | ☐ | ☐ |

## 3. RISKS-BENEFITS OF ICS TREATMENT AND OPTIMIZATION OF THEIR CLINICAL USE

***Inhaled corticosteroids (ICS) are an integral part of COPD treatment however, according to COPD guidelines, their use is reserved only for certain type of patients. Current clinical practice shows that ICS is widely used beyond its indications and are frequently selected as the first maintenance therapy.***
***The aim of this section is to reach a consensus on the risk-benefit of ICS in different COPD patient subgroups, to collect your experience with ICS withdrawal and to discuss potential solutions to optimize treatment.***

*Please, remember that in case you are* ***not familiar*** *with a procedure or* ***if in your country it is not relevant****, please feel free to mark the option* ***"Don't know"****'.*

3.1 Please indicate your level of agreement regarding the following statements in reference to the **risks and benefits of ICS treatment for COPD patients in general, and for certain type of patients**. *(being 1 completely disagree and 9 completely agree)*

| | **1 Completely disagree** | **2** | **3** | **4** | **5** | **6** | **7** | **8** | **9 Completely agree** | **Don't know** |
|---|---|---|---|---|---|---|---|---|---|---|
| **Risks- Benefits of ICS treatment** | | | | | | | | | | |
| a. When considering **ICS prescription**, I take into account: | | | | | | | | | | |
| i. Blood eosinophil count alone. | ☐ | ☐ | ☐ | ☐ | ☐ | ☐ | ☐ | ☐ | ☐ | ☐ |
| ii. Exacerbation risk alone. | ☐ | ☐ | ☐ | ☐ | ☐ | ☐ | ☐ | ☐ | ☐ | ☐ |
| iii. Combination of exacerbation risk and blood eosinophil count. | ☐ | ☐ | ☐ | ☐ | ☐ | ☐ | ☐ | ☐ | ☐ | ☐ |
| iv. Comorbidities alone. | ☐ | ☐ | ☐ | ☐ | ☐ | ☐ | ☐ | ☐ | ☐ | ☐ |
| v. Comorbidities, blood eosinophil count and exacerbation risks. | ☐ | ☐ | ☐ | ☐ | ☐ | ☐ | ☐ | ☐ | ☐ | ☐ |
| vi. Uncertainty of concomitant asthma diagnosis. | ☐ | ☐ | ☐ | ☐ | ☐ | ☐ | ☐ | ☐ | ☐ | ☐ |
| b. It is good to re-assess older ICS prescriptions to see if it is still indicated and/or efficacious. | ☐ | ☐ | ☐ | ☐ | ☐ | ☐ | ☐ | ☐ | ☐ | ☐ |
| c. Treatment with ICS increases the risk of **pneumonia.** | ☐ | ☐ | ☐ | ☐ | ☐ | ☐ | ☐ | ☐ | ☐ | ☐ |
| d. Treatment with ICS increases the risk of **tuberculosis** | ☐ | ☐ | ☐ | ☐ | ☐ | ☐ | ☐ | ☐ | ☐ | ☐ |
| e. Special attention should be given to the risk-benefit ratio | ☐ | ☐ | ☐ | ☐ | ☐ | ☐ | ☐ | ☐ | ☐ | ☐ |

| | | | | | | | | | | |
|---|---|---|---|---|---|---|---|---|---|---|
| of ICS in COPD patients with **osteopenia / fractures** | | | | | | | | | | |
| f. Special care should be taken with the prescription of ICS to a patient suffering from **osteoporosis**, since it could increase the risk for fractures by reducing bone density. | ☐ | ☐ | ☐ | ☐ | ☐ | ☐ | ☐ | ☐ | ☐ | ☐ |
| g. ICS treatment increase the risk of **diabetes**. | ☐ | ☐ | ☐ | ☐ | ☐ | ☐ | ☐ | ☐ | ☐ | ☐ |
| h. Special attention should be given to the risk/benefit ratio of ICS treatment because it has a negative impact on **diabetes control.** | ☐ | ☐ | ☐ | ☐ | ☐ | ☐ | ☐ | ☐ | ☐ | ☐ |
| i. Bruising is not uncommon in ICS-treated patients | ☐ | ☐ | ☐ | ☐ | ☐ | ☐ | ☐ | ☐ | ☐ | ☐ |
| j. Bruising negatively impacts quality of life of COPD patients treated with ICS | ☐ | ☐ | ☐ | ☐ | ☐ | ☐ | ☐ | ☐ | ☐ | ☐ |
| k. Oral candidiasis is not uncommon in ICS-treated patients | ☐ | ☐ | ☐ | ☐ | ☐ | ☐ | ☐ | ☐ | ☐ | ☐ |
| l. Oral candidiasis negatively impacts quality of life of COPD patients treated with ICS | ☐ | ☐ | ☐ | ☐ | ☐ | ☐ | ☐ | ☐ | ☐ | ☐ |
| m. **Long-term ICS treatment in patients with low blood eosinophil count increases the risk of exacerbations** probably linked with changes in the airway microbiome. | ☐ | ☐ | ☐ | ☐ | ☐ | ☐ | ☐ | ☐ | ☐ | ☐ |
| n. ICS side effects could be more prominent in patients with COPD who have **no history of asthma**. | ☐ | ☐ | ☐ | ☐ | ☐ | ☐ | ☐ | ☐ | ☐ | ☐ |
| o. Treatment with ICS is less beneficial when patients are **smokers.** | ☐ | ☐ | ☐ | ☐ | ☐ | ☐ | ☐ | ☐ | ☐ | ☐ |
| p. **LABA/ICS may decrease exacerbations to a greater extent than a LABA/LAMA** combination for patients with: | | | | | | | | | | |
| i. **High** exacerbation risk (>2 exacerbations and/or 1 hospitalization in the previous year) & **high** eosinophil blood concentrations | ☐ | ☐ | ☐ | ☐ | ☐ | ☐ | ☐ | ☐ | ☐ | ☐ |

| | | | | | | | | | | |
|---|---|---|---|---|---|---|---|---|---|---|
| ii. **High** exacerbation risk & **low** eosinophil blood concentrations | ☐ | ☐ | ☐ | ☐ | ☐ | ☐ | ☐ | ☐ | ☐ | ☐ |
| iii. **Low** exacerbation risk & **high** eosinophil blood concentrations | ☐ | ☐ | ☐ | ☐ | ☐ | ☐ | ☐ | ☐ | ☐ | ☐ |
| iv. **Low** exacerbation risk & **low** eosinophil blood concentrations | ☐ | ☐ | ☐ | ☐ | ☐ | ☐ | ☐ | ☐ | ☐ | ☐ |
| q. Co-morbidities should be considered when selecting COPD treatment | ☐ | ☐ | ☐ | ☐ | ☐ | ☐ | ☐ | ☐ | ☐ | ☐ |

3.2 According to the **GOLD Report**, **ICS de-escalation** can be considered in case of pneumonia or inappropriate original indication or lack of response to ICS. Please indicate **how confident you are in the next situations** *(being 1 not confident at all and 9 completely confident)*

| | **Not confident at all** | | | | | | | | **Completely confident** | **Don't know** |
|---|---|---|---|---|---|---|---|---|---|---|
| | **1** | **2** | **3** | **4** | **5** | **6** | **7** | **8** | **9** | |
| Confidence with… | | | | | | | | | | |
| a. With ICS withdrawal in case of pneumonia | | | | | | | | | | |
| b. With withdrawal in case of no longer indication for ICS | | | | | | | | | | |
| c. With ICS withdrawal in case of lack of response to ICS | | | | | | | | | | |

3.3 For what reasons do you think ICS-treated patients can be eligible for ICS withdrawal? *(multiple choice, please check all that apply)*

- Patients with pneumonia
- No longer indication for ICS
- Lack of response to ICS
- Patients without asthma
- Patients with diabetes
- Patients with osteoporosis/osteopenia
- Patients with cardiovascular disease
- Patients with ICS-related side effects (e.g. horse voice, bruising of the skin, oral candidiasis)
- Patients should never be withdrawn from an ICS treatment.
- Other, please specify: ______________

3.4 Among your [show answer from **(Q1.7)*(2)**] COPD patients **seen in the last year**, what percentage have been or are currently being treated with ICS, approximately?
____%

3.5 What percentage of your [show answer from (((**Q1.7)** * (2) )* **(Q3.4)**)/100] **ICS-treated patients** seen in the **last year** do you think are eligible for ICS withdrawal, approximately?

____%

3.6 Have you **ever withdrawn ICS treatment** from any COPD patient? (*single answer)*

- ❑ Yes
- ❑ No

**If "no", questions 3.6.1to 3.6.5 will be skipped*
**If yes:*

3.6.1 From what percentage of your [show answer from ((**(Q1.7)** * (2) )* **(Q3.4**))/ (100)] ICS-treated patients, have you withdrawn ICS treatment in the **last year,** approximately?

____%

**If 3.6.1=0, 3.6.3-3.6.5 will be skipped*

3.6.2 What percentage of your [show answer from ((Q1.7*2)* Q3.4)/100] COPD ICS-treated patients have **refused** the proposed ICS withdrawal in the **last year,** approximately?

_____%

3.6.3 Could you please distribute approximately those COPD patients from whom you have withdrawn ICS treatment in the **last year**, according to the **reason for withdrawal**?

| | | |
|---|---|---|
| a. | Lack of response to ICS | % |
| b. | No longer indication for ICS | % |
| c. | Pneumonia | % |
| d. | Fear of adverse events to ICS | % |
| e. | Other, please specify:______ | % |

**the total must sum 100%*

3.6.4 What percentage of those COPD patients from whom you have withdrawn ICS treatment in the **last year** remained **with no exacerbations within 6 months** after ICS withdrawal, approximately?

______%

3.6.5 In which percentage of those COPD patients from whom you have withdrawn ICS treatment in the **last year** did you **need to reintroduce ICS within 6 months** after withdrawal of ICS, approximately?

____%

**If >0:*

3.6.5.a Could you please distribute approximately the reasons for ICS re-introduction in those patients?

| | |
|---|---|
| f. Patient preference | % |
| g. Increased symptoms | % |
| h. Exacerbation | % |
| i. Other, please specify: _______ | % |

3.7 In your personal opinion, how **should** ICS be withdrawn in patients on **LABA/ICS** therapy? *(multiple choice)*

- o Gradual ICS dose reduction without adding any other treatment in all patients.
- o Gradual ICS dose reduction without adding any other treatment in patients on high dose ICS.
- o Abrupt ICS withdrawal without adding any other treatment.
- o Gradual ICS dose reduction and LAMA/LABA treatment introduction at the same time.
- o Gradual ICS dose reduction and LAMA/LABA treatment once ICS is completely withdrawn.
- o Abrupt ICS withdrawal and LAMA/LABA treatment introduction.
- o ICS should not be withdrawn from patients on LABA/ICS.
- o Other, please specify: ______________

3.8 In your personal opinion, how **should** ICS be withdrawn in patients on **triple therapy (LAMA/LABA/ICS)**? *(multiple choice)*

- o Gradual ICS dose reduction maintaining LAMA/LABA combination.
- o Abrupt ICS withdrawal maintaining LAMA/LABA combination.
- o ICS should never be withdrawn from patients on LAMA/LABA/ICS.
- o Other, please specify: ____________

3.9 In your personal opinion, what drives the choice of the LAMA-LABA combination when withdrawing ICS in patients who were on **triple therapy (LAMA/LABA/ICS)**? *(multiple choice)*

- Same device
- Same LAMA
- Same LABA
- Switch to a potentially more effective LAMA
- Switch to a potentially more effective LABA
- Other, please specify: ____________

3.10 In your personal opinion, after ICS withdrawal, how **should** patients with COPD be monitored? *(multiple choice)*

- No specific follow-up.
- Planned follow-up visit / call 1 month after ICS withdrawal.
- Planned visit for spirometry.
- Patients should have the possibility to communicate with me or another healthcare professional in case of questions.
- Patients should have the possibility to call me or another healthcare professional in case of complaints.
- Other, please specify: _________

3.11 Please mark the reasons that could indicate the need for reintroducing ICS *(multiple choice)*:

- Worsening of COPD symptoms (such breathlessness…)
- Exacerbations after ICS withdrawal.
- Persistent adverse events after ICS withdrawal.
- Significant worsening of spirometry.
- Patient preference.
- Other, please, specify: ______

3.12 What should be done in such cases? (*single answer)*

- ❑ Back to the ICS treatment at the same doses than before.
- ❑ Back to the ICS treatment at lower doses than before.
- ❑ Keep the ICS withdrawal.
- ❑ Other, please, specify: ______

3.13 Please indicate your level of agreement with the following statements in relation to **the current use of inhaled corticosteroids in COPD patients in your country.** *(being 1 completely disagree and 9 completely agree)*

| | 1 Completely disagree | 2 | 3 | 4 | 5 | 6 | 7 | 8 | 9 Completely agree | Don't know |
|---|---|---|---|---|---|---|---|---|---|---|
| a. Physicians are familiar with international recommendations regarding ICS treatment restrictions. | ☐ | ☐ | ☐ | ☐ | ☐ | ☐ | ☐ | ☐ | ☐ | ☐ |
| b. Local and national guidelines are timely updated according to latest evidence. | ☐ | ☐ | ☐ | ☐ | ☐ | ☐ | ☐ | ☐ | ☐ | ☐ |
| c. ICS are not over-prescribed in the primary care setting. | ☐ | ☐ | ☐ | ☐ | ☐ | ☐ | ☐ | ☐ | ☐ | ☐ |
| d. ICS are not over-prescribed in the specialty care. | ☐ | ☐ | ☐ | ☐ | ☐ | ☐ | ☐ | ☐ | ☐ | ☐ |
| e. There is enough time in daily clinical practice to... | | | | | | | | | | |
| i. To assess factors that indicate if ICS are adequate for treatment initiation. | ☐ | ☐ | ☐ | ☐ | ☐ | ☐ | ☐ | ☐ | ☐ | ☐ |
| ii. To assess factors that indicate that continuation of ICS is still adequate as maintenance treatment. | ☐ | ☐ | ☐ | ☐ | ☐ | ☐ | ☐ | ☐ | ☐ | ☐ |
| f. There is not enough evidence supporting ICS withdrawal… | | | | | | | | | | |
| i. For COPD patients with co-morbidities. | ☐ | ☐ | ☐ | ☐ | ☐ | ☐ | ☐ | ☐ | ☐ | ☐ |
| ii. For patients with low blood eosinophil count (<100 cells/µl). | ☐ | ☐ | ☐ | ☐ | ☐ | ☐ | ☐ | ☐ | ☐ | ☐ |
| iii. For patients with low/medium blood eosinophil count (100-300 cells/µl). | ☐ | ☐ | ☐ | ☐ | ☐ | ☐ | ☐ | ☐ | ☐ | ☐ |
| iv. For patients with high blood eosinophil count (>300 cells/µl). | ☐ | ☐ | ☐ | ☐ | ☐ | ☐ | ☐ | ☐ | ☐ | ☐ |
| g. There is not enough evidence on the benefits of ICS withdrawal. | ☐ | ☐ | ☐ | ☐ | ☐ | ☐ | ☐ | ☐ | ☐ | ☐ |
| h. Pulmonologists feel insecure with ICS withdrawal. | ☐ | ☐ | ☐ | ☐ | ☐ | ☐ | ☐ | ☐ | ☐ | ☐ |

| | | | | | | | | | | |
|---|---|---|---|---|---|---|---|---|---|---|
| i. GPs feel insecure with ICS withdrawal. | ☐ | ☐ | ☐ | ☐ | ☐ | ☐ | ☐ | ☐ | ☐ | ☐ |
| j. The health care system facilitates ICS prescription in comparison to other treatments (e.g. reimbursement conditions). | ☐ | ☐ | ☐ | ☐ | ☐ | ☐ | ☐ | ☐ | ☐ | ☐ |

3.14 Please, indicate your level of agreement regarding the following recommendations about **treatment switch or ICS withdrawal in COPD patients.** *(being 1 completely disagree and 9 completely agree)*.

| | 1 Completely disagree | 2 | 3 | 4 | 5 | 6 | 7 | 8 | 9 Completely agree | Don't know |
|---|---|---|---|---|---|---|---|---|---|---|
| **Switching from both LABA/ICS or LAMA/LABA/ICS to LABA/LAMA should be considered if…** | | | | | | | | | | |
| a. ICS therapy is no longer indicated. | ☐ | ☐ | ☐ | ☐ | ☐ | ☐ | ☐ | ☐ | ☐ | ☐ |
| b. there has not been a therapeutic response to ICS treatment. | ☐ | ☐ | ☐ | ☐ | ☐ | ☐ | ☐ | ☐ | ☐ | ☐ |
| c. history or current pneumonia | ☐ | ☐ | ☐ | ☐ | ☐ | ☐ | ☐ | ☐ | ☐ | ☐ |
| d. there are other side effects possibly related to ICS (like osteoporosis, cataract…) | ☐ | ☐ | ☐ | ☐ | ☐ | ☐ | ☐ | ☐ | ☐ | ☐ |
| **ICS discontinuation should be considered in patients with:** | | | | | | | | | | |
| **e. No therapeutic response, …** | | | | | | | | | | |
| i. even if eosinophil count ≥ 300 cells /µl | ☐ | ☐ | ☐ | ☐ | ☐ | ☐ | ☐ | ☐ | ☐ | ☐ |
| ii. even if eosinophil count is 100-300 cells/µl | ☐ | ☐ | ☐ | ☐ | ☐ | ☐ | ☐ | ☐ | ☐ | ☐ |
| **f. Adverse events, …** | | | | | | | | | | |
| i. even if eosinophil count ≥ 300 cells /µl | ☐ | ☐ | ☐ | ☐ | ☐ | ☐ | ☐ | ☐ | ☐ | ☐ |
| ii. even if eosinophil count is 100-300 cells/µl | ☐ | ☐ | ☐ | ☐ | ☐ | ☐ | ☐ | ☐ | ☐ | ☐ |
| **g. Diabetes, …** | | | | | | | | | | |
| i. even if eosinophil count ≥ 300 cells /µl | ☐ | ☐ | ☐ | ☐ | ☐ | ☐ | ☐ | ☐ | ☐ | ☐ |

| | | | | | | | | | | |
|---|---|---|---|---|---|---|---|---|---|---|
| ii. even if eosinophil count is 100-300 cells/µl | ☐ | ☐ | ☐ | ☐ | ☐ | ☐ | ☐ | ☐ | ☐ | ☐ |
| **h. Osteoporosis…** | | | | | | | | | | |
| i. even if eosinophil count ≥ 300 cells /µl | ☐ | ☐ | ☐ | ☐ | ☐ | ☐ | ☐ | ☐ | ☐ | ☐ |
| ii. even if eosinophil count is 100-300 cells/µl | ☐ | ☐ | ☐ | ☐ | ☐ | ☐ | ☐ | ☐ | ☐ | ☐ |
| i. **Smoker patients…** | | | | | | | | | | |
| i. even if eosinophil count ≥ 300 cells /µl | ☐ | ☐ | ☐ | ☐ | ☐ | ☐ | ☐ | ☐ | ☐ | ☐ |
| ii. even if eosinophil count is 100-300 cells/µl | ☐ | ☐ | ☐ | ☐ | ☐ | ☐ | ☐ | ☐ | ☐ | ☐ |
| j. **Patient with low eosinophil count (<100 cells /µl)** | | | | | | | | | | |
| i. even if they suffer 1 moderate exacerbation a year | ☐ | ☐ | ☐ | ☐ | ☐ | ☐ | ☐ | ☐ | ☐ | ☐ |
| ii. even if they suffer 1 severe exacerbation with hospitalization a year | ☐ | ☐ | ☐ | ☐ | ☐ | ☐ | ☐ | ☐ | ☐ | ☐ |

## 4. OPTIMISATION OF COPD PATIENT MANAGEMENT AND TREATMENT

4.1 Please indicate your level of agreement regarding the following statements in relation to **the different items/resources that could help physicians optimize the COPD patient management and treatment.** *(being 1 completely disagree and 9 completely agree)*

| | 1 Completely disagree | 2 | 3 | 4 | 5 | 6 | 7 | 8 | 9 Completely agree | Don't know |
|---|---|---|---|---|---|---|---|---|---|---|
| a. Increase patient consultation time. | ☐ | ☐ | ☐ | ☐ | ☐ | ☐ | ☐ | ☐ | ☐ | ☐ |
| b. Integrate a tool to assess comorbidities and drug interactions in a medical software to **trigger alerts.** | ☐ | ☐ | ☐ | ☐ | ☐ | ☐ | ☐ | ☐ | ☐ | ☐ |
| c. Have access to a patient support program that helps COPD patients to: | | | | | | | | | | |
| i. Stop smoking. | ☐ | ☐ | ☐ | ☐ | ☐ | ☐ | ☐ | ☐ | ☐ | ☐ |
| ii. Identify their symptoms and report them to the HCP. | ☐ | ☐ | ☐ | ☐ | ☐ | ☐ | ☐ | ☐ | ☐ | ☐ |
| iii. Cope with their illness. | ☐ | ☐ | ☐ | ☐ | ☐ | ☐ | ☐ | ☐ | ☐ | ☐ |
| iv. Receive more information about their illness. | ☐ | ☐ | ☐ | ☐ | ☐ | ☐ | ☐ | ☐ | ☐ | ☐ |
| d. Faster updates of local guidelines. | ☐ | ☐ | ☐ | ☐ | ☐ | ☐ | ☐ | ☐ | ☐ | ☐ |
| e. Align the different COPD-guidelines. | ☐ | ☐ | ☐ | ☐ | ☐ | ☐ | ☐ | ☐ | ☐ | ☐ |
| f. Have access to a validated tool that helps HCPs identify patients who would benefit from ICS vs. those in whom it may be inappropriate. | ☐ | ☐ | ☐ | ☐ | ☐ | ☐ | ☐ | ☐ | ☐ | ☐ |
| g. A guidance tool on how to withdraw ICS in each type of patient. | ☐ | ☐ | ☐ | ☐ | ☐ | ☐ | ☐ | ☐ | ☐ | ☐ |
| h. Incorporate recommendations for the treatment and management of the multi-morbid COPD patient in the current guidelines. | ☐ | ☐ | ☐ | ☐ | ☐ | ☐ | ☐ | ☐ | ☐ | ☐ |
| i. Specifically train physicians on the | ☐ | ☐ | ☐ | ☐ | ☐ | ☐ | ☐ | ☐ | ☐ | ☐ |

| | | | | | | | | | | |
|---|---|---|---|---|---|---|---|---|---|---|
| management of COPD patients. | | | | | | | | | | |
| j. Improvement of doctor-patient communication that helps the physician discuss patient preference related to treatment (shared decision) | ☐ | ☐ | ☐ | ☐ | ☐ | ☐ | ☐ | ☐ | ☐ | ☐ |
| k. Collaboration/ support to GPs from pulmonologists. | ☐ | ☐ | ☐ | ☐ | ☐ | ☐ | ☐ | ☐ | ☐ | ☐ |
| l. Optimize the referral from GPs to specialists | ☐ | ☐ | ☐ | ☐ | ☐ | ☐ | ☐ | ☐ | ☐ | ☐ |
| m. Decrease waiting time regarding referral from GPs to specialists | ☐ | ☐ | ☐ | ☐ | ☐ | ☐ | ☐ | ☐ | ☐ | ☐ |

## 5. POTENTIAL IMPACT OF COPD TREATMENT OPTIMIZATION

5.1. Please, indicate your level of agreement regarding the following statements in reference to the **potential clinical impact of COPD treatment optimization** (*being 1 completely disagree and 9 completely agree)*

| | 1 Completely disagree | 2 | 3 | 4 | 5 | 6 | 7 | 8 | 9 Completely agree | Don't know |
|---|---|---|---|---|---|---|---|---|---|---|
| **Clinical impact** | | | | | | | | | | |
| a. **Reducing the use of non-indicated ICS in COPD patients could result in…** | | | | | | | | | | |
| i. A reduction of adverse events such as infections. | ☐ | ☐ | ☐ | ☐ | ☐ | ☐ | ☐ | ☐ | ☐ | ☐ |
| ii. A better outcome of co-existing diseases. | ☐ | ☐ | ☐ | ☐ | ☐ | ☐ | ☐ | ☐ | ☐ | ☐ |
| iii. A reduction of bacterial exacerbations. | ☐ | ☐ | ☐ | ☐ | ☐ | ☐ | ☐ | ☐ | ☐ | ☐ |
| iv. Worsening of quality of life. | ☐ | ☐ | ☐ | ☐ | ☐ | ☐ | ☐ | ☐ | ☐ | ☐ |
| v. Improvement of treatment adherence. | ☐ | ☐ | ☐ | ☐ | ☐ | ☐ | ☐ | ☐ | ☐ | ☐ |
| b. **Increasing the use of LABA/LAMA in patients for whom LABA/ICS is not indicated could result in…** | | | | | | | | | | |
| i. A better control of COPD | ☐ | ☐ | ☐ | ☐ | ☐ | ☐ | ☐ | ☐ | ☐ | ☐ |
| ii. Increased clinical response to treatment. | ☐ | ☐ | ☐ | ☐ | ☐ | ☐ | ☐ | ☐ | ☐ | ☐ |
| iii. Reduced exacerbations | ☐ | ☐ | ☐ | ☐ | ☐ | ☐ | ☐ | ☐ | ☐ | ☐ |
| iv. Increased adverse events | ☐ | ☐ | ☐ | ☐ | ☐ | ☐ | ☐ | ☐ | ☐ | ☐ |
| c. **In patients with further exacerbations with high eosinophil count, stepping up the inhaled treatment to LABA/LAMA/ICS (triple therapy) from LABA/LAMA may…** | | | | | | | | | | |
| i. improve lung function | ☐ | | | | | | | | ☐ | ☐ |
| ii. increase adverse events (such as infections) | ☐ | | | | | | | | ☐ | ☐ |
| iii. prevent exacerbations | ☐ | | | | | | | | ☐ | ☐ |
| iv. Control COPD better | ☐ | | | | | | | | ☐ | ☐ |
| v. Increase clinical response to treatment. | ☐ | | | | | | | | ☐ | ☐ |

5.2. Are you aware of the pricing of the different treatment classes (LAMA, LABA, LABA/ICS, LAMA/LABA, LAMA/LABA/ICS) in your country? (*single answer)*
- ❑ Yes
- ❑ No

5.3. Please, indicate your level of agreement regarding the following statements in reference to the **economic impact of COPD treatment.** (*being 1 completely disagree and 9 completely agree)*

| | 1 Completely disagree | 2 | 3 | 4 | 5 | 6 | 7 | 8 | 9 Completely agree | Don't know |
|---|---|---|---|---|---|---|---|---|---|---|
| a. At same perceived clinical added value, I prescribe the cheapest product. | ☐ | ☐ | ☐ | ☐ | ☐ | ☐ | ☐ | ☐ | ☐ | ☐ |
| b. Medicines represent the most significant source of costs in management of COPD patients (which also includes visits to healthcare professionals, exacerbations, pneumonia). | ☐ | ☐ | ☐ | ☐ | ☐ | ☐ | ☐ | ☐ | ☐ | ☐ |
| c. I am sensitive to information on the financial implications of COPD management on the healthcare expenditure. | ☐ | ☐ | ☐ | ☐ | ☐ | ☐ | ☐ | ☐ | ☐ | ☐ |
| d. Pneumonia represents a financial burden for healthcare expenditures. | ☐ | ☐ | ☐ | ☐ | ☐ | ☐ | ☐ | ☐ | ☐ | ☐ |
| e. Exacerbations represent a financial burden for healthcare expenditures. | ☐ | ☐ | ☐ | ☐ | ☐ | ☐ | ☐ | ☐ | ☐ | ☐ |

## 6. IMPORTANCE OF THE INHALER

6.1. When selecting an inhaler for your patient please indicate the importance of patient or device characteristics you might consider, **independently of the active ingredients.** (*being 1 not important and 9 very important)*

| | 1 Not important | 2 | 3 | 4 | 5 | 6 | 7 | 8 | 9 Very important | Don't know |
|---|---|---|---|---|---|---|---|---|---|---|
| **Patient characteristics** | | | | | | | | | | |
| a. Age of the patient | ☐ | ☐ | ☐ | ☐ | ☐ | ☐ | ☐ | ☐ | ☐ | ☐ |
| b. Severity of the disease | ☐ | ☐ | ☐ | ☐ | ☐ | ☐ | ☐ | ☐ | ☐ | ☐ |
| c. Hand dexterity | ☐ | ☐ | ☐ | ☐ | ☐ | ☐ | ☐ | ☐ | ☐ | ☐ |
| d. Patient literacy/ability to understand medical information / Instruction on inhaler use | ☐ | ☐ | ☐ | ☐ | ☐ | ☐ | ☐ | ☐ | ☐ | ☐ |
| e. Patient preference for the device | ☐ | ☐ | ☐ | ☐ | ☐ | ☐ | ☐ | ☐ | ☐ | ☐ |
| f. Patient inspiratory flow rate (measured or estimated) | ☐ | ☐ | ☐ | ☐ | ☐ | ☐ | ☐ | ☐ | ☐ | ☐ |
| **Device characteristics** | | | | | | | | | | |
| g. Level of lung deposition | ☐ | ☐ | ☐ | ☐ | ☐ | ☐ | ☐ | ☐ | ☐ | ☐ |
| h. Size and shape | ☐ | ☐ | ☐ | ☐ | ☐ | ☐ | ☐ | ☐ | ☐ | ☐ |
| i. Multi-dose | ☐ | ☐ | ☐ | ☐ | ☐ | ☐ | ☐ | ☐ | ☐ | ☐ |
| j. Easy coordination of actuation and inhalation | ☐ | ☐ | ☐ | ☐ | ☐ | ☐ | ☐ | ☐ | ☐ | ☐ |
| k. Easy inhalation / low inspiratory effort | ☐ | ☐ | ☐ | ☐ | ☐ | ☐ | ☐ | ☐ | ☐ | ☐ |
| l. Re-usability (use of refills enabling longer use of device) | ☐ | ☐ | ☐ | ☐ | ☐ | ☐ | ☐ | ☐ | ☐ | ☐ |
| m. Presence of dose counter | ☐ | ☐ | ☐ | ☐ | ☐ | ☐ | ☐ | ☐ | ☐ | ☐ |
| n. Locking mechanism when empty | ☐ | ☐ | ☐ | ☐ | ☐ | ☐ | ☐ | ☐ | ☐ | ☐ |
| o. Propellant-free | ☐ | ☐ | ☐ | ☐ | ☐ | ☐ | ☐ | ☐ | ☐ | ☐ |
| p. Lack of additives (e.g. lactose) | ☐ | ☐ | ☐ | ☐ | ☐ | ☐ | ☐ | ☐ | ☐ | ☐ |
| q. Low carbon footprint | ☐ | ☐ | ☐ | ☐ | ☐ | ☐ | ☐ | ☐ | ☐ | ☐ |

6.2. Please, indicate the devices that you think match the following statements (*more than one answer per row is possible)*:

| | DPI (dry powder inhaler) | pMDI (pressured metered dose inhaler) | Soft mist inhaler | Don't know |
|---|---|---|---|---|
| a. High level of lung deposition | ☐ | ☐ | ☐ | ☐ |
| b. Independent of the patient's inspiratory flow rate | ☐ | ☐ | ☐ | ☐ |
| c. Effective when patient is limited in inspiratory capacity (e.g. during exacerbation) | ☐ | ☐ | ☐ | ☐ |
| d. Propellant-free | ☐ | ☐ | ☐ | ☐ |
| e. Low carbon footprint | ☐ | ☐ | ☐ | ☐ |
| f. Reusable | ☐ | ☐ | ☐ | ☐ |
| g. I am most experienced with… | ☐ | ☐ | ☐ | ☐ |
| h. Easy for the patient to use | ☐ | ☐ | ☐ | ☐ |
| i. High patient satisfaction | ☐ | ☐ | ☐ | ☐ |
| j. Easy coordination | ☐ | ☐ | ☐ | ☐ |
